# A High-Resolution Map of *Arabidopsis* Recombinant Inbred Lines by Whole-Genome Exon Array Hybridization

**DOI:** 10.1371/journal.pgen.0020144

**Published:** 2006-09-15

**Authors:** Tatjana Singer, Yiping Fan, Hur-Song Chang, Tong Zhu, Samuel P Hazen, Steven P Briggs

**Affiliations:** 1 Torrey Mesa Research Institute, Syngenta Research and Technology, San Diego, California, United States of America; 2 St. Jude Children's Research Hospital, Hartwell Center for Bioinformatics and Biotechnology, Memphis, Tennessee, United States of America; 3 DermTech International, La Jolla, California, United States of America; 4 Syngenta Biotechnology, Research Triangle Park, North Carolina, United States of America; 5 The Scripps Research Institute, La Jolla, California, United States of America; 6 Division of Biological Sciences, University of California San Diego, La Jolla, California, United States of America; University of Wisconsin, United States of America

## Abstract

Recombinant populations were the basis for Mendel's first genetic experiments and continue to be key to the study of genes, heredity, and genetic variation today. Genotyping several hundred thousand loci in a single assay by hybridizing genomic DNA to oligonucleotide arrays provides a powerful technique to improve precision linkage mapping. The genotypes of two accessions of *Arabidopsis* were compared by using a 400,000 feature exon-specific oligonucleotide array. Around 16,000 single feature polymorphisms (SFPs) were detected in ~8,000 of the ~26,000 genes represented on the array. Allelic variation at these loci was measured in a recombinant inbred line population, which defined the location of 815 recombination breakpoints. The genetic linkage map had a total length of 422.5 cM, with 676 informative SFP markers representing intervals of ~0.6 cM. One hundred fifteen single gene intervals were identified. Recombination rate, SFP distribution, and segregation in this population are not uniform. Many genomic regions show a clustering of recombination events including significant hot spots. The precise haplotype structure of the recombinant population was defined with unprecedented accuracy and resolution. The resulting linkage map allows further refinement of the hundreds of quantitative trait loci identified in this well-studied population. Highly variable recombination rates along each chromosome and extensive segregation distortion were observed in the population.

## Synopsis

A goal of many genetic studies is to discover the underlying genetic condition (the genotype) of a specific physical manifestation in an organism (the phenotype), such as diabetes in humans or leaf rust in cultivated wheat. A limitation to making such discoveries is the ability to resolve genotype. Gene arrays carry representations of the genome, called features, at high-density on a surface the size of a thumbnail. In this study, microarrays designed to measure gene expression were used to detect DNA sequence polymorphisms. DNA from two different *Arabidopsis* strains was hybridized to arrays representing nearly the entire coding region of the genome. Differences in hybridization intensity indicated differences in DNA sequence. The sequence differences, termed single feature polymorphisms, were then assayed in a population of 100 plants derived through inbreeding the progeny from the two parental strains. The precise location of the genetic recombination breakpoints was defined for each line. As a result, Singer et al. were able to generate one of the first very high-resolution genotyping data sets in a multicellular organism that allowed the construction of a high-resolution genetic map of *Arabidopsis*. This map will greatly facilitate attempts to make definitive associations between genotypes and phenotypes.

## Introduction

A key discovery of classical genetics was the observation that some phenotypes do not segregate independently and are thus physically linked, making it possible to map a gene to a location on a chromosome. In an organism with an annotated genome sequence, linkage analysis goes beyond associating traits and discrete molecular markers: the molecular markers and traits co-segregate with known and characterized genomic regions. Linkage mapping resolution, that is, the size of the region confidently associated with a trait, is a function of marker density, recombination rate, and the proportion of the phenotypic variation due to genetic factors. An increase in any one of these factors can improve resolution. Technological advances have made it possible to genotype several hundred thousand loci in a single assay by hybridizing DNA to a high-density oligonucleotide array. This approach has been reported in yeast [1], *Plasmodium* [2], *Anopheles* [3], human [4], and *Arabidopsis* [5]. The underlying principle of detecting sequence polymorphisms by using an oligonucleotide array is based on the observation that mismatched target DNA hybridizes with less affinity than a perfectly matched target to an oligonucleotide feature on an array, resulting in weaker signal intensity. Each oligonucleotide feature that exhibits a significant reduction in hybridization intensity, referred to as a single feature polymorphism (SFP), functions as a marker [5]. When integrated with a completely sequenced genome, the exact genomic location of each feature is known, thus adding to the utility of the marker.

In a comparison between the *Arabidopsis* accession Landsberg *erecta* (L*er*) and the reference strain Columbia (Col), ~4,000 SFPs were identified at a 5% false discovery rate (FDR) by using an *Arabidopsis* genome array that consisted of ~8,300 probe sets corresponding to ~7,000 genes [5]. The Affymetrix ATH1 array, designed to detect ~26,000 transcripts, allowed identification of more than 8,000 SFPs between various accessions (L*er*, Kas-1, Lz-0, Bur-0, and Nd-1) and Col [6–9]. Genotyping by hybridizing genomic DNA to oligonucleotide arrays (also referred to as array genotyping) has proven to be particularly well-suited for bulk segregant analysis, where phenotype-based pools of individuals from a segregating population, e.g. recombinant inbred lines (RILs) or F_2_s are assayed collectively [5–7,9,10]. Because pools of individuals are assayed, the usefulness of the genotyping data is restricted to the study at hand, and is therefore fleeting.

A lasting and far-reaching approach is to genotype independently each individual in a segregating population, preferably comprised of fixed recombinants such as RILs, which are derived from successive generation of self-pollination of progeny derived from a cross between two inbred lines. After eight generations of inbreeding, *Arabidopsis* lines should be nearly homozygous (99.2%) [11]. Thus, each RIL is a mosaic of both parental genomes in which recombination events have been fixed. Advantageously, a population of RILs represents a permanent mapping population that needs to be genotyped only once, but may be repeatedly phenotyped, a practice amenable to accurately measure quantitative phenotypes [12]. One such population was derived from a cross of the *Arabidopsis* accessions Col and L*er* followed by eight generations of inbreeding [13]. In addition to being extensively phenotyped, this population has been thoroughly genotyped using several types of molecular markers: restriction fragment length polymorphisms [13,14], cleaved amplified polymorphic sequences [15,16], simple sequence length polymorphisms [17], amplified fragment length polymorphisms [18,19], and single nucleotide polymorphisms (SNPs) [20]. The genetic linkage map that was constructed from 237 SNP markers [20] resulted in an average resolution greater than 3.5 cM, whereas the largest gap between markers was approximately 15 cM. The most recent integrated map dates from May 2001 (http://arabidopsis.info/new_ri_map.html) and was constructed by placing new markers into existing framework markers by linkage analysis. This marker set is limited by more than 5% missing genotypes for over 80% of all available markers (*n* = 1,357). These include, for example, all SNP markers that were scored in 68 of the 100 RILs [20]. In addition, many markers map to multiple positions in the genome, rendering uncertain the precise location of the marker map-position. Inaccuracies and missing marker data limit mapping resolution and usually result in statistical support of large intervals that consist of hundreds or thousands of genes, thus hampering candidate gene identification.

The aim of this study was to generate a high-density genetic linkage map and describe phenomena such as frequency and distribution of recombination that influence mapping as a gene discovery tool. We first used an exon-specific whole-genome array to identify a large number of significant SFP markers between the parental accessions Col and L*er*. These enabled us to measure variation of gene copy number and the distribution and density of SFPs across the genome. We subsequently identified 815 recombination breakpoints by genotyping 100 Col/L*er* RILs. Further, we defined the exact location of the genes that border each genetic interval. Because SFP marker density greatly exceeds the number of recombination events in this population, only the number of recombination breakpoints, rather than marker density or genotyping information, limits the resolution of the resulting linkage map. The detailed information derived from the high-resolution genotyping of the recombinant population enabled the characterization of recombination hot spots and measurement of widespread segregation distortion.

## Results

### Single Feature and Gene Copy Polymorphisms

**Figure 1:**
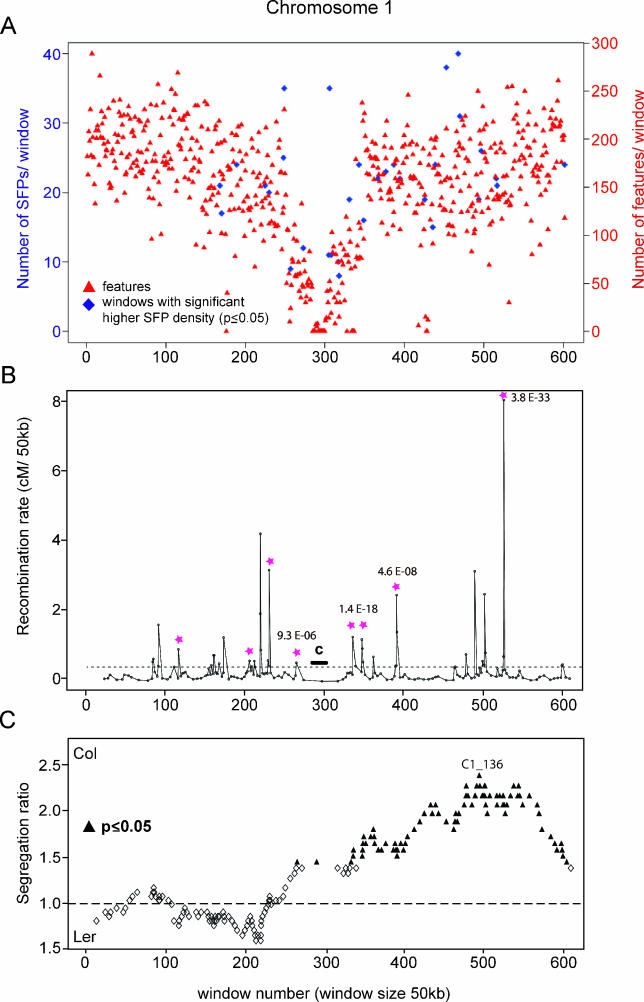
Chromosome-Wide Distribution of SFPs, Recombination Rate, and Segregation Ratio Figures for the remaining four chromosomes are included in Supporting Information. (A) Feature and SFP-distribution on Chromosome 1. The chromosome was divided into 50-kb windows. Red triangles represent number of features per window. The number of SFPs per feature in each window was compared to the number of SFPs per feature in all windows using Fisher's exact test. A *p*-value was calculated and a Bonferroni multiple testing correction was applied to test for significance. Blue diamonds indicate windows with significantly higher SFP density (*p*-value ≤ 0.05). (B) Variation of recombination rate along Chromosome 1. Recombination rate was calculated as the genetic distance (in cM/50 kb) between pairs of neighboring informative SFP markers and plotted versus the average physical distance between the same markers. Pink stars indicate hot spots of recombination that exceed the expected recombination rate significantly (*p*-value ≤ 0.001, Chi-square test, after Bonferroni-correction). *P*-values are depicted next to the peaks. All values were normalized to 50 kb. Average genome-wide recombination rate is marked as a dotted horizontal line. The location of the centromere is marked with a black bar. (C) Segregation distortion of SFP markers on Chromosome 1. Segregation ratios of genotypes for each informative SFP marker were calculated across 98 RILs and plotted along the chromosome. The vertical scale shows allele ratios. The expected equal distribution of Col and L*er* alles accross 98 lines should result in a ratio of 1 and is depicted as a dotted horizontal line. SFP markers with allele ratios above the line indicate segregation distortion towards the Col allele, SFP markers with allele ratios below the line indicate segregation distortion towards the L*er* allele. Empty diamonds represent SFP markers with no significant segregation distortion from the expected ratio of 1 between Col and L*er* genotypes. Filled triangles represent markers that show a significant (*p* ≤ 0.05, Fisher's exact test) segregation distortion towards the Col genotype.

We measured SFPs between the accessions Col and L*er* as significant differences in hybridization intensity of genomic DNA to oligonucleotide arrays. Depending on the significance threshold, we identified 20,450 SFPs (FDR = 0.05) corresponding to 7,920 genes (Table 1, Table S1), or 15,928 SFPs (FDR = 0.01) corresponding to 6,600 genes (Table 1, Table S2, and Figure S1). In general, 4–5% of all features on the array resulted in an SFP, that is, one SFP was identified for approximately one-third of all genes, occurring on average every 9 kb (Table S4). We observed, however, that the SFP-distribution along the chromosomes was not uniform. A significantly higher frequency of polymorphisms was found in peri-centromeric regions on each of the five chromosomes and in some other regions, for example, on the lower arms of Chromosomes 1 (Figure 1), 4, and 5 (Fisher's exact test, *p* value ≤ 0.05; Figure 1A and Figure S2). Genes with high polymorphism rates were often located in clusters such as the disease resistance gene clusters on Chromosomes 1, 3 (Resistance to Peronospora parasitica [RPP]-1 clusters), 4 (RPP-4, RPP-5, and RPP-2 clusters), and 5 (Resistance to Pseudomonas syringae cluster) [21]. Often those highly polymorphic gene clusters consist of multiple homologous genes belonging to one gene family, like leucine-rich repeat kinases, P450 proteins, and others. In addition, we observed 234 instances where all of the features corresponding to an entire L*er* transcript appeared not to hybridize, suggesting that the entire gene is not present in the L*er* accession (Table S3). This could be due to a deletion event in L*er* or an insertion event in the reference Col sequence.

**Table 1:**
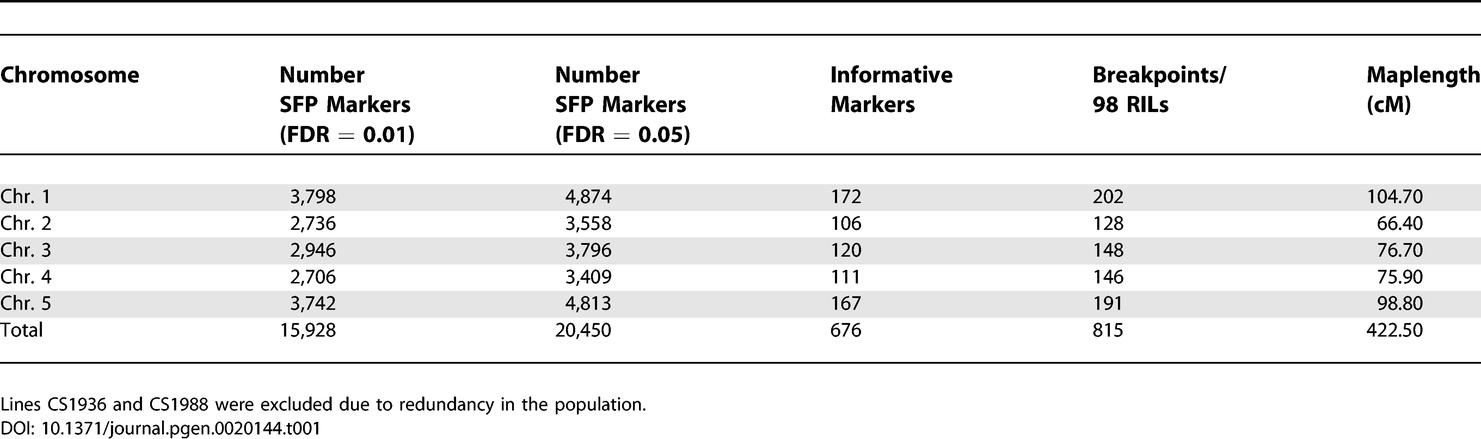
Genetic Linkage Map Characteristics and SFP Marker Summary for the 98 Col/L*er* RILs

### RIL-Population Genotyping

**Figure 2:**
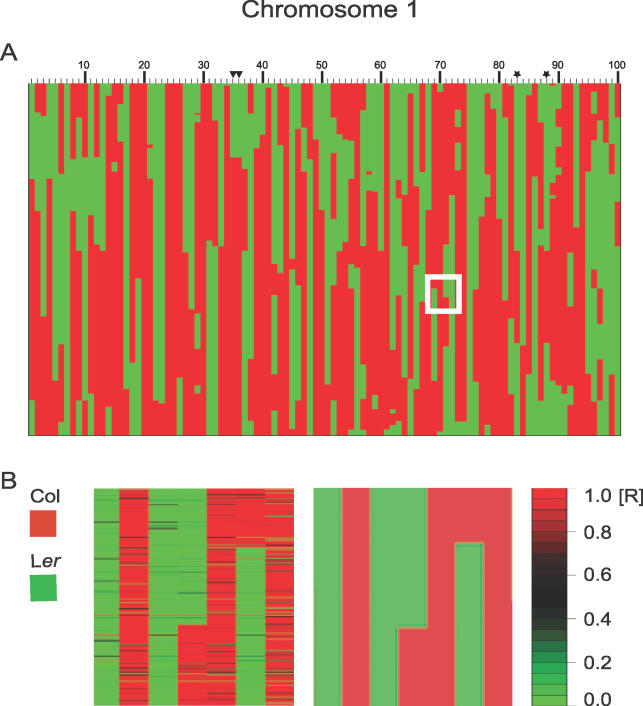
Graphical Genotype of Chromosome 1 for All Col/L*er* RILs (A) The 100 lines are arranged in numerical order according their CS number. Each column represents a single line from the Col/L*er* RIL population. The 3,798 SFP markers for Chromosome 1 are plotted vertically. Red areas indicate stretches of Col SFP alleles, green areas indicate L*er* genotype. The duplicated lines CS1935 and CS1936 are marked with black triangles and the duplicated lines CS1983 and CS1988 are marked with black stars. (B) A magnified view of the region marked with a white square in (A). Left: Genotyping results based on the computed ratio (color legend to the right; R = ratio) before SFP-calling and breakpoint determination. The complete results for all 100 RILs for Chromosome 1 are shown in Figure S3. Black lines indicate features where parental allele could not be determined. Red lines in stretches of green and green lines in stretches of red indicate possible genotyping errors, recombination events that were not deemed significant or gene conversion events. Right: Genotyping results after SFP-calling and breakpoint prediction with structural change analysis.

Next, we genotyped 100 RILs derived from a cross between Col and L*er* [13] by array hybridization (see Materials and Methods, Figure 2, and Figures S3–S7). In total, we identified 815 breakpoints at which crossovers had occurred (Table 1). Comparison of breakpoint locations revealed that lines CS1935 and CS1936 (recombination frequency [r] = 0.988), as well as lines CS1983 and CS1988 (r = 0.999) had nearly identical marker genotypes across the five chromosomes and therefore are likely redundant entries in the RIL mapping population (Figure 2A and Figures S3–S7). To rule out experimental error such as mislabeling of samples or files, we repeated sample preparation and array genotyping of each of the putative duplicate lines and corroborated the previous result. The redundant entries may have occurred while developing the original RIL set or during seed propagation at the stock center. Thus, this set contains 98 unique lines useful for linkage mapping.

Among the 98 RILs, we identified 31 lines with the greatest number of breakpoints over the five chromosomes. Therefore, these lines should be the most informative for mapping purposes (see Table 2 for a list of RILs and number of breakpoints for each line). Incidentally, 12 of these lines are different from those previously identified as the most informative RILs [22]. We identified 89 instances in 61 RILs for which no crossover had occurred along one, two, or three chromosomes. On average, 1.7 recombination events occurred per chromosome and 8.3 breakpoints in each line. Recombination breakpoints were flanked by two, non-overlapping SFPs, which can be nearby or distant. Therefore, breakpoint resolution is SFP-density dependent. Given the array-based genotype data, the average distance between two SFPs flanking a breakpoint was 33 kb. The smallest interval for which we could define a breakpoint was 5 bp and the largest interval was 385 kb.

**Table 2:**
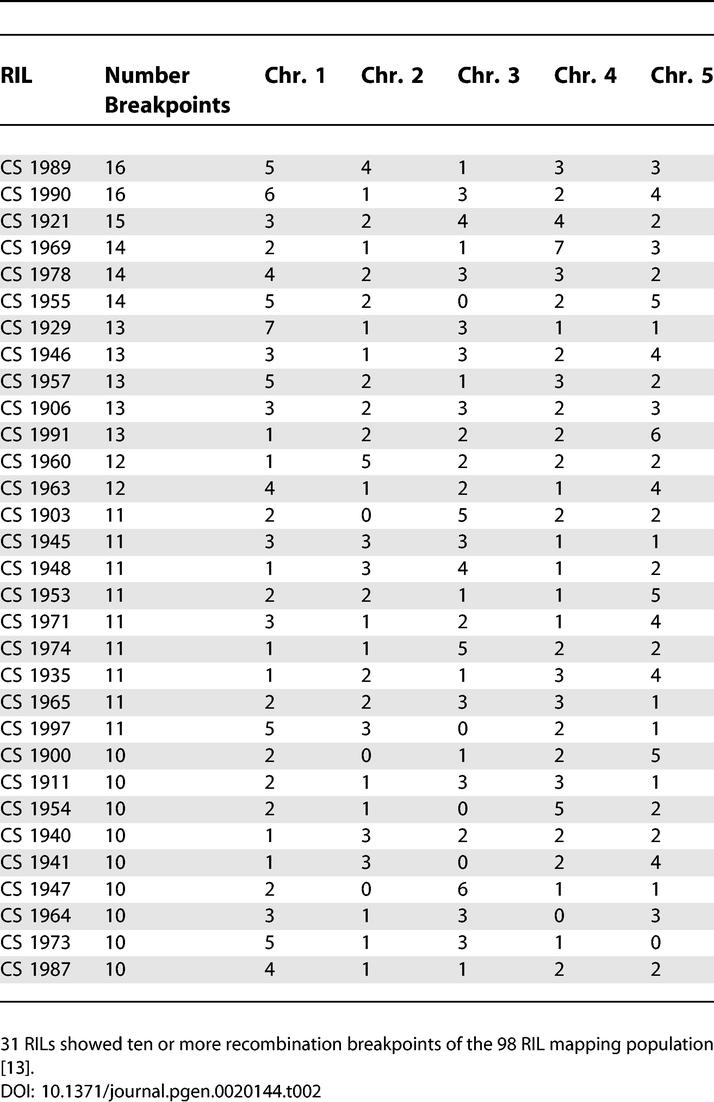
RILs with Highest Number of Recombination Breakpoints

Since the markers are all located in regions annotated as exons, it was possible to characterize the recombination events relative to genic regions. In 57 RILs we observed 105 instances (12% of all breakpoints) for which a breakpoint could be mapped within a single gene, ranging from one to four events per line. Therefore, intragenic recombination seems to occur frequently. Moreover, we observed seven instances where recombination occurred within the same gene in independent RILs. For example, a three-exon glycosyl hydrolase gene recombined in four different RILs (CS1939, CS1971, CS1978, and CS1990) and a 13-exon metallo-β-lactamase gene recombined in three different RILs (CS1920, CS1977, CS1994). Interestingly, in all instances the same pair of probes defined the recombination breakpoints. Although the exact location of the recombination event is not known, one explanation of this phenomenon is that RIL lines were not derived independently, i.e., one F_2_ plant gave rise to multiple F_3_ plants. Thus, a single recombination event would be preserved in more than one line. On the other hand, some genes may indeed be more prone to recombination than others. Further studies of newly generated F_2_ plants are needed to investigate this issue.

### Genetic and Physical Map

Since there were many more SFPs than breakpoints in the population, recombination was not observed between most of the markers. While markers that co-segregate without exception have different physical positions in the genome, they are genetically redundant. To create a minimal set of informative mapping markers, the RILs were divided into intervals of markers exhibiting the same genotype pattern across the 98 lines. An interval was defined as the smallest region flanked by two recombination breakpoints across 98 RILs with the exception of the terminal intervals, which were adjacent to telomeres. An SFP marker in the middle of each interval was selected as a proxy and declared an informative marker. In total, 676 informative markers were identified (Table 1, Figure S8). The physical location and probe sequence of each marker can be found in Table S5.

We calculated recombination frequencies based on breakpoint locations and used the genotype information of each informative marker as input into MAPMAKER/Exp software [23] to build a genetic linkage map (Figure S8, Table S7). Genotype information for each SFP marker in the Col/L*er* RILs can be found in Table S6. The genetic resolution of our map, calculated as the average genetic distance between informative SFP markers was 0.62 cM. The total map length was 422.5 cM (Table 1). On average, 43 genes were located between informative SFP markers, ranging from one to 492 genes per genetically defined interval (Table S8). With the exception of the terminal intervals, two SFP markers bordering each interval were identified. The average physical distance between interval SFP markers was 145 kb, ranging from 7 bp to 2.54 Mb. The average gene number between interval SFP markers was 37, ranging from one to 466 (Table S8). One hundred fifteen intervals were identified that harbor only a single gene (Table S9).

### Comparison to Existing Linkage Map Data

To assess the resolution of our SFP-based linkage map we compared it to a linkage map derived from publicly available marker data. Of 1,357 available markers total, 242 loci were amenable for linkage mapping after elimination of markers with more than 5% missing genotype information. The final map length was 397.1 cM, with an average map resolution of 1.67 cM and a maximum distance of 12.3 cM between two markers. The estimated number of breakpoints using this marker set was 1,025, compared to 815 breakpoints in the SFP-based marker map.

### Distribution of Recombination and Recombination Hot Spots

To assess the variability of recombination rate along the five *Arabidopsis* chromosomes, we compared the genetic distances between adjacent map intervals with their physical distances. The recombination rate was visualized by plotting the genetic distances between neighboring SFP markers versus the average physical distance for each map unit (Figures 1B, S9–S11). The recombination rate in the RIL population varied extensively within each of the five chromosomes, ranging from as high as 4 kb/cM (251 cM/Mb) to as low as ~3 Mb/cM (0.3 cM/Mb) (Table S10). Extreme peaks in recombination rate may indicate recombination hot spots whereas flat recombination rates indicate regions of suppressed recombination. To identify regions in the genome where significantly more crossovers occurred than expected by chance, we divided each chromosome into 50-kb windows and calculated a chi-square test-statistic for each window. After Bonferroni multiple-testing correction we found 37 windows with significantly elevated recombination rates (*p* ≤ 0.001) indicating the existence of recombination hot spots. Six to eight significant hot spots were found on each chromosome (Figure 1B and Figures S9–S11). A characteristic of recombination hot spot was that the gene or genes where crossovers occurred were mostly located in very small genetic intervals, consisting mostly of 1–2 genes, and that those genes almost always harbored one or more SFPs. Recombination also often occurred adjacent to the single-gene interval at the hot spot at the gene closest to the interval.

We calculated the average recombination rate for each chromosome from the slope of linear regression through the plot of the cM distance of each marker versus its physical position on the chromosome (Table S10 and Figures S9–S11). For all five *Arabidopsis* chromosomes we estimated the average genome-wide recombination rate to be 260 kb/cM (~4.0 cM/Mb) with the lowest recombination rates at the centromeres. Occasionally, other smaller regions along all five chromosomes exhibited depressed recombination rates as well. The largest section with the lowest recombination rate was in a region that comprises the heterochromatic knob [24] and the centromere on Chromosome 4. High recombination rates were observed at localized regions along the chromosomes and at the proximal telomeres of Chromosomes 2, 3, and 4 as well as the distal end of Chromosome 5. Clusters of peaks in recombination frequency also occurred either on one side (Chromosomes 3 and 5) or on both sides of the centromeres (Chromosomes 1, 2, and 4). Also, significantly more SFPs were identified in genomic regions where one or more recombination events occurred, suggesting a correlation between the occurrence of SFPs and recombination frequency (Fisher's exact test, Chromosomes 1–5, *p*-values ≤ 7.95 × 10^−22^).

### Segregation Distortion

Next, we tested the fit of the expected equal segregation ratio of Col and L*er* alleles in the RILs. Based on genotype distribution of informative SFP markers in 98 RILs, we found significant aberrations from the expected segregation ratio of 1:1 for all chromosomes except for Chromosome 3 for which no significant segregation distortion was apparent (Figure 1C, Figure S12). The long arm of Chromosome 1 showed significant segregation distortion in favor of Col alleles peaking at ~25 Mb at marker C1_136 (Col/Ler ratio: 2.4, Chi-square test, *p*-value = 4.08 × 10^−5^). The genetic interval at this marker with the most significant test statistic contains only a single gene, coding for a cytosolic glutamine synthetase. This gene (GLN1;2) contains an SFP as well.

## Discussion

Hybridizing DNA to a high-density oligonucleotide array can reliably detect copious DNA sequence polymorphisms. The number of SFPs detected with this approach is in part a function of the number of loci measured, corresponding to oligonucleotide array features. Approximately 4,000 SFPs were detected by hybridizing triplicate samples of Col and L*er* to the AtGenome1 Affymetrix array with features corresponding to ~7,000 genes [5]. In this study, we aimed to increase the number of highly significant SFPs between Col and L*er* by increasing the number of replicates and features on the array. We used an *Arabidopsis* whole genome array designed to detect ~26,000 distinct transcripts that represent ~10 Mb of sequence. Having identified ~16,000 (FDR = 0.01) and ~20,500 (FDR = 0.05) SFPs, this approach detected approximately 5-fold more SFPs than a preceding study. Approximately one-third of all genes harbor an SFP with one occurring every 0.5–0.6 kb of exon sequence. Using another method to detect SNPs between Col and L*er*, a polymorphism rate of one SNP per 1,000 kb was reported [20]. The greatest estimate for SNP rates in exons (one SNP every ~250–300 bp) was derived from sequencing 876 short fragments of 96 *Arabidopsis* accessions [25]. These results suggest that we detected ~50% of all possible SNPs with the exon array.

The distribution of SFPs along the chromosomes was not uniform with SFP density greatest near the centromeres, a phenomenon reported for amplified fragment length polymorphisms in *Arabidopsis,* potato, barley, soybean, and maize [18,26–29]. Peri-centromeric regions consist mainly of inactive heterochromatin that is primarily comprised of silenced retrotransposons and transposable elements [30]. Thus, a high degree of sequence divergence between those sequences is not surprising. Genes that exhibited significantly more SFPs than average were often closely clustered and are involved in disease resistance, defense, and signaling.

Linkage mapping accuracy is in part dependent of genotyping accuracy. Genotyping errors lead to ambiguous marker locations and paucity of markers diminishes trait-mapping accuracy. Both, ambiguous markers and reduced mapping accuracy complicate candidate gene discovery. Of the 1,357 markers scored in the Col/L*er* RIL population, only six have complete genotyping data and less than 20% were successfully genotyped in greater than 95% of the RILs. In our study the array features used to detect SFPs were derived from exon sequences and the exact genomic location of each marker is unambiguous. We array-genotyped all 100 individuals of the Col/L*er* RIL mapping population for all ~16,000 significant SFP markers. We were able to reliably predict recombination breakpoints with an established statistical method that is routinely employed to detect changes in econometrics and stock market trends [31]. It is a context-free method that detects significant changes in a dataset, regardless of how it was generated, even if it is noisy and lacking multiple replicated data points. Although some SFPs could not be assigned to either genotype or mis-scoring of genotypes may have occurred, these analyses are still likely to delineate the correct locations of crossovers. Residual heterozygosity, new mutations or gene conversion events may not have been detected with the number of replicates used in this study. Also double crossover events were not detected if an interval consisted only of a single feature. Nevertheless, recombination breakpoints were resolved with unprecedented resolution, in some instances defining a breakpoint between two adjacent features within a single gene or even exon. We found that intergenic recombination occurred frequently and that in several instances independently in multiple lines in the same gene. The true independence of these observations requires further examination in a newly generated F_2_ population. Based on the breakpoint locations, we defined genetic intervals in which no recombination occurred across all 98 RILs. Only ~4% of the ~16,000 SFP markers were required to identify a representative informative marker for each interval. Thus, the genetic resolution of our map is limited only by the number of recombination breakpoints in the Col/L*er* RILs population and not by the availability of markers. Even though resolution of this map is limited due to lack of introgression and population size, still 115 intervals harboring only a single were identified.

To estimate the increase in resolution, we compared our linkage map with a linkage map we derived from the existing public marker set. Using stringent selection criteria for marker inclusion from the public set, we expected to find fewer breakpoints than with the SFPs. Surprisingly, the number of breakpoints estimated using the public marker set exceeded the number of breakpoints using SFPs by more than 200. This discrepancy is either due to genotyping errors or missing genotype data in the existing public dataset, erroneously inflating the recombination breakpoint estimate. On the other hand it also possible that we may have overlooked small intervals created by double crossovers. An alignment of marker genotypes of the public dataset with our SFP-marker genotype data is difficult, since for many of the public markers (e.g. restriction fragment length polymorphisms) the exact physical position is not known. In cases where we genotyped one entire chromosome to be either Col or L*er* our data usually was in perfect agreement with the public dataset (e.g Chromosome 1 lines CS1994 and 1975, or Chromosome 4 lines CS1995 and CS1996). Also, there is complete agreement of the segregation distortion measured by Lister and Dean [13] with relatively few restriction fragment length polymorphism markers.

The total length of our SFP-based linkage map (422.5 cM) is considerably longer than that of the map derived from the previously published markers (397.1 cM), consistent with the observation that map length should increase with increasing marker density [11,32]. Although the resolution of the SFP-based linkage map is considerably greater than previous maps, the number of breakpoints in this mapping population delineates its genetic resolution. A larger population or a highly intercrossed population would have been desirable to increase map resolution.

One global measure of recombination rate is the relationship between physical distance and genetic distance. Our estimate of the genome-wide average recombination rate in the Col/L*er* RIL population was 285 kb/cM (3.5 cM/Mb). This is within the range of earlier reports estimating an average recombination rate of 221 kb/cM [33] and 208 kb/cM [34]. Compared to other organisms our estimated recombination rate is 7-fold greater than mouse (0.5 cM/Mb), [35], 5-fold greater than maize (0.7 cM/Mb) [36], 3-fold greater than humans (1.1 cM/Mb) [37], and 1.2-fold greater than *Drosophila* (2.9 cM/Mb) [38].

We found a high variability of recombination rates along the chromosomes ranging from as low as ~2–3 Mb/cM (~0.3–0.5 cM/Mb) at centromeric regions to peaks of ~4–10 kb/cM (100–250 kb/Mb) indicating recombination hot spots. The maximum local recombination rates were ~30–70-fold greater than the genome average. A similar phenomenon was reported in maize where recombination frequency at the *bronze* locus was 40–80-fold greater than the genome average [36]. Non-uniform distribution of recombination rate has been observed in a range of other organisms [39–41]. Since the L*er* genomic sequence is not completely known, variation in local recombination rates may also be due to large insertions/deletions in L*er*. With a few exceptions at the telomeres, our estimates of local recombination rates are generally in good agreement with the localized recombination patterns described in *Arabidopsis* [33,34]. Not surprisingly, the lowest recombination rate was observed at the centromeres at 10–12-fold below the genome-wide average. These findings are consistent with previous observations that *Arabidopsis* centromeres are recombinationally suppressed due to heavily methylated heterochromatin [33,42]. We also observed high recombination frequencies on one or both sides of the centromeres as well as elevated recombination activity for some telomeres, a phenomenon observed in *Arabidopsis* [34], mouse [43], and humans [44].

In mammals several recent studies suggest that haplotype blocks are largely defined by recombination hot spots and that those hot spots are clustered in small regions of 1–2 kb [41,45,46]. We also observed that local recombination occurs non-randomly in small localized clusters and sometimes independently between features within a single gene. Clustering of breakpoints in RILs can occur due to close crossover events in different generations in heterozygous regions that are not yet fixed [47].

Except for Chromosome 3, segregation distortion for several Mb stretches was prevalent. Genetic elements that distort Mendelian segregation to enhance their own transmission (so-called selfish genetic elements) are thought to be a potent evolutionary force [48]. Similar systems have been found in several crop species [49–52]. Segregation distortion has been observed before in *Arabidopsis* [8,13,18], but not at this resolution. One explanation how segregation distortion may have occurred is the possibility of selection over the course of inbreeding during RIL construction. On the other hand, genes in regions of segregation distortion may confer a selective advantage when in one or the other allelic state.

Another phenomenon that appears to be related to recombination is the positive correlation between recombination rate and nucleotide variability, also observed in this study and for *Drosophila*, *C. elegans,* humans, mice, and plants [39,53–62]. The simplest explanation for this phenomenon is that recombination and the associated repair of double strand breaks itself can be mutagenic [63]. While this explanation may hold true in humans [55] it is probably not the case in *Arabidopsis*, which is highly inbred [25]. More likely, the observed positive correlation of recombination rate and polymorphism in *Arabidopsis* can be explained by background selection eliminating unconditionally deleterious mutations [25,64,65] rather than genetic hitchhiking involving advantageous mutations sweeping through a population [66,67].

## Materials and Methods

### Plant material.

Seeds of the Columbia/Landsberg (Col/L*er*) RILs (eight generations of inbreeding) [13] and the parental lines were kindly provided by the *Arabidopsis* Biological Resource Center (ABRC) at the Ohio State University, Columbus, Ohio, United States. The accessions used in this study correspond to the first set of 100 RILs (CS1899), the parental lines were Columbia (CS933; Col-4, referred to as Col) and Landsberg *erecta* (CS20, referred to as L*er*). Eight plants for each RIL were grown in one pot under long-day conditions (16 h light, 8 h dark) and pooled for analysis.

### DNA isolation, labeling, and microarray hybridization.

Total genomic DNA was isolated from leaf tissue with the DNeasy Plant Mini Kit (Qiagen, Valencia, California, United States) according to the manufacturer's instructions. Four and six biological replicates of Col (CS933) and L*er* (CS20) were conducted, respectively. For RILs 1 (CS1900) to 57 (CS1957) a single replicate was available, with exception of line 6 (CS1906) which was triplicated. RILs 58 (CS1958) to 100 (CS4686) were duplicated, except line 73 (CS1973) was triplicated, and lines 83 (CS1983), 84 (CS1984), and 89 (CS1989) were quadruplicated. Probe intensity values for replicate microarrays of RILs were averaged for genotyping. DNA was labeled by random priming with biotin14-dCTP (Bioprime DNA labeling system, Invitrogen, Carlsbad, California, United States). Hybridization, washing, staining, and scanning was carried out using the standard Affymetrix Eukaryotic protocol. GeneChip Suite 4.0 (Affymetrix, Santa Clara, California, United States) was used for image acquisition.

### Array design and data analysis.

A custom *Arabidopsis* GeneChip^®^ array was designed by Torrey Mesa Research Institute and manufactured by Affymetrix, based on The Institute of Genomic Research (TIGR, Rockville, Maryland, United States) release of the *Arabidopsis* whole genome sequence, version 1.0, April, 2001. The 25-mer oligonucleotides on the GeneChip^®^ array were designed to correspond to annotated exon sequences and were selected as perfect match probes. No mismatch oligonucleotides were incorporated in the array. Each annotated gene was represented by ~15 probes (1–10 features/exon, depending on exon length), totaling 403,108 features (18-μm feature size) on the array. Analyses were performed using CEL files generated by Affymetrix GeneChip^®^ Suite 4.0 software. Arrays were background-corrected similar to the method described in the White Paper, “Statistical Algorithms Description Document” by Affymetrix (2002) (http://www.affymetrix.com/support/technical/whitepapers.affx). The probe intensity values were log-transformed and normalized to a mean of zero and standard deviation of one for each microarray. Prior to analysis, the custom array was re-annotated based on the TIGR genome release version 3.0, April 2003. After background correction and normalization 370,403 features, representing 26,136 genes were left for further analysis.

### Identification of SFPs and RIL genotyping.

To identify features that correspond to only a single genomic locus, the sequences of all features on the microarray were BLAST-searched against the TIGR *Arabidopsis* genome sequence, release 3.0. Features with more than one perfect match or a second match with an e-value less than 0.05 were discarded, as were probes that overlapped by more than 21 bp, leaving 331,031 unique single locus features. To identify SFPs with significantly higher hybridization signal in Col than in L*er* we calculated a FDR employing a permutation-based, non-symmetrical t-test statistic.

To assign a parental allele genotype for each SFP in each RIL, only SFPs (*n* = 12,987) with a 0.01 FDR along with the added criteria that Col hybridization was above background were considered. From the parental array replicates we obtained two t-distributions for each genotype (Col or L*er*). We defined Pr(Col) + Pr(L*er*) = 1. Using the data on the parental lines, we calculated Pr(y | Col) and Pr(y | L*er*). Using a 1:1 prior on Col:Ler, we calculated Pr(Col | y) = Pr(y | Col) / {Pr(y | Col) + Pr(y | L*er*)}. A ratio of 1.0 was defined as Col genotype, a value of 0.0 was defined as L*er* genotype, and for a value of 0.5, no genotype could be defined.

Using either three Col or three L*er* arrays as replicate reference sets we tested if we could reliably predict the known genotype of the remaining parental arrays (one Col array and three L*er* arrays). We treated the remaining parental arrays as a test set with unknown genotypes. We assessed the accuracy of our genotyping method by comparing the predicted genotypes in the test-set to the known genotypes in the reference set. We replicated this procedure for all possible permutations of arrays. The accuracy of the predictions was slightly less than perfect (Col = 97% and L*er* = 98%).

Locations of recombination breakpoints were estimated based on the ratio of hybridization intensities derived from RIL genotyping (Figure 2B). SFPs were ordered by physical position. A structural change analysis was performed using the ‘strucchange' module in R (‘efp' function) to estimate the location of breakpoints with confidence intervals [68]. The Bayesian Information Criterion (BIC) was used to aid in selecting the correct number of breakpoints. Essentially, the BIC is designed to choose a model that describes the data adequately while attempting to minimize parameters. The best model from among several competing models was selected. Starting with a maximum of 20 breakpoints per line a BIC value was calculated for each model under consideration, and the model with the smallest BIC value was chosen as the best model. Then the breakpoint locations were extracted from the model [69]. Finally, all markers between two breakpoints were assigned to be either Col or L*er* genotype, a process we termed ‘SFP-calling.'

### Genetic linkage mapping.

To construct the genetic linkage map, MAPMAKER/EXP version 3.0 [23] was modified to accommodate up to 500 markers per chromosome. Recombination frequencies (r) between informative markers were calculated from the fraction of recombinants (R) using the equation r = R/2(1–R) [11], and were converted to map distances in cM using the Kosambi mapping function [70]. A two-point analysis was performed using 676 markers with the default linkage criteria. Pairwise comparison of all loci with the ‘big lods' command (minimum LOD score 25.0, max distance 5.0 cM) showed that markers adjacent to each other by physical position were also linked with the highest LOD scores. For each chromosome, three-point analysis was performed. Map order was determined with the ‘order' command using full multipoint analysis. A permutation test of map orders was performed with the ‘ripple' command after each step to verify marker positions.

## Supporting Information

Figure S1 The T-Statistic Distribution Used to Determine Significant SFPs The values of the observed t-statistics (solid line) corresponding to 331,031 unique features are plotted against the expected “null” distribution (dotted line) obtained after 210 permutations. The dashed lines represent the 1% FDR threshold. Every feature with a value above the cutoff was recorded as a highly significant SFP. Features scoring below the cutoff had similar or greater hybridization intensities in L*er* and were therefore discarded.
(171 KB PDF)

Figure S2 Feature and SFP-Distribution on Chromosomes 2–5 The chromosomes were divided into 50-kb windows. Red triangles represent number of features per window. The number of SFPs per feature in each window was compared to the number of SFPs per feature in all windows using Fisher's exact test. A *p*-value was calculated and a Bonferroni multiple testing correction was applied to test for significance. Blue diamonds indicate windows with significantly higher SFP density (*p*-value ≤ 0.05). Position of centromeres are marked as black bars.
(2.0 MB PDF)

Figure S3 Graphical Genotype for Chromosome 1 before and after SFP-Calling and Breakpoint Prediction Each column represents a single accession from the Col/L*er* RIL population. The lines are arranged by CS number. SFP markers for each chromosome are plotted horizontally. Red indicates stretches of Col allele SFPs, green indicates the L*er* genotype. The duplicated lines CS1935 and CS1936 are marked with black triangles and the duplicated lines CS1983 and CS1988 are marked with black stars.
(A) Genotyping results based on the computed likelihood ratio before SFP-calling and breakpoint determination. Black lines indicate features where parental allele could not be determined. Red lines in stretches of green and green lines in stretches of red indicate possible genotyping errors, recombination events that were not deemed significant or gene conversion events.
(B) Genotyping results after SFP-calling and breakpoint prediction.
(1.9 MB PDF)

Figure S4 Graphical Genotype for Chromosome 2 before and after SFP-Calling and Breakpoint Prediction Each column represents a single accession from the Col/L*er* RIL population. The lines are arranged by CS number. SFP markers for each chromosome are plotted horizontally. Red indicates stretches of Col allele SFPs, green indicates the L*er* genotype. The duplicated lines CS1935 and CS1936 are marked with black triangles and the duplicated lines CS1983 and CS1988 are marked with black stars.
(A) Genotyping results based on the computed likelihood ratio before SFP-calling and breakpoint determination. Black lines indicate features where parental allele could not be determined. Red lines in stretches of green and green lines in stretches of red indicate possible genotyping errors, recombination events that were not deemed significant or gene conversion events.
(B) Genotyping results after SFP-calling and breakpoint prediction.
(1.0 MB PDF)

Figure S5 Graphical Genotype for Chromosome 3 before and after SFP-Calling and Breakpoint Prediction Each column represents a single accession from the Col/L*er* RIL population. The lines are arranged by CS number. SFP markers for each chromosome are plotted horizontally. Red indicates stretches of Col allele SFPs, green indicates the L*er* genotype. The duplicated lines CS1935 and CS1936 are marked with black triangles and the duplicated lines CS1983 and CS1988 are marked with black stars.
(A) Genotyping results based on the computed likelihood ratio before SFP-calling and breakpoint determination. Black lines indicate features where parental allele could not be determined. Red lines in stretches of green and green lines in stretches of red indicate possible genotyping errors, recombination events that were not deemed significant or gene conversion events.
(B) Genotyping results after SFP-calling and breakpoint prediction.
(1.2 MB PDF)

Figure S6 Graphical Genotype for Chromosome 4 before and after SFP-Calling and Breakpoint Prediction Each column represents a single accession from the Col/L*er* RIL population. The lines are arranged by CS number. SFP markers for each chromosome are plotted horizontally. Red indicates stretches of Col allele SFPs, green indicates the L*er* genotype. The duplicated lines CS1935 and CS1936 are marked with black triangles and the duplicated lines CS1983 and CS1988 are marked with black stars.
(A) Genotyping results based on the computed likelihood ratio before SFP-calling and breakpoint determination. Black lines indicate features where parental allele could not be determined. Red lines in stretches of green and green lines in stretches of red indicate possible genotyping errors, recombination events that were not deemed significant or gene conversion events.
(B) Genotyping results after SFP-calling and breakpoint prediction.
(3.0 MB PDF)

Figure S7 Graphical Genotype for Chromosome 5 before and after SFP-Calling and Breakpoint Prediction Each column represents a single accession from the Col/L*er* RIL population. The lines are arranged by CS number. SFP markers for each chromosome are plotted horizontally. Red indicates stretches of Col allele SFPs, green indicates the L*er* genotype. The duplicated lines CS1935 and CS1936 are marked with black triangles and the duplicated lines CS1983 and CS1988 are marked with black stars.
(A) Genotyping results based on the computed likelihood ratio before SFP-calling and breakpoint determination. Black lines indicate features where parental allele could not be determined. Red lines in stretches of green and green lines in stretches of red indicate possible genotyping errors, recombination events that were not deemed significant or gene conversion events.
(B) Genotyping results after SFP-calling and breakpoint prediction.
(3.2 MB PDF)

Figure S8 Graphical Representation of the Five *Arabidopsis* Chromosomes and the Physical Position of Each SFP Marker in the Genome Horizontal bars next to each chromosome represent informative SFP markers used to construct the genetic linkage map. The position of the first and last SFP marker on each chromosome and their respective genome position on each chromosome are noted. Centromeres are depicted as thick black lines. Total chromosome length is shown below each chromosome. Due to high marker density in some parts of the genome not all markers can be resolved in this view. Schematic chromosome view was adapted from NCBI (http://www.ncbi.nlm.nih.gov/mapview/map_search.cgi?taxid=3702).
(151 KB PDF)

Figure S9 Variation of Recombination Rates for Chromosome 1 (A) Recombination rates were calculated as the genetic distance (in cM/50 kb) between pairs of neighboring informative SFP markers and plotted versus the average physical distance between the same markers. The average genome-wide recombination rate is marked as a dotted line.
(B) Recombination variation visualized as a function of the cumulative genetic distance between adjacent informative SFP markers (in cM) versus the cumulative physical distance between the same markers. A regression line was fit to the data to determine the recombination rate for each chromosome. The location of the centromeres on each chromosome are marked with a black bar.
(308 KB PDF)

Figure S10 Variation of Recombination Rates for Chromosomes 2 and 3 (A) Recombination rates were calculated as the genetic distance (in cM/50 kb) between pairs of neighboring informative SFP markers and plotted versus the average physical distance between the same markers. The average genome-wide recombination rate is marked as a dotted line.
(B) Recombination variation visualized as a function of the cumulative genetic distance between adjacent informative SFP markers (in cM) versus the cumulative physical distance between the same markers. A regression line was fit to the data to determine the recombination rate for each chromosome. The location of the centromeres on each chromosome are marked with a black bar.
(1.5 MB PDF)

Figure S11 Variation of Recombination Rates for Chromosomes 4 and 5 (A) Recombination rates were calculated as the genetic distance (in cM/50 kb) between pairs of neighboring informative SFP markers and plotted versus the average physical distance between the same markers. The average genome-wide recombination rate is marked as a dotted line.
(B) Recombination variation visualized as a function of the cumulative genetic distance between adjacent informative SFP markers (in cM) versus the cumulative physical distance between the same markers. A regression line was fit to the data to determine the recombination rate for each chromosome. The location of the centromeres on each chromosome are marked with a black bar.
(1.8 MB PDF)

Figure S12 Segregation Distortion of SFP Markers for Chromosomes 2–5 Segregation ratios of genotypes for each informative SFP marker were calculated across 98 RILs and plotted along the chromosome. The vertical scale shows allele ratios. The expected equal distribution of Col and L*er* alles accross 98 lines should result in a ratio of 1 and is depicted as a dotted horizontal line. SFP markers with allele ratios above the line indicate segregation distortion towards the Col allele, SFP markers with allele ratios below the line indicate segregation distortion towards the L*er* allele. Empty diamonds represent SFP markers with no significant segregation distortion from the expected ratio of 1 between Col and L*er* genotypes. Filled triangles represent markers that show a significant (*p* ≤ 0.05, Fisher's exact test) segregation distortion towards the Col genotype.
(2.8 MB PDF)

Table S1 SFPs Identified between Col and L*er* Parental Lines at a 5% FDR after 210 Permutations (1.4 MB TXT)

Table S2 SFPs Identified between Col and L*er* Parental Lines at a 1% FDR after 210 Permutations (1.1 MB TXT)

Table S3 Putatively Deleted Genes in L*er* Compared to Col (228 KB DOC)

Table S4 Genome-Wide SFP Distribution Based on 50-kb Window Size (30 KB DOC)

Table S5 Informative SFP Markers Used for Genetic Mapping in 98 Lines of the Col/L*er* RIL Population [13] (1.3 MB DOC)

Table S6 List of Informative SFP Markers with Associated Genotypes for 98 Lines of the Col/L*er* RIL Population [13] (199 KB TXT)

Table S7 Genetic Linkage Map with Centimorgan Distances for the *Arabidopsis* Genome Based on 676 Informative SFP Markers for the Col/L*er* RIL Population [13]. (384 KB DOC)

Table S8 Genome-Wide Statistics on Interval Sizes and Number of Genes per Genetic Interval Based on Informative SFP Markers and on Interval SFP Markers (40 KB DOC)

Table S9 Genes Marking the Beginning and End of Each Recombination Interval for the 98 Lines of the Col/L*er* RIL Population [13] (1.5 MB DOC)

Table S10 Recombination Rates for Five *Arabidopsis* Chromosomes (35 KB DOC)

### Accession Numbers

The National Center for Biotechnology Information (NCBI) (http://www.ncbi.nlm.nih.gov) identifiers for the genes and gene products discussed in this study include glycosyl hydrolase (At5g09700, NP_196532), metallo-β-lactamase (At2g01730, NP_178282), and cytosolic glutamine synthetase (At1g66200, NP_001031240). Microarray data (.CEL files) have been deposited with ArrayExpress (http://www.ebi.ac.uk/arrayexpress/) under accession number E-TABM-135. The accession number for the array design (.CDF file) is A-AFFY-73.

## Abbreviations

BIC: Bayesian Information Criterion
Col: Columbia
FDR: false discovery rate
L*er*: Landsberg *erecta*
*r*: recombination frequency
R: fraction of recombinants
RIL: recombinant inbred line
RPP: Resistance to Peronospora parasitica
SFPs: single feature polymorphism
SNP: single nucleotide polymorphism
