# Supplementary material for: A High-Resolution Map of Arabidopsis Recombinant Inbred Lines by Whole-Genome Exon Array Hybridization
Source: PLoS Genet. 2006 Sep 15;2(9):e144. doi: 10.1371/journal.pgen.0020144 (PMC1564425; doi:10.1371/journal.pgen.0020144)
Supplement: Supplementary file 13 [file pgen.0020144.st003.doc]

| Functional category | Gene ID | annotation |
| --- | --- | --- |
| Expressed & Hypothetical | At1g24480 | hypothetical protein similar to EST gb\|AA394324 |
| Expressed & Hypothetical | At1g31370 | hypothetical protein |
| Expressed & Hypothetical | At1g31960 | hypothetical protein |
| Expressed & Hypothetical | At1g32030 | hypothetical protein |
| Expressed & Hypothetical | At1g41930 | hypothetical protein |
| Expressed & Hypothetical | At1g42400 | hypothetical protein common family includes At2g10370, At5g36050, At1g35090, At1g44860, At4g19300, At1g42400, At3g43040, At3g42500 |
| Expressed & Hypothetical | At1g48290 | hypothetical protein |
| Expressed & Hypothetical | At1g63620 | pseudogene, hypothetical protein GB:AAD26889 |
| Expressed & Hypothetical | At1g63890 | unknown protein |
| Expressed & Hypothetical | At1g76960 | expressed protein |
| Expressed & Hypothetical | At1g80970 | hypothetical protein |
| Expressed & Hypothetical | At2g05090 | hypothetical protein common family includes At5g37080, At5g37170, At2g05090 |
| Expressed & Hypothetical | At2g06820 | hypothetical protein low similarity to zinc finger protein (Arabidopsis thaliana) GI:976277 |
| Expressed & Hypothetical | At2g07390 | hypothetical protein |
| Expressed & Hypothetical | At2g11010 | hypothetical protein |
| Expressed & Hypothetical | At2g13660 | hypothetical protein |
| Expressed & Hypothetical | At2g15170 | expressed protein similar to hypothetical protein GB:AAD00377 |
| Expressed & Hypothetical | At2g15180 | hypothetical protein contains CCHC zinc-finger domain |
| Expressed & Hypothetical | At2g15800 | hypothetical protein |
| Expressed & Hypothetical | At2g17920 | hypothetical protein similar to zinc finger protein (Arabidopsis thaliana) GI:976277 |
| Expressed & Hypothetical | At2g18610 | hypothetical protein |
| Expressed & Hypothetical | At2g22340 | hypothetical protein |
| Expressed & Hypothetical | At2g27870 | hypothetical protein |
| Expressed & Hypothetical | At2g29160 | pseudogene, tropinone reductase non-consensus GA donor splice site at exon 2 |
| Expressed & Hypothetical | At2g29830 | hypothetical protein |
| Expressed & Hypothetical | At2g29880 | hypothetical protein |
| Expressed & Hypothetical | At2g34110 | hypothetical protein |
| Expressed & Hypothetical | At3g11350 | hypothetical protein |
| Expressed & Hypothetical | At3g16750 | hypothetical protein |
| Expressed & Hypothetical | At3g24630 | hypothetical protein |
| Expressed & Hypothetical | At3g28170 | hypothetical protein |
| Expressed & Hypothetical | At3g29080 | hypothetical protein |
| Expressed & Hypothetical | At3g30320 | hypothetical protein |
| Expressed & Hypothetical | At3g30655 | hypothetical protein |
| Expressed & Hypothetical | At3g42190 | hypothetical protein several hypothetical proteins - Arabidopsis thaliana |
| Expressed & Hypothetical | At3g42290 | hypothetical protein predicted proteins, Arabidopsis thaliana |
| Expressed & Hypothetical | At3g42310 | hypothetical protein |
| Expressed & Hypothetical | At3g42340 | hypothetical protein various predicted Helicases, Arabidopsis thaliana |
| Expressed & Hypothetical | At3g42370 | putative protein |
| Expressed & Hypothetical | At3g42390 | hypothetical protein |
| Expressed & Hypothetical | At3g42440 | hypothetical protein several hypothetical proteins - Arabidopsis thaliana |
| Expressed & Hypothetical | At3g42550 | hypothetical protein several putative proteins - Arabidopsis thaliana |
| Expressed & Hypothetical | At3g42910 | hypothetical protein predicted proteins, Arabidopsis thaliana |
| Expressed & Hypothetical | At3g42920 | hypothetical protein predicted proteins, Arabidopsis thaliana |
| Expressed & Hypothetical | At3g42940 | expressed protein |
| Expressed & Hypothetical | At3g43170 | hypothetical protein predicted proteins, Arabidopsis thaliana |
| Expressed & Hypothetical | At3g43330 | hypothetical protein putative proteins - Arabidopsis thaliana |
| Expressed & Hypothetical | At3g43760 | hypothetical protein predicted proteins, Arabidopsis thaliana |
| Expressed & Hypothetical | At3g43770 | hypothetical protein predicted proteins, Arabidopsis thaliana |
| Expressed & Hypothetical | At3g44430 | expressed protein |
| Expressed & Hypothetical | At3g45350 | hypothetical protein common family similar to At4g09400, At2g14330 |
| Expressed & Hypothetical | At3g45510 | hypothetical protein hypothetical protein At2g26130 - Arabidopsis thaliana, EMBL:AAC31224 |
| Expressed & Hypothetical | At3g47920 | hypothetical protein similar to zinc finger protein (Arabidopsis thaliana) GI:976277 |
| Expressed & Hypothetical | At3g50480 | expressed protein |
| Expressed & Hypothetical | At4g03480 | hypothetical protein similar to A. thaliana hypothetical protein F21B7.8, GenBank accession number AC002560 |
| Expressed & Hypothetical | At4g07800 | hypothetical protein |
| Expressed & Hypothetical | At4g07920 | hypothetical protein hypothetical protein T24H24.5 - Arabidopsis thaliana,Pir2:T01467 |
| Expressed & Hypothetical | At4g07930 | hypothetical protein hypothetical protein T24H24.5 - Arabidopsis thaliana,PIR2:T01467 |
| Expressed & Hypothetical | At4g09700 | hypothetical protein similar to zinc finger protein (Arabidopsis thaliana) GI:976277 |
| Expressed & Hypothetical | At4g15600 | hypothetical protein |
| Expressed & Hypothetical | At4g20220 | hypothetical protein |
| Expressed & Hypothetical | At5g01140 | hypothetical protein predicted protein, Arabidopsis thaliana |
| Expressed & Hypothetical | At5g20450 | hypothetical protein unconventional myosin heavy chain, Zea mays, PIR:A59310 |
| Expressed & Hypothetical | At5g20770 | hypothetical protein |
| Expressed & Hypothetical | At5g22520 | expressed protein |
| Expressed & Hypothetical | At5g25010 | hypothetical protein |
| Expressed & Hypothetical | At5g27890 | expressed protein predicted proteins, Arabidopsis thaliana |
| Expressed & Hypothetical | At5g39770 | hypothetical protein predicted protein, Arabidopsis thaliana |
| Expressed & Hypothetical | At5g42460 | expressed protein strong similarity to unknown protein (pir\|\|T00841) |
| Expressed & Hypothetical | At5g45450 | hypothetical protein similar to unknown protein (gb\|AAD49762.1) |
| Expressed & Hypothetical | At5g45460 | expressed protein similar to unknown protein (pir\|\|T04430) |
| Expressed & Hypothetical | At5g56470 | hypothetical protein strong similarity to unknown protein (pir\|\|T02676) |
| Other | At1g23630 | OBP32pep protein -related identical to putative OBP32pep protein GB:AAC98019 GI:4056446 from (Arabidopsis thaliana) |
| Other | At1g33530 | F-box protein family contains F-box domain Pfam:PF00646 |
| Other | At1g52990 | thioredoxin family similar to SP\|P48384 Thioredoxin M-type, chloroplast precursor (TRX-M) {Pisum sativum}; contains Pfam profile PF00085: Thioredoxin |
| Other | At1g53950 | ubiquitin family contains INTERPRO:IPR000626 ubiquitin domain |
| Other | At1g53980 | polyubiquitin -related similar to polyubiquitin GI:166336 from (Aglaothamnion neglectum) |
| Other | At1g65350 | polyubiquitin, putative similar to polyubiquitin GI:248337 from (Zea mays) |
| Other | At1g73530 | RNA recognition motif (RRM) - containing protein low similarity to SP\|Q03251 Glycine-rich RNA-binding protein 8 (CCR1 protein) {Arabidopsis thaliana}; contains InterPro entry IPR000504: RNA-binding region RNP-1 (RNA recognition motif) (RRM) |
| Other | At2g15420 | myosin heavy chain-related |
| Other | At2g25970 | KH domain protein |
| Other | At3g43550 | lipase, putative similar to family II lipase EXL3 (GI:15054386), EXL1 (GI:15054382), EXL2 (GI:15054384) (Arabidopsis thaliana); contains Pfam profile PF00657: Lipase/Acylhydrolase with GDSL-like motif |
| Other | At3g43570 | lipase, putative similar to family II lipases EXL3 GI:15054386, EXL1 GI:15054382, EXL2 GI:15054384 from (Arabidopsis thaliana); contains Pfam profile PF00657: GDSL-like Lipase/Acylhydrolase |
| Other | At3g44010 | 40S ribosomal protein S29 (RPS29B) ribosomal protein S29, rat, PIR:S30298 |
| Other | At4g04390 | Ulp1 protease family contains Pfam profile PF02902: Ulp1 protease family, C-terminal catalytic domain |
| Other | At4g05490 | F-box protein family, AtFBL22 contains similarity to N7 protein GI:3273101 from (Medicago truncatula) |
| Other | At4g07580 | Ulp1 protease family contains Pfam profile PF02902: Ulp1 protease family, C-terminal catalytic domain |
| Other | At4g12500 | protease inhibitor/seed storage/lipid transfer protein (LTP) family similar to pEARLI 1 (Accession No. L43080): an Arabidopsis member of a conserved gene family (PGF95-099), Plant Physiol. 109 (4), 1497 (1995); contains Pfam protease inhibitor/seed storage/LTP family domain PF00234 |
| Other | At4g20210 | terpene synthase/cyclase family (+)-delta-cadinene synthase isozyme XC14, Gossypiumarboreum, PIR2:S68366 |
| Other | At4g24980 | nodulin MtN21 family protein similar to MtN21 (Medicago truncatula) GI:2598575 |
| Other | At5g05900 | glucuronosyl transferase-related protein |
| Other | At5g18880 | SAE1-S9-protein - like SAE1-S9-protein, Brassica rapa, EMBL:AB012866 |
| Other | At5g37610 | porin -related protein porin, Prunus armeniaca, EMBL:AF139498 |
| Other | At5g50140 | ankyrin repeat protein family contains ankyrin repeat domains, Pfam:PF00023 |
| Signal Transd. & Defense | At1g15890 | disease resistance protein (CC-NBS-LRR class), putative domain signature CC-NBS-LRR exists, suggestive of a disease resistance protein. |
| Signal Transd. & Defense | At1g60320 | disease resistance protein (TIR class), putative domain signature TIR exists, suggestive of a disease resistance protein. |
| Signal Transd. & Defense | At1g62090 | hypothetical protein similar to putative receptor-like protein kinase GI:3461841 from (Arabidopsis thaliana) |
| Signal Transd. & Defense | At1g62100 | receptor-related protein kinase similar to receptor-like protein kinase GI:9758833 from (Arabidopsis thaliana) |
| Signal Transd. & Defense | At1g63870 | disease resistance protein (TIR-NBS-LRR class), putative domain signature TIR-NBS-LRR exists, suggestive of a disease resistance protein. |
| Signal Transd. & Defense | At1g63880 | disease resistance protein (TIR-NBS-LRR class), putative domain signature TIR-NBS-LRR exists, suggestive of a disease resistance protein. |
| Signal Transd. & Defense | At1g65190 | expressed protein contains similarity to serine threonine kinase GI:166813 from (Arabidopsis thaliana) |
| Signal Transd. & Defense | At1g66980 | kinase -related similar to leaf rust resistance kinase Lr10 GI:1680685 from (Triticum aestivum) |
| Signal Transd. & Defense | At1g69550 | disease resistance protein (TIR-NBS class), putative domain signature TIR-NBS exists, suggestive of a disease resistance protein. |
| Signal Transd. & Defense | At2g26820 | avrRpt2-induced protein 1 -related |
| Signal Transd. & Defense | At3g24900 | disease resistance protein family (LRR) contains leucine rich-repeat domains Pfam:PF00560, INTERPRO:IPR001611; similar to Cf-2.2 (Lycopersicon pimpinellifolium) gi\|1184077\|gb\|AAC15780 |
| Signal Transd. & Defense | At3g46370 | leucine rich repeat protein kinase, putative similar to light repressible receptor protein kinase (Arabidopsis thalian) gi\|1321686\|emb\|CAA66376; contains leucine rich repeat (LRR) domains, Pfam:PF00560; contains protein kinase domain, Pfam:PF00069 |
| Signal Transd. & Defense | At4g07670 | peptidase -related |
| Signal Transd. & Defense | At4g09360 | disease resistance protein (NBS-LRR class), putative domain signature NBS-LRR exists, suggestive of a disease resistance protein. |
| Signal Transd. & Defense | At4g09420 | disease resistance protein (TIR-NBS class), putative domain signature TIR-NBS exists, suggestive of a disease resistance protein. |
| Signal Transd. & Defense | At4g09430 | disease resistance protein (TIR-NBS-LRR class), putative domain signature TIR-NBS-LRR exists, suggestive of a disease resistance protein. |
| Signal Transd. & Defense | At4g16930 | disease resistance protein (TIR-NBS class), putative domain signature TIR-NBS exists, suggestive of a disease resistance protein. |
| Signal Transd. & Defense | At4g16960 | disease resistance protein (TIR-NBS-LRR class), putative domain signature TIR-NBS-LRR exists, suggestive of a disease resistance protein. |
| Signal Transd. & Defense | At4g19760 | glycosyl hydrolase family 18 similar to chitinase, class V GI:505267 from (Nicotiana tabacum) |
| Signal Transd. & Defense | At4g23220 | serine/threonine kinase - like protein serine/threonine kinase, Brassica oleracea |
| Signal Transd. & Defense | At4g27190 | disease resistance protein (NBS-LRR class), putative domain signature NBS-LRR exists, suggestive of a disease resistance protein. |
| Signal Transd. & Defense | At4g27220 | disease resistance protein (NBS-LRR class), putative domain signature NBS-LRR exists, suggestive of a disease resistance protein. |
| Signal Transd. & Defense | At5g10610 | cytochrome P450 family similar to Cytochrome P450 91A1 (SP:Q9FG65) (Arabidopsis thaliana); similar to cytochrome P450, Helianthus tuberosus, EMBL:HTCYP81L |
| Signal Transd. & Defense | At5g17890 | disease resistance protein (TIR-NBS-LRR class), putative domain signature TIR-NBS-LRR exists, suggestive of a disease resistance protein. |
| Signal Transd. & Defense | At5g18350 | disease resistance protein (TIR-NBS-LRR class), putative domain signature TIR-NBS-LRR exists, suggestive of a disease resistance protein. |
| Signal Transd. & Defense | At5g43740 | disease resistance protein (CC-NBS-LRR class), putative domain signature CC-NBS-LRR exists, suggestive of a disease resistance protein. |
| Signal Transd. & Defense | At5g45070 | disease resistance protein (TIR class), putative domain signature TIR exists, suggestive of a disease resistance protein. |
| Signal Transd. & Defense | At5g45090 | disease resistance protein (TIR class), putative domain signature TIR exists, suggestive of a disease resistance protein; low similarity to PP2 lectin polypeptide (Cucurbita maxima) GI:410437 |
| Signal Transd. & Defense | At5g45220 | disease resistance protein (TIR class), putative domain signature TIR exists, suggestive of a disease resistance protein. |
| Signal Transd. & Defense | At5g45230 | disease resistance protein (TIR-NBS-LRR class), putative domain signature TIR-NBS-LRR exists, suggestive of a disease resistance protein. |
| Signal Transd. & Defense | At5g46510 | disease resistance protein (TIR-NBS-LRR class), putative domain signature TIR-NBS-LRR exists, suggestive of a disease resistance protein. |
| Signal Transd. & Defense | At5g48620 | disease resistance protein (CC-NBS-LRR class), putative domain signature CC-NBS-LRR exists, suggestive of a disease resistance protein. |
| Signal Transd. & Defense | At5g59650 | leucine rich repeat protein kinase, putative contains leucine rich repeat (LRR) domains, Pfam:PF00560; contains protein kinase domain, Pfam:PF00069 |
| Signal Transd. & Defense | At5g59660 | leucine rich repeat protein kinase, putative similar to light repressible receptor protein kinase (Arabidopsis thaliana) gi\|1321686\|emb\|CAA66376; contains leucine rich repeat (LRR) domains, Pfam:PF00560; contains protein kinase domain, Pfam:PF00069 |
| Transcription | At1g30500 | transcription factor -related similar to transcription factor GB:CAA74050 GI:2398525 from (Arabidopsis thaliana) |
| Transcription | At1g63040 | DRE binding protein similar to GI:3738224 from (Arabidopsis thaliana) (Plant Cell 10 (8), 1391-1406 (1998)) |
| Transcription | At2g17590 | CHP-rich zinc finger protein, putative |
| Transcription | At3g11370 | CHP-rich zinc finger protein, putative similar to unknown protein GB:AAD32917 (Arabidopsis thaliana) |
| Transcription | At3g24680 | zinc finger protein identical to zinc-finger protein GI:976277 from (Arabidopsis thaliana) |
| Transcription | At3g26250 | CHP-rich zinc finger protein, putative |
| Transcription | At3g31430 | hypothetical protein low similarity to zinc finger protein (Arabidopsis thaliana) GI:976277 |
| Transcription | At3g45530 | CHP-rich zinc finger protein, putative several hypothetical proteins - Arabidopsis thaliana |
| Transcription | At3g47680 | myb family protein |
| Transcription | At4g01920 | CHP-rich zinc finger protein, putative similar to A. thaliana CHP-rich proteins |
| Transcription | At4g01930 | CHP-rich zinc finger protein, putative similar to A. thalian CHP-rich proteins |
| Transcription | At4g07950 | DNA-directed RNA polymerase subunit -related similar to A. fulgidus DNA-directed RNA polymerase subunit M, GenBank accession number AE001019 |
| Transcription | At5g02330 | CHP-rich zinc finger protein, putative various predicted proteins, Arabidopsis thaliana |
| Transcription | At5g54040 | CHP-rich zinc finger protein, putative |
| Transposons & pseudogenes | At1g10000 | reverse transcriptase -related similar to GB:AAD29058 |
| Transposons & pseudogenes | At1g22560 | pseudogene, similar to reverse transcriptase (LINE-element) (Mus musculus) (GB:BAA20419) |
| Transposons & pseudogenes | At1g23990 | reverse transcriptase (RNA-dependent DNA polymerase) family protein low similarity to reverse transcriptase (Arabidopsis thaliana) GI:976278; contains Pfam profile PF00078: Reverse transcriptase (RNA-dependent DNA polymerase) |
| Transposons & pseudogenes | At1g26990 | pseudogene, similar to open reading frame 1 (Ty1_Copia-element) (Brassica oleracea) (GB:CAA72989) similar to polyprotein GB:AAC02664 GI:2865424 from (Arabidopsis thaliana) |
| Transposons & pseudogenes | At1g31030 | reverse transcriptase (RNA-dependent DNA polymerase) family protein similar to reverse transcriptase (Arabidopsis thaliana) GI:976278; contains Pfam profiles PF00078: Reverse transcriptase (RNA-dependent DNA polymerase) |
| Transposons & pseudogenes | At1g33510 | pseudogene, reverse transcriptase |
| Transposons & pseudogenes | At1g34080 | hypothetical protein similar to putative retroelement gag/pol polyprotein GI:4557066 from (Arabidopsis thaliana) |
| Transposons & pseudogenes | At1g34090 | polyprotein -related similar to polyprotein GI:4996361 from (Arabidopsis thaliana) |
| Transposons & pseudogenes | At1g34240 | hypothetical protein contains similarity to Ac-like transposase GI:5306256 from (Arabidopsis thaliana) |
| Transposons & pseudogenes | At1g35130 | pseudogene, similar to Athila ORF 1 (Arabidopsis thaliana) (GB:CAA57397) similar to putative Athila retroelement ORF1 protein GB:AAD19762 GI:4388724 from (Arabidopsis thaliana) |
| Transposons & pseudogenes | At1g42110 | transposase -related similar to Ac-like transposase GI:5306256 from (Arabidopsis thaliana) |
| Transposons & pseudogenes | At1g42130 | pseudogene, similar to putative transposase protein (CACTA-element) transposon="Shooter" (Zea mays) (GB:AAD55677) similar to En/Spm-like transposon protein GI:4115354 from (Arabidopsis thaliana) |
| Transposons & pseudogenes | At1g42160 | pseudogene, putative Athila retroelement protein |
| Transposons & pseudogenes | At1g42170 | pseudogene, similar to polyprotein (Gypsy_Ty3-element) (Ananas comosus) (GB:CAA73042) similar to polyprotein GI:2995405 from (Ananas comosus |
| Transposons & pseudogenes | At1g42180 | pseudogene, putative gag-pol polyprotein |
| Transposons & pseudogenes | At1g42190 | hypothetical protein similar to putative transposon protein GI:7267139 from (Arabidopsis thaliana) |
| Transposons & pseudogenes | At1g42340 | pseudogene (MIPS) |
| Transposons & pseudogenes | At1g43250 | pseudogene, similar to ORF2 (Mus musculus) (GB:AAA39398) |
| Transposons & pseudogenes | At1g43280 | pseudogene, similar to mudrA of transposon="MuDR" (MuDr-element) (Zea mays) (GB:AAA21566) |
| Transposons & pseudogenes | At1g47430 | pseudogene, putative reverse transcriptase |
| Transposons & pseudogenes | At1g52210 | pseudogene, similar to retrofit (TY1_Copia-element) (Oryza longistaminata) (GB:AAB82754) |
| Transposons & pseudogenes | At1g62550 | pseudogene, putative non-LTR retroelement reverse transcriptase similar to non-LTR retroelement reverse transcriptase-like protein GI:9758853 from (Arabidopsis thaliana) |
| Transposons & pseudogenes | At1g65750 | pseudogene, similar to reverse transcriptase (LINE-element) (Mus musculus) (GB:AAA67727) |
| Transposons & pseudogenes | At1g67240 | mutator-related transposase similar to mutator-like transposase GI:5306250 from (Arabidopsis thaliana) |
| Transposons & pseudogenes | At2g03960 | pseudogene, similar to hypothetical protein GB:AAD31338 |
| Transposons & pseudogenes | At2g05200 | reverse transcriptase (RNA-dependent DNA polymerase) family protein similar to reverse transcriptase (Arabidopsis thaliana) GI:976278; contains Pfam profile PF00078: Reverse transcriptase (RNA-dependent DNA polymerase) |
| Transposons & pseudogenes | At2g05390 | retroelement pol polyprotein -related |
| Transposons & pseudogenes | At2g06540 | pseudogene, similar to L1 repeat, Tf subfamily, member 30 (LINE-element) (Mus musculus) (GB:NP_038605) |
| Transposons & pseudogenes | At2g06560 | pseudogene, similar to ORF2 (LINE-element) (Rattus norvegicus) (GB:AAB41224) |
| Transposons & pseudogenes | At2g06660 | CENP-B/ARS binding protein -related protein also related to Drosophila pogo transposon proein |
| Transposons & pseudogenes | At2g06710 | pseudogene, similar to hypothetical protein GB:AAD14512 |
| Transposons & pseudogenes | At2g06950 | retroelement pol polyprotein -related |
| Transposons & pseudogenes | At2g07580 | pseudogene, similar to hypothetical protein GB:AF058825 |
| Transposons & pseudogenes | At2g10100 | pseudogene, similar to Athila ORF 1 (Arabidopsis thaliana) (GB:CAA57397) |
| Transposons & pseudogenes | At2g10620 | Athila retroelement ORF1 protein -related |
| Transposons & pseudogenes | At2g11100 | pseudogene, similar to Athila ORF 1 (Arabidopsis thaliana) (GB:CAA57397) |
| Transposons & pseudogenes | At2g11140 | retroelement pol polyprotein -related |
| Transposons & pseudogenes | At2g11190 | retroelement pol polyprotein -related |
| Transposons & pseudogenes | At2g11240 | reverse transcriptase (RNA-dependent DNA polymerase) family protein similar to reverse transcriptase (Arabidopsis thaliana) GI:976278; contains Pfam profiles PF00078: Reverse transcriptase (RNA-dependent DNA polymerase), PF03372: Endonuclease/Exonuclease/phosphatase family |
| Transposons & pseudogenes | At2g11290 | pseudogene, similar to polyprotein (Gypsy_Ty3-element) (Oryza sativa subsp. indica) (GB:AAD27547) |
| Transposons & pseudogenes | At2g11580 | hypothetical protein very low similarity to transposase DOPD (Zea mays) GI:10441445 |
| Transposons & pseudogenes | At2g12000 | pseudogene, fragment of a MADS-box protein |
| Transposons & pseudogenes | At2g12450 | pseudogene, similar to prpol (gypsy_Ty3-element) (Zea mays) (GB:AAD11615) |
| Transposons & pseudogenes | At2g12880 | CCHC-type zinc finger protein -related contains a CCHC type zinc-finger domain (QDOC50158); related to retroviral gag proteins |
| Transposons & pseudogenes | At2g13940 | retroelement pol polyprotein -related contains a Zn-finger CCHC type domain (prosite:QDOC50158) and a DDE integrase signature motif |
| Transposons & pseudogenes | At2g13970 | Mutator transposase-related similar to MURAZC (Zea mays) gi\|1857256\|gb\|AAB48408 |
| Transposons & pseudogenes | At2g14630 | hypothetical protein contains Pfam profile PF03004: Plant transposase (Ptta/En/Spm family) |
| Transposons & pseudogenes | At2g14640 | retroelement pol polyprotein -related |
| Transposons & pseudogenes | At2g15410 | retrotransposon gag protein family similar to GAG-POL precursor (Oryza sativa (japonica cultivar-group)) GI:5902445; contains Pfam profiles PF00078: Reverse transcriptase (RNA-dependent DNA polymerase), PF00665: Integrase core domain, PF03732: Retrotransposon gag protein |
| Transposons & pseudogenes | At2g15540 | reverse transcriptase (RNA-dependent DNA polymerase), putative similar to reverse transcriptase (Arabidopsis thaliana) GI:976278; contains Pfam profiles PF03372: Endonuclease/Exonuclease/phosphatase family, PF00078: Reverse transcriptase (RNA-dependent DNA polymerase) |
| Transposons & pseudogenes | At2g15810 | Mutator-related transposase similar to MURA transposase of maize Mutator transposon |
| Transposons & pseudogenes | At2g15940 | Ac-related transposase related to Ac/Ds transposon family of maize |
| Transposons & pseudogenes | At2g16110 | pseudogene, putative non-LTR retrolelement reverse transcriptase |
| Transposons & pseudogenes | At2g16540 | pseudogene, reverse transcriptase |
| Transposons & pseudogenes | At2g17610 | reverse transcriptase (RNA-dependent DNA polymerase) family protein similar to reverse transcriptase (Arabidopsis thaliana) GI:976278; contains Pfam profile PF00078: Reverse transcriptase (RNA-dependent DNA polymerase) |
| Transposons & pseudogenes | At2g18580 | Ac-related transposase related to Ac/Ds transposon family of maize |
| Transposons & pseudogenes | At2g19840 | copia-related retroelement pol polyprotein |
| Transposons & pseudogenes | At2g22210 | pseudogene, similar to L1 repeat, Tf subfamily, member 23 (LINE-element) (Mus musculus) (GB:NP_038603) |
| Transposons & pseudogenes | At2g23330 | retroelement pol polyprotein -related |
| Transposons & pseudogenes | At2g31080 | reverse transcriptase (RNA-dependent DNA polymerase) family protein similar to reverse transcriptase (Arabidopsis thaliana) GI:976278; contains Pfam profile PF00078: Reverse transcriptase (RNA-dependent DNA polymerase) |
| Transposons & pseudogenes | At2g31520 | reverse transcriptase (RNA-dependent DNA polymerase) family protein similar to reverse transcriptase (Arabidopsis thaliana) GI:976278; contains Pfam profiles PF00078: Reverse transcriptase (RNA-dependent DNA polymerase), PF03372: Endonuclease/Exonuclease/phosphatase family |
| Transposons & pseudogenes | At3g28915 | non-LTR retrotransposon family (LINE) |
| Transposons & pseudogenes | At3g30743 | CACTA-like transposase family (Tnp2/En/Spm) |
| Transposons & pseudogenes | At3g30744 | CACTA-like transposase family (Ptta/En/Spm) |
| Transposons & pseudogenes | At3g31420 | pseudogene, similar to L1 repeat, Tf subfamily, member 9 (LINE-element) (Mus musculus) (GB:NP_038607) |
| Transposons & pseudogenes | At3g32043 | non-LTR retrotransposon family (LINE) |
| Transposons & pseudogenes | At3g32130 | non-LTR reverse transcriptase -related |
| Transposons & pseudogenes | At3g32168 | gypsy-like retrotransposon family |
| Transposons & pseudogenes | At3g32250 | gypsy-like retrotransposon family (Athila) |
| Transposons & pseudogenes | At3g32360 | pseudogene, similar to ORF2 (Mus musculus) (GB:AAA39398) |
| Transposons & pseudogenes | At3g32377 | pseudogene, similar to aintegumenta-like protein |
| Transposons & pseudogenes | At3g33175 | pseudogene, hypothetical protein GB:AAD26889 |
| Transposons & pseudogenes | At3g42230 | hypothetical protein various predicted transposase like proteins |
| Transposons & pseudogenes | At3g43320 | hypothetical protein retrotransposon finger protein (clone DW15) - Arabidopsis thaliana, PIR:S65811 |
| Transposons & pseudogenes | At3g43360 | transposase -related mutator-like transposases |
| Transposons & pseudogenes | At3g44650 | reverse transcriptase (RNA-dependent DNA polymerase) family protein low similarity to reverse transcriptase (Arabidopsis thaliana) GI:976278; contains Pfam profile PF00078: Reverse transcriptase (RNA-dependent DNA polymerase) |
| Transposons & pseudogenes | At3g45520 | copia-related polyprotein COPIA PROTEIN - Drosophila melanogaster, SWISSPROT:COPI_DROME |
| Transposons & pseudogenes | At4g04000 | reverse transcriptase (RNA-dependent DNA polymerase) family protein similar to reverse transcriptase (Arabidopsis thaliana) GI:976278; contains Pfam profile PF00078: Reverse transcriptase (RNA-dependent DNA polymerase) |
| Transposons & pseudogenes | At4g04380 | polyprotein -related |
| Transposons & pseudogenes | At4g07420 | plant transposase (Ptta/En/Spm) family contains Pfam profile PF03004: Plant transposase (Ptta/En/Spm family) |
| Transposons & pseudogenes | At4g07770 | pseudogene, similar to L1 repeat, Tf subfamily, member 30 (LINE-element) (Mus musculus) (GB:NP_038605) |
| Transposons & pseudogenes | At4g07810 | polyprotein -related |
| Transposons & pseudogenes | At4g08090 | glycine-rich protein |
| Transposons & pseudogenes | At4g14470 | reverse transcriptase (RNA-dependent DNA polymerase) family protein low similarity to reverse transcriptase (Arabidopsis thaliana) GI:976278; contains Pfam profile PF00078: Reverse transcriptase (RNA-dependent DNA polymerase) |
| Transposons & pseudogenes | At4g15590 | reverse transcriptase (RNA-dependent DNA polymerase) family protein similar to reverse transcriptase (Arabidopsis thaliana) GI:976278; contains Pfam profile PF00078: Reverse transcriptase (RNA-dependent DNA polymerase) |
| Transposons & pseudogenes | At4g17450 | retrotransposon like protein |
| Transposons & pseudogenes | At4g18410 | MuDR transposable element - like protein mudrA protein - maize transposon MuDR, PIR2:S59141 |
| Transposons & pseudogenes | At4g19790 | LTR retrotransposon -related Brassica oleracea SLG-Sc and SLA-Sc genes and Melmoth retrotransposon sequence, PID:g2462935 |
| Transposons & pseudogenes | At5g20800 | pseudogene, putative protein predicted non-LTR reverse ranscriptase sequence fragments |
| Transposons & pseudogenes | At5g33400 | pseudogene |
| Transposons & pseudogenes | At5g35820 | copia-related retrotransposable element |
| Transposons & pseudogenes | At5g35935 | copia-like retrotransposon |

**Supplemental Table 3**

Putatively deleted genes in L*er* compared to Col-0. Genes were identified as deleted based on a 0.05 FDR threshold for SFP-detection. A transcript was considered deleted in L*er* if all of the Col hybridization values were above background and L*er* was not. In addition, 70% of the features had to be identified as SFPs (FDR 0.05). Genes were classified according to the functional categories obtained from the Munich Information Center for Protein Sequences (MIPS). The largest single group of the putatively deleted genes (39%) were transposable elements and pseudogenes. One third of the putative deletions (31%) was classified as ‘expressed’ and ‘hypothetical’ proteins, 15% belonged to the categories ‘intracellular signaling’, ‘disease, virulence and defense’ and 6% fell into the category ‘transcription’. Genes in the functional category termed ‘other’ include the following categories: Metabolism, protein synthesis, protein fate (folding, modification, destination) and cellular transport and transport mechanisms.
