# Supplementary material for: A High-Resolution Map of Arabidopsis Recombinant Inbred Lines by Whole-Genome Exon Array Hybridization
Source: PLoS Genet. 2006 Sep 15;2(9):e144. doi: 10.1371/journal.pgen.0020144 (PMC1564425; doi:10.1371/journal.pgen.0020144)
Supplement: Supplementary file 14 [file pgen.0020144.st004.doc]

| Chrom. | Ave. SFP no./ 50kb window | Ave. gene no./ 50kb window | Ave. probe no./ 50kb window | Ave. SFP/ gene ratio | Ave. SFP/ probe ratio |
| --- | --- | --- | --- | --- | --- |
| 1 | 5.3 | 11.0 | 150.8 | 0.48 | 0.035 |
| 2 | 5.6 | 10.4 | 134.9 | 0.54 | 0.041 |
| 3 | 5.4 | 11.1 | 146.2 | 0.48 | 0.037 |
| 4 | 6.2 | 11.2 | 148.5 | 0.55 | 0.042 |
| 5 | 5.5 | 11.2 | 151.1 | 0.49 | 0.036 |
| Genome-wide ave. | 5.6 | 11.0 | 146.3 | 0.51 | 0.038 |

**Supplemental Table 4.**

Genome-wide SFP distribution based on 50kb window size. Data was based on 1% FDR for SFP detection.
