# Supplementary material for: A High-Resolution Map of Arabidopsis Recombinant Inbred Lines by Whole-Genome Exon Array Hybridization
Source: PLoS Genet. 2006 Sep 15;2(9):e144. doi: 10.1371/journal.pgen.0020144 (PMC1564425; doi:10.1371/journal.pgen.0020144)
Supplement: Supplementary file 15 [file pgen.0020144.st005.doc]

| SFP-marker | chrom. | strand | pos.1 | pos.2 | gene ID | sequence (5'-3') |
| --- | --- | --- | --- | --- | --- | --- |
| C1_001 | 1 | Plus | 677,972 | 677,996 | At1g02980 | CATTTGACCCGGTTCAACGTATGCA |
| C1_002 | 1 | Plus | 1,238,972 | 1,238,996 | At1g04540 | TGGGCCGATGGTCTCAGAAGGATCT |
| C1_003 | 1 | Minus | 1,473,303 | 1,473,279 | At1g05120 | TTGAGGCTGGGACATTAATGAATAA |
| C1_004 | 1 | Minus | 1,560,971 | 1,560,947 | At1g05350 | CCTCAGGGTCAGGAGAAACAGAGGA |
| C1_005 | 1 | Minus | 1,933,783 | 1,933,759 | At1g06340 | AATCTCTCCACGTATTGATTCGCTG |
| C1_006 | 1 | Minus | 2,355,231 | 2,355,207 | At1g07640 | CTCAGGACAGCTAAGGACAAATCAT |
| C1_007 | 1 | Minus | 2,491,914 | 2,491,890 | At1g08030 | AGCTTCCGAGGGAGAGAACTTCGGT |
| C1_008 | 1 | Plus | 2,741,920 | 2,741,944 | At1g08620 | GAAGTAGTTCAGGTACACCGTCTTG |
| C1_009 | 1 | Plus | 2,928,345 | 2,928,369 | At1g09070 | CATGACGATAAGCACGATGGTGTTT |
| C1_010 | 1 | Minus | 3,226,096 | 3,226,072 | At1g09920 | CGTGTACATTGTGCTCGGAATTTAG |
| C1_011 | 1 | Plus | 4,120,516 | 4,120,540 | At1g12130 | AACTTCCCTCGGAGGAGCAAATGAT |
| C1_012 | 1 | Plus | 4,224,469 | 4,224,493 | At1g12410 | GATGCCATTATCAAGAATCGCCCTC |
| C1_013 | 1 | Plus | 4,261,744 | 4,261,768 | At1g12490 | TGTCGTGTGCGAAGTCGAACATTGA |
| C1_014 | 1 | Plus | 4,299,347 | 4,299,371 | At1g12630 | CTTGAATATGCCTAATCTGCTGGCT |
| C1_015 | 1 | Minus | 4,380,223 | 4,380,199 | At1g12850 | TCTCAACTCTCAACCTGCATTGGAG |
| C1_016 | 1 | Plus | 4,598,647 | 4,598,671 | At1g13400 | AATGATGTACCCACCGAGACATTTC |
| C1_017 | 1 | Minus | 4,614,592 | 4,614,568 | At1g13450 | GCAAAAAAGTGACACCACCACAGTA |
| C1_018 | 1 | Minus | 4,628,059 | 4,628,035 | At1g13500 | TAATGCCTCAGGGAATGAGAAGATA |
| C1_019 | 1 | Plus | 4,813,668 | 4,813,692 | At1g14040 | CTTCAGACACAGAAAAAGCACTTGC |
| C1_020 | 1 | Plus | 5,189,209 | 5,189,233 | At1g15080 | GTGGCCACATTTTGTTATCTGCAAT |
| C1_021 | 1 | Plus | 5,394,448 | 5,394,472 | At1g15680 | GACTTGAAGACACACAAGCTTACAC |
| C1_022 | 1 | Minus | 5,547,098 | 5,547,074 | At1g16210 | GTGGCTCTGGCTAAGAAAGCAGAAG |
| C1_023 | 1 | Plus | 5,854,427 | 5,854,451 | At1g17130 | ATTCCCGTAAAGAAGACGTCATTGG |
| C1_024 | 1 | Plus | 5,870,115 | 5,870,139 | At1g17170 | AGACTTGGCCCGACAATAACCCTCT |
| C1_025 | 1 | Plus | 6,034,729 | 6,034,753 | At1g17550 | GAGGTACATACTTCTTCGTCATTCA |
| C1_026 | 1 | Minus | 6,150,366 | 6,150,342 | At1g17870 | CCATGTGGAAGACTTGAAGGAGGTC |
| C1_027 | 1 | Minus | 6,229,150 | 6,229,126 | At1g18100 | CCCGGAGGCACAAATCCCTCAAGAG |
| C1_028 | 1 | Plus | 6,346,214 | 6,346,238 | At1g18440 | GGAATCAGAGTTACTTCAAGCTTTA |
| C1_029 | 1 | Plus | 6,493,270 | 6,493,294 | At1g18830 | TTTGAATCCTCCTCGAGAACTAAAG |
| C1_030 | 1 | Plus | 6,944,593 | 6,944,617 | At1g20020 | CAATACGCAGCTGAGTTATGGGAGT |
| C1_031 | 1 | Minus | 7,072,485 | 7,072,461 | At1g20400 | GATCGAGATCAAATCTACCGAGCTG |
| C1_032 | 1 | Minus | 7,259,483 | 7,259,459 | At1g20870 | GGGATCAACTACAAGAGGCGAAAAA |
| C1_033 | 1 | Plus | 7,635,391 | 7,635,415 | At1g21730 | GTTCTGTCAAAGCTCACGATGCAAC |
| C1_034 | 1 | Minus | 7,716,840 | 7,716,816 | At1g21940 | AAGAGTGTGACGTGCAGCTGCTCGT |
| C1_035 | 1 | Minus | 7,793,657 | 7,793,633 | At1g22080 | TGATTAACCGCGAACAGGTGGCGTT |
| C1_036 | 1 | Minus | 7,901,999 | 7,901,975 | At1g22380 | TTGTCACGAGAGAGGATGTCCCTCC |
| C1_037 | 1 | Minus | 7,953,845 | 7,953,821 | At1g22520 | TTAGCAGCTCCCAAGAGTACGGAGA |
| C1_038 | 1 | Plus | 8,056,875 | 8,056,899 | At1g22760 | ATCGAGCGCAGTTGTAATGAGGGAC |
| C1_039 | 1 | Plus | 8,096,334 | 8,096,358 | At1g22880 | TCCCTCAAACCCTGGCTCTGATGTA |
| C1_040 | 1 | Plus | 8,144,798 | 8,144,822 | At1g23000 | TCACATGCAACAAACATGTCTAGTG |
| C1_041 | 1 | Plus | 8,200,954 | 8,200,978 | At1g23130 | CTAACCGAATCAATGGACGAAATGC |
| C1_042 | 1 | Plus | 8,325,624 | 8,325,648 | At1g23450 | GGAAGAGACCTTCTATACGGCCATT |
| C1_043 | 1 | Minus | 8,398,720 | 8,398,696 | At1g23740 | CAACTGCGAGTACAGAAAAGCTAGA |
| C1_044 | 1 | Plus | 8,637,064 | 8,637,088 | At1g24340 | ACAGAGTGATACTTGCTGGTGATGC |
| C1_045 | 1 | Plus | 8,694,377 | 8,694,401 | At1g24530 | TCCGGTCATACCAAGCCGGTTAAGT |
| C1_046 | 1 | Minus | 8,714,515 | 8,714,491 | At1g24590 | CGGATTCGGTCAGGTTAAATATCAT |
| C1_047 | 1 | Plus | 8,976,669 | 8,976,693 | At1g25550 | TACACCCAGAAAATGAAGAGATGTC |
| C1_048 | 1 | Plus | 9,190,322 | 9,190,346 | At1g26590 | CTCATTGATCAGGTTCAGGAATAGT |
| C1_049 | 1 | Minus | 9,372,145 | 9,372,121 | At1g26990 | AAGTCCACGGGTATCCTCCAGGTCA |
| C1_050 | 1 | Minus | 9,512,854 | 9,512,830 | At1g27385 | TCTCCAGCATCAGTTCCAGTCCATG |
| C1_051 | 1 | Minus | 9,843,658 | 9,843,634 | At1g28180 | GACAGCGATAGTGTTTGTGAATACC |
| C1_052 | 1 | Plus | 10,000,010 | 10,000,034 | At1g28440 | AAGTGGGTTTGCTCTACATTGGACC |
| C1_053 | 1 | Plus | 10,150,189 | 10,150,213 | At1g29070 | GCCTGTTTCCCTAGCGTCTCCATTT |
| C1_054 | 1 | Plus | 10,208,206 | 10,208,230 | At1g29200 | TTATCTGGTAATGATCTCTTACTCG |
| C1_055 | 1 | Plus | 10,344,428 | 10,344,452 | At1g29600 | GGGTCAAATACTGACTCATACGCGC |
| C1_056 | 1 | Plus | 10,434,381 | 10,434,405 | At1g29800 | AAATCGGGACTGGACTTGTGGTTGC |
| C1_057 | 1 | Plus | 10,466,659 | 10,466,683 | At1g29890 | GGAGATTTTCCCTGACACTCTTTGC |
| C1_058 | 1 | Minus | 10,584,729 | 10,584,705 | At1g30120 | ACCGTTCGACCTTCACACAATTGGA |
| C1_059 | 1 | Minus | 10,641,882 | 10,641,858 | At1g30240 | CTTTTGGCAGCGAAAAACACAACAC |
| C1_060 | 1 | Plus | 10,701,242 | 10,701,266 | At1g30340 | GAGGATTGGTCCTTCATCAGTTTCG |
| C1_061 | 1 | Plus | 10,816,416 | 10,816,440 | At1g30530 | CTCCAAGAAGATTTCTCCATGAAAG |
| C1_062 | 1 | Plus | 10,995,336 | 10,995,360 | At1g30890 | TGTTCTGTTTCCTTTCCTACACCGG |
| C1_063 | 1 | Minus | 11,010,486 | 11,010,462 | At1g30920 | TTACATGGCGAAAAACACCAAATGA |
| C1_064 | 1 | Minus | 11,023,206 | 11,023,182 | At1g30940 | ATAAGTTGTCTCCTGCCGATTTCCA |
| C1_065 | 1 | Minus | 11,039,647 | 11,039,623 | At1g30960 | ATCAGAAGTCGCTTAATGTCGGCAT |
| C1_066 | 1 | Minus | 11,106,254 | 11,106,230 | At1g31120 | TATATGGAAACATGACCCAAGCGTT |
| C1_067 | 1 | Plus | 11,251,764 | 11,251,788 | At1g31420 | GAGTGCTCTCTGGATTTTCTAAGAA |
| C1_068 | 1 | Plus | 11,344,562 | 11,344,586 | At1g31680 | GGAAGTTGTGGTCACCTCCTCAACG |
| C1_069 | 1 | Plus | 11,466,617 | 11,466,641 | At1g31930 | TGACGAATCGAAACGGTCTAAATTA |
| C1_070 | 1 | Minus | 11,513,039 | 11,513,015 | At1g32020 | AGAACTGTGACTTCCCTCCTAATGG |
| C1_071 | 1 | Plus | 11,554,788 | 11,554,812 | At1g32120 | CCCTGATAGAAGTTCTGATGAGCCT |
| C1_072 | 1 | Plus | 11,570,253 | 11,570,277 | At1g32160 | TGTCTCTGGACTTGCGATGTTGGTT |
| C1_073 | 1 | Plus | 11,653,247 | 11,653,271 | At1g32300 | CGACGGAATGGACCAACCAAATTCT |
| C1_074 | 1 | Plus | 11,944,258 | 11,944,282 | At1g32950 | CAGGTCTCATACTCGACATGGACTC |
| C1_075 | 1 | Plus | 12,160,804 | 12,160,828 | At1g33530 | GGTCCAATGCCACTTTCTTGCATTC |
| C1_076 | 1 | Minus | 12,404,741 | 12,404,717 | At1g34080 | TTTTCTTGATACTCTCGTACGTATG |
| C1_077 | 1 | Plus | 12,537,659 | 12,537,683 | At1g34355 | TTTGTCCGTAGTACGCAGCAGAGTG |
| C1_078 | 1 | Plus | 12,849,254 | 12,849,278 | At1g35130 | ATCATGGTTGTGATAACTCGAAGCA |
| C1_079 | 1 | Minus | 13,119,968 | 13,119,944 | At1g35570 | TTCTCCTGTGACTTTGCTTCGATAG |
| C1_080 | 1 | Plus | 13,218,466 | 13,218,490 | At1g35700 | TTTATCCATAACTGTATTATTGGCG |
| C1_081 | 1 | Minus | 13,280,893 | 13,280,869 | At1g35780 | AGTCGTATTTGGAGGACAAGTTACA |
| C1_082 | 1 | Minus | 13,579,138 | 13,579,114 | At1g36200 | GGCAACTGCAAATTATCCATGGATT |
| C1_083 | 1 | Plus | 14,504,020 | 14,504,044 | NO_GENE | ATCAATACATTTGACCACACGGTCG |
| C1_084 | 1 | Minus | 15,343,526 | 15,343,502 | NO_GENE | ACTGTTACCCGGTGAACACACTTGA |
| C1_085 | 1 | Plus | 15,710,697 | 15,710,721 | At1g43030 | GATGCCCGGTATCGTGGTGTTAAAA |
| C1_086 | 1 | Plus | 15,861,767 | 15,861,791 | At1g43280 | GAAACAGCTGCCATACTCAATAGGT |
| C1_087 | 1 | Plus | 16,008,415 | 16,008,439 | At1g43690 | TCTCCTGTTAACAAAGCTAGGGTTT |
| C1_088 | 1 | Plus | 16,105,710 | 16,105,734 | At1g43790 | GGTTGTGTCATGTTCTTGGCGTTTC |
| C1_089 | 1 | Minus | 16,192,557 | 16,192,533 | At1g43930 | TACTCATCAAGAACACTTTGCACGG |
| C1_090 | 1 | Plus | 16,323,712 | 16,323,736 | At1g44150 | CGGCTGTACCCGTAGCAGAGTCTTC |
| C1_091 | 1 | Plus | 16,343,605 | 16,343,629 | At1g44190 | GGACGCGGAGGTGATGGTTTAGGAG |
| C1_092 | 1 | Minus | 16,512,992 | 16,512,968 | At1g44920 | CCGTGTTTGTTCAAAGGTGATGCTA |
| C1_093 | 1 | Plus | 16,890,812 | 16,890,836 | At1g47220 | AGGATATACTTCTCGCGCTTGAGTT |
| C1_094 | 1 | Plus | 16,914,453 | 16,914,477 | At1g47270 | GTATAATGCACAGTATCCCGATTTC |
| C1_095 | 1 | Plus | 16,935,612 | 16,935,636 | At1g47320 | TTCAAGCCAGAAATTTATGCAAGAC |
| C1_096 | 1 | Plus | 16,983,978 | 16,984,002 | At1g47430 | ACAGTTATACTGAAGGCACAAGAGG |
| C1_097 | 1 | Plus | 17,108,033 | 17,108,057 | At1g47650 | TGCTTTTCAGGTCTCTGAGGCAATG |
| C1_098 | 1 | Plus | 17,425,090 | 17,425,114 | At1g48280 | GAATATGGCCGGAAATCTGTCGTAG |
| C1_099 | 1 | Minus | 17,590,625 | 17,590,601 | At1g48680 | CATTAACCGATGTTGTTAAAGAGGC |
| C1_100 | 1 | Plus | 17,637,552 | 17,637,576 | At1g48800 | CCACTACTATTCCTCTGGCGTCTGG |
| C1_101 | 1 | Minus | 17,670,559 | 17,670,535 | At1g48890 | GGTTGGGTGCCCTAACGCTTGACGC |
| C1_102 | 1 | Plus | 17,898,744 | 17,898,768 | At1g49470 | CATCTACAGACAATAATAGAAGGAG |
| C1_103 | 1 | Plus | 18,100,525 | 18,100,549 | At1g49980 | TCAATTGGAGACATGGCTGACATGT |
| C1_104 | 1 | Plus | 18,367,694 | 18,367,718 | At1g50690 | TCATAAACCCGAAACCGACGGAGAT |
| C1_105 | 1 | Minus | 18,851,051 | 18,851,027 | At1g51870 | CTTATTGAATGCACAGTGTGGAGCG |
| C1_106 | 1 | Minus | 18,953,470 | 18,953,446 | At1g52070 | CAATGGCAAGATGGTGATTGGCTTC |
| C1_107 | 1 | Minus | 19,012,614 | 19,012,590 | At1g52180 | CAGACTCATCGTTGGTGCTAACATT |
| C1_108 | 1 | Plus | 19,072,718 | 19,072,742 | At1g52320 | CCTGGAACTACTTTAGAAGATACTC |
| C1_109 | 1 | Plus | 19,087,935 | 19,087,959 | At1g52360 | GTCAGCCAAGTTTGTAGCACGAAAG |
| C1_110 | 1 | Plus | 19,105,089 | 19,105,113 | At1g52400 | TACGGAGAAGACCTTGGAGAGAAGC |
| C1_111 | 1 | Plus | 19,314,768 | 19,314,792 | At1g52950 | TTTGATAGTTTTGTCGAGCCAATCA |
| C1_112 | 1 | Plus | 19,484,558 | 19,484,582 | At1g53330 | TAAGCTACAACATGATACTTGGTGT |
| C1_113 | 1 | Minus | 19,603,993 | 19,603,969 | At1g53630 | ATCCGGAGCCGGTATTTGAGACGGA |
| C1_114 | 1 | Minus | 19,735,358 | 19,735,334 | At1g53950 | GAGAACCAACACCTACGAGGTTAAG |
| C1_115 | 1 | Minus | 20,160,112 | 20,160,088 | At1g55140 | CAAGTGGTTCTTCAACAGAGAGAAT |
| C1_116 | 1 | Minus | 20,511,085 | 20,511,061 | At1g55940 | AGAAGAAGCAACATCGGATGACTCA |
| C1_117 | 1 | Plus | 20,795,966 | 20,795,990 | At1g56600 | ACTTATCATAACCTCTTGGAGACGG |
| C1_118 | 1 | Plus | 21,194,095 | 21,194,119 | At1g58260 | GTAATGACCCCATCATCCAAGAAAG |
| C1_119 | 1 | Minus | 21,291,097 | 21,291,073 | At1g58410 | CTCACATCTAAAGAGAGGAAAGATG |
| C1_120 | 1 | Minus | 21,562,444 | 21,562,420 | At1g59640 | GCAACAGATGGAACCAACGAAAGAT |
| C1_121 | 1 | Minus | 21,744,665 | 21,744,641 | At1g59990 | CGTTTAAGCTCTCTCAATCGATCTG |
| C1_122 | 1 | Plus | 22,231,103 | 22,231,127 | At1g61230 | CACATGGATTCCTGATTGTAGATTA |
| C1_123 | 1 | Minus | 22,703,751 | 22,703,727 | At1g62340 | TAATCCTTCTTGGACTCCAGCCATG |
| C1_124 | 1 | Plus | 22,781,137 | 22,781,161 | At1g62480 | CGCCTGTCGAAGTAACAACTAAAGA |
| C1_125 | 1 | Plus | 22,832,344 | 22,832,368 | At1g62600 | ATACGTATATTGAGCGGCCTGGTTA |
| C1_126 | 1 | Minus | 22,927,044 | 22,927,020 | At1g62860 | TTAGAGCTTTTCAGCGAGATGTGTC |
| C1_127 | 1 | Plus | 23,023,856 | 23,023,880 | At1g63040 | AGCTATTGAACATGCCTGGTTTGCT |
| C1_128 | 1 | Minus | 23,360,995 | 23,360,971 | At1g63870 | GAAAGCCATGTTCACTGACAACAAC |
| C1_129 | 1 | Plus | 23,475,661 | 23,475,685 | At1g64180 | CCACGTGGCGATGAAACTGAAAAAC |
| C1_130 | 1 | Plus | 23,491,460 | 23,491,484 | At1g64240 | CGCGCCTCAAGAAAATGATGAATGG |
| C1_131 | 1 | Minus | 23,597,105 | 23,597,081 | At1g64470 | CAGTGGTCGAGAGCTTGCCAGGAAT |
| C1_132 | 1 | Minus | 23,849,372 | 23,849,348 | At1g65130 | ACTTGTTGCAATCCTCGAGTTTTGT |
| C1_133 | 1 | Plus | 24,041,941 | 24,041,965 | At1g65590 | GTTGGTCTGCAACACCGCGAATAGC |
| C1_134 | 1 | Minus | 24,058,670 | 24,058,646 | At1g65630 | CAAACATCTATATAAGCTTGTAGAG |
| C1_135 | 1 | Plus | 24,147,278 | 24,147,302 | At1g65850 | AGCTCTGGCAAAAATGGGAACAACC |
| C1_136 | 1 | Minus | 24,309,029 | 24,309,005 | At1g66200 | GACCTGTGACCGATCCATCAAAACT |
| C1_137 | 1 | Minus | 24,348,585 | 24,348,561 | At1g66260 | CTCTATGCTGAAATTGGAGAGCTGA |
| C1_138 | 1 | Minus | 24,501,728 | 24,501,704 | At1g66610 | CTGAATATATCTTGTGTCCGATGGG |
| C1_139 | 1 | Plus | 24,554,169 | 24,554,193 | At1g66760 | CAAGCAACTTCTACAATGATCGTCG |
| C1_140 | 1 | Minus | 24,638,173 | 24,638,149 | At1g66960 | TATGACCGAGCGTGAATATACAGAA |
| C1_141 | 1 | Minus | 24,653,369 | 24,653,345 | At1g66980 | CTGGCCAATGCCTCAAATAGTGAAG |
| C1_142 | 1 | Minus | 24,671,523 | 24,671,499 | At1g67040 | AATTATCAGGCGATCGCAAGTCTTA |
| C1_143 | 1 | Minus | 24,715,862 | 24,715,838 | At1g67110 | TGTAGAGATCGGTCGGAGTAGCTCA |
| C1_144 | 1 | Plus | 24,813,487 | 24,813,511 | At1g67240 | CCATGAAGCGTATTGCTAAGCTGTG |
| C1_145 | 1 | Plus | 24,968,247 | 24,968,271 | At1g67550 | CTTCGGAGCAAAACCAGAAATGATA |
| C1_146 | 1 | Plus | 25,412,071 | 25,412,095 | At1g68600 | TTTCGTCATCTTTCTCAAAGAGCCG |
| C1_147 | 1 | Minus | 25,430,183 | 25,430,159 | At1g68660 | CAACTTCAACAAGAGGGAATATGTG |
| C1_148 | 1 | Minus | 25,597,099 | 25,597,075 | At1g69020 | GTAGTAAGCCATGGTACACAGCAGA |
| C1_149 | 1 | Minus | 25,800,828 | 25,800,804 | At1g69545 | TCCAATTCGGCAGGAAAAAATGGGA |
| C1_150 | 1 | Minus | 25,813,994 | 25,813,970 | At1g69570 | CTTCATCATGTTTAGGAAAACGGTC |
| C1_151 | 1 | Plus | 25,873,199 | 25,873,223 | At1g69700 | CAATTCAATGGAGCAGAACATATCT |
| C1_152 | 1 | Minus | 25,880,819 | 25,880,795 | At1g69730 | TATGCCTTGTGGAGATATGCAACTG |
| C1_153 | 1 | Minus | 25,946,148 | 25,946,124 | At1g69840 | TTCTGCAGATCAAGCGTGCTGAGGG |
| C1_154 | 1 | Minus | 26,044,967 | 26,044,943 | At1g70070 | AGGTTCAACTTATATGCCTATCAGC |
| C1_155 | 1 | Plus | 26,278,043 | 26,278,067 | At1g70610 | CTATGGACAGTGGACGAGTCGTCGA |
| C1_156 | 1 | Plus | 26,477,613 | 26,477,637 | At1g71140 | CCTTGAGTGGTGGTCATTCGAATTT |
| C1_157 | 1 | Minus | 26,735,374 | 26,735,350 | At1g71960 | TCCACAGACTTTTAAAAGAACGGCG |
| C1_158 | 1 | Plus | 26,787,238 | 26,787,262 | At1g72120 | GCAAATCGATCTCAAGAATCTCCAG |
| C1_159 | 1 | Minus | 26,854,400 | 26,854,376 | At1g72270 | AACTGGGCTACGAAACTCTCGGGAT |
| C1_160 | 1 | Minus | 27,040,076 | 27,040,052 | At1g72760 | AATATTGTTGCTGATGCTCCACAAT |
| C1_161 | 1 | Minus | 27,462,130 | 27,462,106 | At1g73960 | ATGAACTCGATGGAATGTCTGATCA |
| C1_162 | 1 | Plus | 27,957,468 | 27,957,492 | At1g75420 | AGATCTAGCTTTGAAACCTGTAGCA |
| C1_163 | 1 | Minus | 28,070,426 | 28,070,402 | At1g75680 | TAGATCATCCATGCTGGGAGAGACC |
| C1_164 | 1 | Plus | 28,246,532 | 28,246,556 | At1g76200 | GGAGTCACTGTCCACACTCCCAAGA |
| C1_165 | 1 | Minus | 28,802,247 | 28,802,223 | At1g77590 | TGCCTCGAGATAATAGACCGAAAAA |
| C1_166 | 1 | Minus | 29,056,323 | 29,056,299 | At1g78140 | TACAGTGAACCGATGCCTCTTTCCA |
| C1_167 | 1 | Plus | 29,187,692 | 29,187,716 | At1g78500 | AGTTTCCGGAGCACAGAAGGGAAGA |
| C1_168 | 1 | Minus | 29,465,012 | 29,464,988 | At1g79250 | CTCACCTGGTTCCAACATGACCGAG |
| C1_169 | 1 | Plus | 29,514,852 | 29,514,876 | At1g79380 | TCTTAGCATGTTACAAGAGAATTGC |
| C1_170 | 1 | Minus | 29,565,719 | 29,565,695 | At1g79520 | AACACATAGACACAGTGAGAGCCTA |
| C1_171 | 1 | Plus | 29,802,537 | 29,802,561 | At1g80150 | CGTTATACAAGCACGAGAGGTGTGT |
| C1_172 | 1 | Plus | 30,064,780 | 30,064,804 | At1g80940 | AGCATGCGTAGAATAGCTGTCCAAG |
| C2_001 | 2 | Minus | 246,863 | 246,839 | At2g01550 | TCAAATAGTTACTCTGCTCTGAATG |
| C2_002 | 2 | Minus | 337,120 | 337,096 | At2g01780 | GCATCTTCACAAGATTGAACCTAAG |
| C2_003 | 2 | Minus | 356,899 | 356,875 | At2g01820 | CTCACCGTCCAATGGAAACCAACTG |
| C2_004 | 2 | Plus | 385,971 | 385,995 | At2g01850 | ATCGGGCTAGGTACAATGTTGCTTT |
| C2_005 | 2 | Minus | 452,335 | 452,311 | At2g01970 | TTTGGTCGTTATCTATGCTCTCACA |
| C2_006 | 2 | Minus | 475,213 | 475,189 | At2g02010 | GCCTGAGAACTCAATCCCAAAAGAA |
| C2_007 | 2 | Plus | 495,314 | 495,338 | At2g02060 | TACCATTTCACATGTCAAAAGCCAC |
| C2_008 | 2 | Minus | 531,792 | 531,768 | At2g02110 | CTCCTCCGTGGAATGAATTGAAATG |
| C2_009 | 2 | Plus | 701,728 | 701,752 | At2g02580 | CGGCCTCAAACTTCTTCCCAAATGG |
| C2_010 | 2 | Plus | 945,842 | 945,866 | At2g03140 | TGTTGTCCCGGGTAGAAGACAAAAG |
| C2_011 | 2 | Minus | 1,148,928 | 1,148,904 | At2g03760 | GGAAAAAGCTAGCTCCTGAGGAAAC |
| C2_012 | 2 | Minus | 1,231,954 | 1,231,930 | At2g03950 | ACCCGAATTTGTATCAGGACAATAA |
| C2_013 | 2 | Minus | 1,549,463 | 1,549,439 | At2g04470 | CTATAGCGGAAGACGAATGGCTCAA |
| C2_014 | 2 | Plus | 1,797,991 | 1,798,015 | At2g05060 | GATCATAGAGCCTCTCGTGAAGAGT |
| C2_015 | 2 | Plus | 1,970,523 | 1,970,547 | At2g05390 | CTTGCTCAGATAATAGCTGGTATTT |
| C2_016 | 2 | Minus | 2,039,068 | 2,039,044 | At2g05550 | AGCCCCGGGAAAAGTCATGTAGGAT |
| C2_017 | 2 | Plus | 2,296,819 | 2,296,843 | At2g05950 | AGATGCTTTTCACAAAACGACTGAA |
| C2_018 | 2 | Minus | 2,313,359 | 2,313,335 | At2g05980 | ATAGAAGCACAGATGGCGATCGCTC |
| C2_019 | 2 | Minus | 2,674,698 | 2,674,674 | At2g06710 | GTTGTACATGCTGAACTGTCCTACG |
| C2_020 | 2 | Minus | 3,231,240 | 3,231,216 | At2g07670 | AATGGAAGCTTGTCGCCTGAAGCGG |
| C2_021 | 2 | Minus | 4,426,618 | 4,426,594 | At2g11240 | AGAAGAGACCTTACAGGCTGGTTTC |
| C2_022 | 2 | Minus | 4,934,983 | 4,934,959 | At2g12380 | CGGCTGCAAGTAGAGCTGGATAACC |
| C2_023 | 2 | Minus | 5,011,599 | 5,011,575 | At2g12470 | TCACGAAGATTAGTCACTGGTTTTT |
| C2_024 | 2 | Plus | 5,240,350 | 5,240,374 | At2g12880 | GGAAATCAAGACCATCGTTTCAATT |
| C2_025 | 2 | Plus | 5,373,288 | 5,373,312 | At2g13140 | AAACCGCAGAGAATATACCGAACCA |
| C2_026 | 2 | Minus | 5,481,221 | 5,481,197 | At2g13335 | GAAAGTGACATCCTTAACGGTCCAA |
| C2_027 | 2 | Minus | 5,758,479 | 5,758,455 | At2g13860 | TCCACAGACAGATGGACAATATGAG |
| C2_028 | 2 | Minus | 5,844,375 | 5,844,351 | At2g14030 | AGTTGAGTCTCTGAACCCAGTCTTG |
| C2_029 | 2 | Plus | 6,045,222 | 6,045,246 | At2g14370 | CTTTGCCTGAGAAATAACAGTTTTG |
| C2_030 | 2 | Plus | 6,206,074 | 6,206,098 | At2g14640 | AAGTAATCAACTTGCGCGTTGGATG |
| C2_031 | 2 | Minus | 6,233,920 | 6,233,896 | At2g14690 | TACACGAAGCAGAGCGATGGCTTAG |
| C2_032 | 2 | Plus | 6,243,515 | 6,243,539 | At2g14700 | CGACGAAGCAGTGGAATGCGCTTCA |
| C2_033 | 2 | Plus | 6,320,972 | 6,320,996 | At2g14840 | AATGTCAAAAGTTCGGCTTAAACAG |
| C2_034 | 2 | Plus | 6,387,048 | 6,387,072 | At2g14940 | GGCTGTCTCTAAACTTTCCCGCAAC |
| C2_035 | 2 | Plus | 6,513,866 | 6,513,890 | At2g15130 | GAGATGCAACGGACTTCACGTGGCG |
| C2_036 | 2 | Minus | 6,622,089 | 6,622,065 | At2g15330 | GGCTTCTTTCCCATGACCCAACTGA |
| C2_037 | 2 | Minus | 6,657,992 | 6,657,968 | At2g15390 | GAACCTGACGCATGGGGAACTGAAT |
| C2_038 | 2 | Minus | 6,674,030 | 6,674,006 | At2g15420 | TTCCCGTGATGATGCGGAAAAGGGA |
| C2_039 | 2 | Minus | 6,820,422 | 6,820,398 | At2g15770 | GACTCAAACAGTTCCGGTTCGGATT |
| C2_040 | 2 | Plus | 6,907,029 | 6,907,053 | At2g16000 | ATTGGACTGTCACACAGTTAGAGAA |
| C2_041 | 2 | Plus | 7,024,752 | 7,024,776 | At2g16350 | TACATCAACAGATCAACCAACAGGG |
| C2_042 | 2 | Plus | 7,120,812 | 7,120,836 | At2g16550 | ATGTACCAGATTCTGGCGTTGTGGC |
| C2_043 | 2 | Minus | 7,174,734 | 7,174,710 | At2g16670 | GCAACTTCAACGACATCGGCTTCGT |
| C2_044 | 2 | Minus | 7,258,965 | 7,258,941 | At2g16870 | AAATCTGTGATACGAAGGCTAGACA |
| C2_045 | 2 | Plus | 7,351,270 | 7,351,294 | At2g17033 | TAATGTTCCTTTCTCCATAAGGACG |
| C2_046 | 2 | Minus | 7,637,713 | 7,637,689 | At2g17700 | CAGCGACTTCAAGAATCTAATTACG |
| C2_047 | 2 | Minus | 8,015,333 | 8,015,309 | At2g18580 | CTGGTTCATCATATCCTACTGCTAG |
| C2_048 | 2 | Plus | 8,194,829 | 8,194,853 | At2g19020 | GAGAGTCGTCGGAAGCATCTAGACT |
| C2_049 | 2 | Minus | 8,296,313 | 8,296,289 | At2g19230 | TCTCTTATGCTGTATCGAAGATGGG |
| C2_050 | 2 | Plus | 8,595,413 | 8,595,437 | At2g20020 | GTACGGAGACTTGGAGGCAAAATCA |
| C2_051 | 2 | Minus | 9,142,355 | 9,142,331 | At2g21460 | GACTGAACAGCGAGGCAACAACAAC |
| C2_052 | 2 | Plus | 9,330,394 | 9,330,418 | At2g22060 | ACCATTGCAATGTCGGGATACAATG |
| C2_053 | 2 | Plus | 9,397,639 | 9,397,663 | At2g22210 | AAGTTATGACGGATTGCTCAGATCT |
| C2_054 | 2 | Plus | 9,529,120 | 9,529,144 | At2g22540 | AAACTGTTCGAGTTCTGTAGCTCCA |
| C2_055 | 2 | Minus | 9,876,522 | 9,876,498 | At2g23330 | GACGCTGTTCTCGACAAACTCATCA |
| C2_056 | 2 | Plus | 10,123,367 | 10,123,391 | At2g23900 | CATACCGCAGTTTTGACAGAATTTG |
| C2_057 | 2 | Plus | 10,128,301 | 10,128,325 | At2g23910 | CTAAACAAGCTGGTGGAAGCTGGAT |
| C2_058 | 2 | Plus | 10,163,350 | 10,163,374 | At2g24010 | TTTCTACTGGCTTACAGAGTCCCCT |
| C2_059 | 2 | Minus | 10,218,177 | 10,218,153 | At2g24160 | CTTAGTAGTCTATACCTTGCTGATA |
| C2_060 | 2 | Minus | 10,296,027 | 10,296,003 | At2g24330 | CTCTAAAGCCAATGGAAAGTGAAGT |
| C2_061 | 2 | Minus | 10,462,557 | 10,462,533 | At2g24700 | TCCGAAGACGTTCATTATGTCAAAT |
| C2_062 | 2 | Minus | 10,644,335 | 10,644,311 | At2g25130 | TTTTCTCTTGAACACGTTGGGAGAC |
| C2_063 | 2 | Plus | 10,811,639 | 10,811,663 | At2g25530 | GGCATCCTAGTTTTGACATCTCTAC |
| C2_064 | 2 | Minus | 10,998,442 | 10,998,418 | At2g25910 | GTGACATTAGCTTCGATTGGGTTAC |
| C2_065 | 2 | Minus | 11,026,393 | 11,026,369 | At2g25980 | CCCGCCTTATGGAATGGATGCTGGC |
| C2_066 | 2 | Plus | 11,110,484 | 11,110,508 | At2g26220 | CTATGTTTCTTGAGGAGGTGAGCTC |
| C2_067 | 2 | Plus | 11,229,058 | 11,229,082 | At2g26520 | GTATCGACGGTCACATTCATCCTCA |
| C2_068 | 2 | Minus | 11,506,039 | 11,506,015 | At2g27070 | TTTCAATACTAACATGATGAGCAGC |
| C2_069 | 2 | Plus | 11,656,324 | 11,656,348 | At2g27360 | AATGGATATCCGAGGGCGTACTAAC |
| C2_070 | 2 | Minus | 11,768,700 | 11,768,676 | At2g27730 | GAACAGAAACTATGCGGTTGTGGCA |
| C2_071 | 2 | Minus | 11,898,947 | 11,898,923 | At2g28060 | AAGAGATCCTCCAGCAGTACCGCCA |
| C2_072 | 2 | Minus | 12,082,704 | 12,082,680 | At2g28380 | TTAAGGATTCATCGAAAGGAGAACC |
| C2_073 | 2 | Minus | 12,112,359 | 12,112,335 | At2g28450 | TGGCCACTGAGTTTGAAGAATTGGC |
| C2_074 | 2 | Minus | 12,125,766 | 12,125,742 | At2g28480 | TGCAATAATAGTAAGCCCGGCCAAG |
| C2_075 | 2 | Plus | 12,407,784 | 12,407,808 | At2g28990 | CCTCACATAGTGGAATGGGTCAGTT |
| C2_076 | 2 | Minus | 12,679,412 | 12,679,388 | At2g29830 | TTTCATTTTGGATCTTGAGTGTTGT |
| C2_077 | 2 | Minus | 12,850,184 | 12,850,160 | At2g30240 | ATTTCTTTGAGATAACAGAGAGCCA |
| C2_078 | 2 | Minus | 12,947,060 | 12,947,036 | At2g30500 | AAGACGGTCAAGTGAGCTTACGGAA |
| C2_079 | 2 | Plus | 13,066,216 | 13,066,240 | At2g30780 | ATGCTCAAGAAGACTTTGTGGAAGC |
| C2_080 | 2 | Minus | 13,357,782 | 13,357,758 | At2g31480 | TTGTTGGAAACACAATCCTTGATGA |
| C2_081 | 2 | Plus | 13,694,092 | 13,694,116 | At2g32370 | TCATTTGTGCTTTATGCGCCTGTTG |
| C2_082 | 2 | Minus | 13,923,935 | 13,923,911 | At2g32940 | TTATAGGTCTAAGTTCGAAACCGTG |
| C2_083 | 2 | Minus | 14,159,531 | 14,159,507 | At2g33550 | CTCCGGCGTCAGACGAGGGATTGTT |
| C2_084 | 2 | Minus | 14,431,132 | 14,431,108 | At2g34310 | AAAGTCCCGGATGGAGCATGAGCAT |
| C2_085 | 2 | Minus | 14,890,067 | 14,890,043 | At2g35600 | CTTACACAAGAATTGATTCAGCAAG |
| C2_086 | 2 | Minus | 15,148,159 | 15,148,135 | At2g36250 | TTTTGAAGAGCCATCTGCTCCGAGT |
| C2_087 | 2 | Plus | 15,189,091 | 15,189,115 | At2g36350 | TTGCAGTATGGCTCTTTAGGACTGC |
| C2_088 | 2 | Minus | 15,278,275 | 15,278,251 | At2g36560 | AAGATGAGTCCAACAACGCTATGGC |
| C2_089 | 2 | Minus | 15,316,552 | 15,316,528 | At2g36670 | CCTTCGTTCCCTAATGTTGGCGGCG |
| C2_090 | 2 | Plus | 15,451,765 | 15,451,789 | At2g36910 | CAAATCAGGGTAGTGATGGCTTTCG |
| C2_091 | 2 | Plus | 15,518,216 | 15,518,240 | At2g37050 | GACTCAAATGGTCACCTGATAATCA |
| C2_092 | 2 | Minus | 15,789,817 | 15,789,793 | At2g37800 | TGGGTCACCACAACCGCAATTTTAC |
| C2_093 | 2 | Plus | 16,029,705 | 16,029,729 | At2g38390 | AAGTGCTTTTCTCAACTCCAGGAGC |
| C2_094 | 2 | Minus | 16,103,646 | 16,103,622 | At2g38630 | ATCAATCGAAGATGGCACTCTTCTG |
| C2_095 | 2 | Plus | 16,332,387 | 16,332,411 | At2g39230 | TATACAACGATGATCGACGGGTTGT |
| C2_096 | 2 | Plus | 16,817,978 | 16,818,002 | At2g40400 | GGCCAACTTCCGATTATCTTCTCAG |
| C2_097 | 2 | Minus | 17,150,992 | 17,150,968 | At2g41250 | TTGGATCTCCTCTGCTTCGTCATCT |
| C2_098 | 2 | Minus | 17,550,929 | 17,550,905 | At2g42260 | TGCCCTCACTCCAGAGAAGAAGCTC |
| C2_099 | 2 | Minus | 18,149,870 | 18,149,846 | At2g43950 | GCACATGCATGAACATATCCTTCGT |
| C2_100 | 2 | Minus | 18,346,924 | 18,346,900 | At2g44580 | ATTATCGTCTCTGTAGCCGTCTTCA |
| C2_101 | 2 | Minus | 18,497,964 | 18,497,940 | At2g44970 | TCTTGAAGAATCATCCGCTTCCCCG |
| C2_102 | 2 | Minus | 18,600,307 | 18,600,283 | At2g45230 | CACAGATGGTGCATGGTCTAAGGAT |
| C2_103 | 2 | Minus | 18,722,122 | 18,722,098 | At2g45550 | ACGTTATATGCCATTCCCGTAAAGA |
| C2_104 | 2 | Minus | 18,861,369 | 18,861,345 | At2g45970 | TCCGTTTAAATTCGTTGCGTTCAAT |
| C2_105 | 2 | Plus | 19,099,116 | 19,099,140 | At2g46640 | AGAAGGACGTTCCATACAAGCCAAC |
| C2_106 | 2 | Minus | 19,474,812 | 19,474,788 | At2g47600 | TGCCAAAAGCTGGAGAACTGAAAAA |
| C3_001 | 3 | Minus | 192,362 | 192,338 | At3g01490 | GGCACAGGTCAGACGCAGAGATAGC |
| C3_002 | 3 | Plus | 340,169 | 340,193 | At3g02010 | AGGGTACAAGCCTGACACTAGTTCG |
| C3_003 | 3 | Minus | 603,862 | 603,838 | At3g02780 | CATTCTTGTTGATGAAACTGATCGT |
| C3_004 | 3 | Plus | 808,224 | 808,248 | At3g03380 | GTATTGACTCTGAAACAAGATCTCC |
| C3_005 | 3 | Minus | 903,318 | 903,294 | At3g03670 | TTATTTTCCCCGTGGCATTAGCGCA |
| C3_006 | 3 | Plus | 1,079,332 | 1,079,356 | At3g04110 | CGTTAATCCGGAATTTCAGGGCTCT |
| C3_007 | 3 | Minus | 1,261,198 | 1,261,174 | At3g04640 | GGATTACGTCCGATTCCGATTTATG |
| C3_008 | 3 | Plus | 1,357,789 | 1,357,813 | At3g04910 | GCCATCCGAAAAAACCGAAAATCAG |
| C3_009 | 3 | Minus | 1,387,823 | 1,387,799 | At3g05000 | CCTTCATCTGAGAATGAAACCCAAG |
| C3_010 | 3 | Plus | 1,468,110 | 1,468,134 | At3g05170 | GACTGAGGCCACTGAAGATAGAGAA |
| C3_011 | 3 | Plus | 1,512,584 | 1,512,608 | At3g05310 | ATCAACTGATGAACATTATGCTGTA |
| C3_012 | 3 | Minus | 1,751,984 | 1,751,960 | At3g05860 | AGCTTAATTTGACTGATAGAAACCG |
| C3_013 | 3 | Minus | 2,677,419 | 2,677,395 | At3g08820 | GCTTATGGGTTTTGTTCCAACGACA |
| C3_014 | 3 | Plus | 3,158,205 | 3,158,229 | At3g10200 | ATACCTATTAGATGGCCAACCAGTA |
| C3_015 | 3 | Minus | 3,391,282 | 3,391,258 | At3g10840 | CTAGGGAATGCTTTTGGAGGGTCAC |
| C3_016 | 3 | Plus | 3,449,960 | 3,449,984 | At3g11000 | TAAGTTTAAGGTCATCCATCGTTCT |
| C3_017 | 3 | Minus | 3,551,666 | 3,551,642 | At3g11325 | GCCTCGGCTTATATACTGAAAAACA |
| C3_018 | 3 | Plus | 3,561,771 | 3,561,795 | At3g11370 | GGCATCGTCTTTCTCACATTCCTTG |
| C3_019 | 3 | Plus | 3,663,424 | 3,663,448 | At3g11590 | AAGATCACCATATCCTCTCGGGTTC |
| C3_020 | 3 | Plus | 3,886,666 | 3,886,690 | At3g12190 | GAGCTACTGGTTCAACAGGAGAAGT |
| C3_021 | 3 | Plus | 4,391,374 | 4,391,398 | At3g13470 | TGGGACCACACAAGAAGCAGTGAAC |
| C3_022 | 3 | Minus | 4,686,211 | 4,686,187 | At3g14130 | TCATGCTGTGAAAGGTAGGATTCCG |
| C3_023 | 3 | Plus | 4,922,934 | 4,922,958 | At3g14650 | CTGCAGCGAGATTGTTTGCAAATGG |
| C3_024 | 3 | Minus | 5,032,046 | 5,032,022 | At3g14950 | AGCAATAGAGCTATCACCGAGTAAT |
| C3_025 | 3 | Plus | 5,439,672 | 5,439,696 | At3g16030 | TGTCAAACAGATACCCTACTGCAGG |
| C3_026 | 3 | Minus | 5,619,798 | 5,619,774 | At3g16520 | TCCGGTTCTGAATCATAAAGCAGTC |
| C3_027 | 3 | Minus | 5,701,283 | 5,701,259 | At3g16750 | CTTGGCAACAGTGATAATGACTCTA |
| C3_028 | 3 | Plus | 5,936,712 | 5,936,736 | At3g17360 | GCTGAGACCATTAGGCACAATGGAA |
| C3_029 | 3 | Minus | 6,169,555 | 6,169,531 | At3g18035 | CGTTGAGCCACAAGTTATGGAAGAA |
| C3_030 | 3 | Minus | 6,417,382 | 6,417,358 | At3g18650 | TTCAATTTGGATCAAAACGGAAACA |
| C3_031 | 3 | Plus | 6,507,038 | 6,507,062 | At3g18860 | TTTGCATCATCATGGAGCAAGTCTA |
| C3_032 | 3 | Minus | 6,755,234 | 6,755,210 | At3g19490 | ATTTTGGCAGCTCATTGCATTCTGT |
| C3_033 | 3 | Plus | 6,823,560 | 6,823,584 | At3g19650 | AGATTCAGACCCTAGACGTCACCGT |
| C3_034 | 3 | Plus | 6,905,750 | 6,905,774 | At3g19860 | TAGAAGAAAGCTCTCATTCTAGCAA |
| C3_035 | 3 | Minus | 7,120,495 | 7,120,471 | At3g20420 | TTAGTACTGAGAAACTCGCTCGTGT |
| C3_036 | 3 | Minus | 7,186,831 | 7,186,807 | At3g20570 | GACACTCCAAACAGCGCAGCTTCTT |
| C3_037 | 3 | Minus | 7,606,763 | 7,606,739 | At3g21600 | CTTACATCAGGAATCGTCAAAAATG |
| C3_038 | 3 | Plus | 7,930,774 | 7,930,798 | At3g22400 | ACCAGGTACGGATGAGTATGCTGAG |
| C3_039 | 3 | Minus | 7,999,243 | 7,999,219 | At3g22560 | CTCTCCTCCAAGACGGTCATTCGAT |
| C3_040 | 3 | Minus | 8,034,091 | 8,034,067 | At3g22740 | CGACCTAATAGCATTCGAGACTATT |
| C3_041 | 3 | Plus | 8,070,112 | 8,070,136 | At3g22810 | ATATCATGACATTAACCGCTGGAGC |
| C3_042 | 3 | Plus | 8,139,661 | 8,139,685 | At3g22960 | CTGTGACGGAAGCTGAGCTTAAGGA |
| C3_043 | 3 | Minus | 8,222,572 | 8,222,548 | At3g23110 | GTGGAGAAACCCATGTCCCCATACC |
| C3_044 | 3 | Minus | 8,222,598 | 8,222,574 | At3g23110 | CAGACTCTATGGACTCGATCAAATT |
| C3_045 | 3 | Plus | 8,344,950 | 8,344,974 | At3g23320 | GTTAATCGCCTTATTCGAAACAAAG |
| C3_046 | 3 | Plus | 8,612,178 | 8,612,202 | At3g23840 | AAGTTCCATTCACGTCGATTTCTCG |
| C3_047 | 3 | Minus | 8,832,555 | 8,832,531 | At3g24340 | CAAATTCTCTTGATGCATGGTAAAG |
| C3_048 | 3 | Minus | 9,011,746 | 9,011,722 | At3g24630 | TGTTATTACATAGTCGAAGAGCCTT |
| C3_049 | 3 | Minus | 9,168,646 | 9,168,622 | At3g25180 | CGATGAATGGCGTTATAAGGAGGCC |
| C3_050 | 3 | Minus | 9,237,479 | 9,237,455 | At3g25480 | GAAGTATAAACCAATATCCGCCATG |
| C3_051 | 3 | Minus | 9,288,765 | 9,288,741 | At3g25570 | TGAACGGATACTTTAGCCAAGAGAG |
| C3_052 | 3 | Plus | 9,309,023 | 9,309,047 | At3g25600 | GTTTTCTCATATTATGGCTAAATCG |
| C3_053 | 3 | Minus | 9,318,799 | 9,318,775 | At3g25620 | GGCATTCTCGCGTTGCTCATTTACA |
| C3_054 | 3 | Plus | 9,382,267 | 9,382,291 | At3g25720 | TTGTTGCTTGTGTTCTTGTGCACCC |
| C3_055 | 3 | Minus | 9,459,661 | 9,459,637 | At3g25850 | TCGCATTTTTCATCTTGGTCTCGGA |
| C3_056 | 3 | Minus | 9,613,121 | 9,613,097 | At3g26250 | TTTATGGGTATATCTCGACCTATTT |
| C3_057 | 3 | Plus | 9,679,032 | 9,679,056 | At3g26440 | GAAATCATCTAAGACGAGTTCACAA |
| C3_058 | 3 | Minus | 9,713,327 | 9,713,303 | At3g26510 | TCCGTTAGCGTTACCGTCATCAACC |
| C3_059 | 3 | Minus | 9,748,377 | 9,748,353 | At3g26550 | GCACCGGATGTATGCGCCGTTGTCT |
| C3_060 | 3 | Minus | 9,805,711 | 9,805,687 | At3g26690 | GCCATTTCCAGCATTTCAAATCGAG |
| C3_061 | 3 | Minus | 10,011,316 | 10,011,292 | At3g27150 | ATGGGAACTCATACCGGACATTCTC |
| C3_062 | 3 | Plus | 10,151,134 | 10,151,158 | At3g27420 | GGAAATCATTGCATAGCATCTGGCA |
| C3_063 | 3 | Minus | 10,196,030 | 10,196,006 | At3g27530 | GAGAAAGACTACATCAACCGCTTGA |
| C3_064 | 3 | Plus | 10,308,857 | 10,308,881 | At3g27810 | CGGGATCATCAGCAGAAGCAGAAGT |
| C3_065 | 3 | Plus | 10,437,496 | 10,437,520 | At3g28040 | ACTTCTCCGGTAACATTCCTGATGG |
| C3_066 | 3 | Plus | 10,526,590 | 10,526,614 | At3g28220 | AGTGTTCTCATTCACTAAGAGTTTG |
| C3_067 | 3 | Minus | 10,630,712 | 10,630,688 | At3g28390 | ATTGGCAAAGGGTCCAAAGGGAGCT |
| C3_068 | 3 | Minus | 10,638,408 | 10,638,384 | At3g28400 | CTGCATCACTGTGGATAATGCTATT |
| C3_069 | 3 | Plus | 10,839,745 | 10,839,769 | At3g28840 | AGGTCTACGAAGTGCAAGGGTCGTT |
| C3_070 | 3 | Plus | 11,071,084 | 11,071,108 | At3g29080 | TCTTTGCCTTTGTTAGTAGTAGCTG |
| C3_071 | 3 | Minus | 11,384,022 | 11,383,998 | At3g29575 | GACTTGTTCGTTACCCGTGGAGACG |
| C3_072 | 3 | Plus | 11,721,232 | 11,721,256 | At3g29800 | ATTACTTGCTGCATGGTCTACCGGG |
| C3_073 | 3 | Plus | 11,734,424 | 11,734,448 | At3g29820 | AGCGAAGGCACATTTCCAACACAGA |
| C3_074 | 3 | Minus | 11,965,858 | 11,965,834 | At3g30370 | CCTTCAGATTCAATGTGGAGGATTA |
| C3_075 | 3 | Minus | 12,477,783 | 12,477,759 | At3g30800 | ACCGATAGTTCAACCACCATACTGG |
| C3_076 | 3 | Minus | 13,382,889 | 13,382,865 | NO_GENE | TCAAGCTTCCCGAGGAGATCCGTCA |
| C3_077 | 3 | Minus | 15,284,751 | 15,284,727 | At3g43320 | GGAAGTCTCATTATTATCGAATCCT |
| C3_078 | 3 | Minus | 15,783,158 | 15,783,134 | At3g43960 | CATTTGTTGTCTCCAAGTGTGTTTA |
| C3_079 | 3 | Minus | 15,961,631 | 15,961,607 | At3g44260 | TCGTCGGCAAGGTATCGATTTTGAG |
| C3_080 | 3 | Minus | 16,169,512 | 16,169,488 | At3g44580 | TGTCTCCTGCGGTTGCTGATGACTT |
| C3_081 | 3 | Plus | 16,450,571 | 16,450,595 | At3g44980 | CAGACATCTGTAATATAGCAAGAGT |
| C3_082 | 3 | Plus | 16,629,387 | 16,629,411 | At3g45290 | CCCTCTCGTTGAGTCAAACGACACG |
| C3_083 | 3 | Plus | 16,661,317 | 16,661,341 | At3g45400 | ATTGGCTTACGTTGGATTTGCTTGC |
| C3_084 | 3 | Minus | 17,033,399 | 17,033,375 | At3g46330 | AGAATCCCGAAAAATCTTGAATCGG |
| C3_085 | 3 | Minus | 17,140,137 | 17,140,113 | At3g46530 | AAGAGTTCATGTGAGCTGGGCTTCC |
| C3_086 | 3 | Minus | 17,338,527 | 17,338,503 | At3g47050 | CACATTGCTCCCTATAAGAAGTGTA |
| C3_087 | 3 | Minus | 17,586,556 | 17,586,532 | At3g47680 | AGATTATCAAGGACCAAATTGCTGG |
| C3_088 | 3 | Plus | 17,656,128 | 17,656,152 | At3g47830 | ACACTACTTAGACCTACCGCTGAAG |
| C3_089 | 3 | Minus | 17,765,927 | 17,765,903 | At3g48090 | CCGTCCAAGAGAGTGAACAGAGAAT |
| C3_090 | 3 | Plus | 17,914,863 | 17,914,887 | At3g48350 | CCCACGAGGCAATAAAGCGGTTCAA |
| C3_091 | 3 | Plus | 18,034,414 | 18,034,438 | At3g48650 | TACAAGGCTATCTACGACGAGCATC |
| C3_092 | 3 | Minus | 18,046,578 | 18,046,554 | At3g48690 | TCCTGGTTTGAACGATACAGGAATC |
| C3_093 | 3 | Plus | 18,117,461 | 18,117,485 | At3g48830 | TGGCTAGCTGCGAAAATATTGCACA |
| C3_094 | 3 | Plus | 18,228,910 | 18,228,934 | At3g49150 | TGACCAAAAAGCAGGATGGGAGTCA |
| C3_095 | 3 | Plus | 18,300,768 | 18,300,792 | At3g49330 | GTTGGTGGATGCTCATTTAGACAAC |
| C3_096 | 3 | Minus | 18,358,419 | 18,358,395 | At3g49500 | CATGAAAATCTCAGCGAGGAGGAAA |
| C3_097 | 3 | Plus | 18,513,480 | 18,513,504 | At3g49910 | TCAGGTTTACCGTCGCAAATGGGTG |
| C3_098 | 3 | Minus | 18,539,845 | 18,539,821 | At3g49980 | CTTGTTCTGATTACCGCGTGTCTTT |
| C3_099 | 3 | Plus | 18,723,590 | 18,723,614 | At3g50430 | AGTCTAGATCAATGTTTCTTCGGTT |
| C3_100 | 3 | Minus | 18,901,192 | 18,901,168 | At3g50820 | CATCTCCGATCAAACCAAACCGTGG |
| C3_101 | 3 | Plus | 19,046,212 | 19,046,236 | At3g51280 | ATCAGCCCCTTGTTCTCCTGCAAAG |
| C3_102 | 3 | Minus | 19,478,696 | 19,478,672 | At3g52510 | TTCAGCTCAGACTGGGAAAGAAACA |
| C3_103 | 3 | Plus | 19,537,286 | 19,537,310 | At3g52680 | ACACGTGAAGCAGAGTGGTGGAATC |
| C3_104 | 3 | Minus | 19,626,786 | 19,626,762 | At3g52910 | TATTAAGAAAAGTCTCCTCCATCAA |
| C3_105 | 3 | Minus | 20,023,064 | 20,023,040 | At3g54040 | GCCCTGTCTCCGAAATTGCACCAGG |
| C3_106 | 3 | Minus | 20,439,359 | 20,439,335 | At3g55120 | GGTTCTGGAATCTATCATCGGGAAG |
| C3_107 | 3 | Minus | 21,059,962 | 21,059,938 | At3g56860 | ACAGGCCTTGACAGCTTTATTGGCA |
| C3_108 | 3 | Plus | 21,738,436 | 21,738,460 | At3g58760 | TGTCCTAACTGATAAAGAAGTTCCA |
| C3_109 | 3 | Minus | 21,834,034 | 21,834,010 | At3g59050 | ATCAGTGCACGGTGCTTATTCAACC |
| C3_110 | 3 | Plus | 21,954,682 | 21,954,706 | At3g59380 | TCCTGTAAGAGCTAACTATTGGGCA |
| C3_111 | 3 | Minus | 22,330,704 | 22,330,680 | At3g60390 | AGATGACAGTTCGAGGAAGAAACTC |
| C3_112 | 3 | Minus | 22,530,710 | 22,530,686 | At3g60940 | GTCCCGAAAGTTCTACTTCCACTGC |
| C3_113 | 3 | Plus | 22,632,878 | 22,632,902 | At3g61130 | ATTTGTTACAAGAACTCCAGGCCCG |
| C3_114 | 3 | Plus | 22,725,443 | 22,725,467 | At3g61390 | GATATCCCTGAATCAATGGAAATCA |
| C3_115 | 3 | Plus | 22,947,197 | 22,947,221 | At3g61940 | CGAGGCAGCTCGAAAAGGGATTGAT |
| C3_116 | 3 | Minus | 22,965,532 | 22,965,508 | At3g61980 | GTGGATGCACAATCAACTGTTTCCG |
| C3_117 | 3 | Plus | 22,989,194 | 22,989,218 | At3g62060 | CTGCGATATCTTTTCTGGAGTATAA |
| C3_118 | 3 | Minus | 23,023,725 | 23,023,701 | At3g62160 | CGCTCCCGATTGGTTTCTACGGCAA |
| C3_119 | 3 | Minus | 23,086,877 | 23,086,853 | At3g62360 | GTAGTCTAAGATCTGTCACAGACAA |
| C3_120 | 3 | Plus | 23,344,414 | 23,344,438 | At3g63170 | ATAAGGCTACAGATTGTCTACGGCA |
| C4_001 | 4 | Minus | 124,134 | 124,110 | At4g00290 | CGAACATACGCCTGCATAATGATGC |
| C4_002 | 4 | Minus | 216,954 | 216,930 | At4g00480 | GAGCAGCAAGTCTCGGGTTTTGTTC |
| C4_003 | 4 | Minus | 266,179 | 266,155 | At4g00640 | ATGAAGAGTCCGTTGCTAAAGAAGC |
| C4_004 | 4 | Minus | 423,549 | 423,525 | At4g00980 | CTATGGAGATTGTCGCTACACAAAA |
| C4_005 | 4 | Minus | 515,956 | 515,932 | At4g01230 | GGATTTGGTGGTCGTCGTCTTCTCA |
| C4_006 | 4 | Plus | 695,872 | 695,896 | At4g01610 | CCTTCAAGCAAGAATGTGTTTAGGG |
| C4_007 | 4 | Minus | 703,957 | 703,933 | At4g01650 | AGCTTGAGGTTCTGCCACATGGGAG |
| C4_008 | 4 | Minus | 823,892 | 823,868 | At4g01910 | AAGCACGATAGATCTTAGCGAATTA |
| C4_009 | 4 | Plus | 1,038,056 | 1,038,080 | At4g02350 | CATTCATCAGACACTATCTCTTGGG |
| C4_010 | 4 | Plus | 1,188,732 | 1,188,756 | At4g02700 | TGGCCTCGCTGTTGACGGCTGCGAT |
| C4_011 | 4 | Plus | 1,439,540 | 1,439,564 | At4g03280 | AGAATTTCTTGTCAAGCGTCGAGTA |
| C4_012 | 4 | Minus | 1,546,311 | 1,546,287 | At4g03480 | TGATGCATCAACACGCCAAATGGAG |
| C4_013 | 4 | Plus | 2,142,434 | 2,142,458 | At4g04390 | TCGATTGACATTTGAGATGACCATG |
| C4_014 | 4 | Plus | 3,557,989 | 3,558,013 | At4g07770 | GATTTGGTGCAGTGGCCATTCCTCC |
| C4_015 | 4 | Plus | 4,058,456 | 4,058,480 | At4g08130 | ACTAGAACACCAACGAGGCAATATA |
| C4_016 | 4 | Plus | 4,335,175 | 4,335,199 | At4g08450 | CGGTATACGACTCTCTGAGGATGAT |
| C4_017 | 4 | Minus | 4,353,546 | 4,353,522 | At4g08480 | TAAACGACATTAAGTCCCGCAAGGA |
| C4_018 | 4 | Minus | 4,429,733 | 4,429,709 | At4g08590 | CTTGCATTGCTGGACAAGAAGACTA |
| C4_019 | 4 | Minus | 4,581,179 | 4,581,155 | At4g08810 | AAGCATCGAAGTATGTTGAGAGGCC |
| C4_020 | 4 | Plus | 4,694,249 | 4,694,273 | At4g08930 | GCTACAAATGGCTGCAGATATGGTT |
| C4_021 | 4 | Minus | 4,742,864 | 4,742,840 | At4g09010 | AGGAGATTGACTCTATCTCGAAGGG |
| C4_022 | 4 | Plus | 4,906,414 | 4,906,438 | At4g09360 | TCCCTGATGAATTGGTAATGGTGCC |
| C4_023 | 4 | Plus | 4,937,229 | 4,937,253 | At4g09430 | AGCTTACGGGCAAAGTCTCTCCTGG |
| C4_024 | 4 | Minus | 5,037,642 | 5,037,618 | At4g09600 | AGCAAATCGTCGAGGCCAAATCTGT |
| C4_025 | 4 | Plus | 5,089,939 | 5,089,963 | At4g09690 | GCTCCTCACGGGTCTATTACCGATC |
| C4_026 | 4 | Minus | 5,124,137 | 5,124,113 | At4g09780 | GCACAACCATTTACTGCAATTATGG |
| C4_027 | 4 | Plus | 5,193,273 | 5,193,297 | At4g09930 | GCTGCTCAGGCAACGGGAAAAGTGC |
| C4_028 | 4 | Minus | 5,237,149 | 5,237,125 | At4g10030 | GTATGATCTTGTTCAAGGAGCACTT |
| C4_029 | 4 | Plus | 5,280,397 | 5,280,421 | At4g10120 | GTTGTTTTCAATATGCTCGTGGGAC |
| C4_030 | 4 | Plus | 5,435,879 | 5,435,903 | At4g10460 | ACTTGGCATGGAGATCTCAAGAGAC |
| C4_031 | 4 | Minus | 5,607,038 | 5,607,014 | At4g10790 | CTTCTGCTGTTGGTGAAGCGATGGA |
| C4_032 | 4 | Minus | 5,726,789 | 5,726,765 | At4g11080 | AACAGAATCCTGAAGCTGATTTCAA |
| C4_033 | 4 | Plus | 5,770,858 | 5,770,882 | At4g11160 | CTGGTGAAGCCGAGGTACTTAGCAT |
| C4_034 | 4 | Minus | 5,792,379 | 5,792,355 | At4g11200 | CAGTGTCATCCAAGAGGAAGGTGTG |
| C4_035 | 4 | Plus | 5,823,192 | 5,823,216 | At4g11270 | TGCATGTATTCACATATACGACATG |
| C4_036 | 4 | Plus | 5,929,212 | 5,929,236 | At4g11460 | GGATTGTATCATGGACGTCACAAGA |
| C4_037 | 4 | Plus | 5,931,485 | 5,931,509 | At4g11460 | CTCCTCGGTAAAACTCTTGCTTCTG |
| C4_038 | 4 | Minus | 6,060,128 | 6,060,104 | At4g11800 | CTGATATGCGACGTGTTGAAATACA |
| C4_039 | 4 | Plus | 6,241,573 | 6,241,597 | At4g12180 | ATATCGGGACACTGCAAAAGCCTTT |
| C4_040 | 4 | Plus | 6,301,134 | 6,301,158 | At4g12390 | GCAGCAGCATCAACTTCATTGTCTC |
| C4_041 | 4 | Minus | 6,447,403 | 6,447,379 | At4g12700 | TGCAACAAATGAACCAGAGTTATCT |
| C4_042 | 4 | Minus | 6,467,515 | 6,467,491 | At4g12750 | GGCACTTCTTCTGTCGGTCAACAGA |
| C4_043 | 4 | Plus | 6,497,594 | 6,497,618 | At4g12830 | AAGAGTTCTGCAAGAGTTCGGGACA |
| C4_044 | 4 | Minus | 6,584,690 | 6,584,666 | At4g13060 | TTGTTTTACCTAGTTTGCCTCAAAG |
| C4_045 | 4 | Minus | 6,621,999 | 6,621,975 | At4g13180 | ACGGTGAAAATGCTTGCAGGGGCTT |
| C4_046 | 4 | Minus | 6,780,074 | 6,780,050 | At4g13450 | GTAACTTCTTGGAACAAATGAAACG |
| C4_047 | 4 | Plus | 6,834,897 | 6,834,921 | At4g13540 | GAGAAACCATTGAGACACTGAAAGG |
| C4_048 | 4 | Plus | 6,885,275 | 6,885,299 | At4g13610 | GCTAAGGACCACAACGGTTATAAGG |
| C4_049 | 4 | Minus | 6,899,286 | 6,899,262 | At4g13630 | ATGCCCTGAGTGATATAGAACTGAA |
| C4_050 | 4 | Minus | 6,973,309 | 6,973,285 | At4g13820 | AAACCGCTTCAATGGAGTCCTTCGA |
| C4_051 | 4 | Minus | 7,065,918 | 7,065,894 | At4g14040 | TGATGACACGTGGAGCCATGAGGCA |
| C4_052 | 4 | Minus | 7,202,396 | 7,202,372 | At4g14310 | ACAGCACACCATTTCCTCTTCAGGG |
| C4_053 | 4 | Plus | 7,282,782 | 7,282,806 | At4g14460 | TCAAGGACTGATGTATTCTGCAGAT |
| C4_054 | 4 | Plus | 7,458,626 | 7,458,650 | At4g14780 | TAGCTTTGTCGATGTTTCTTCCGCG |
| C4_055 | 4 | Minus | 7,708,982 | 7,708,958 | At4g15320 | TTCGGCTTTGGCAGAGACTTGTGGA |
| C4_056 | 4 | Plus | 7,789,787 | 7,789,811 | At4g15420 | GTCCAAGAAAGCATTGATGGAGCTA |
| C4_057 | 4 | Minus | 7,821,812 | 7,821,788 | At4g15500 | TCAGCCGTGGAGCTTCCGATGAGAG |
| C4_058 | 4 | Minus | 7,867,706 | 7,867,682 | At4g15590 | GAGTTCAATTCTTTTACCAGCTTCG |
| C4_059 | 4 | Minus | 8,023,223 | 8,023,199 | At4g15980 | TGCACGTAGACCTAGAAATAATGGA |
| C4_060 | 4 | Plus | 8,055,753 | 8,055,777 | At4g16045 | ACACTTGTAAGAGTGGTCGCAAAAG |
| C4_061 | 4 | Minus | 8,134,280 | 8,134,256 | At4g16180 | GATCCTCAAAAACTAGAAGAGTTCC |
| C4_062 | 4 | Plus | 8,433,247 | 8,433,271 | At4g16820 | CGCCGATAACGATAAACAGGGGCAA |
| C4_063 | 4 | Minus | 8,717,695 | 8,717,671 | At4g17490 | GCCTGTACAACAAACGGAAGAGAGA |
| C4_064 | 4 | Plus | 8,893,737 | 8,893,761 | At4g17870 | AGGTAACTCGGAGGATGATACTCGT |
| C4_065 | 4 | Minus | 8,923,821 | 8,923,797 | At4g17910 | TGAATCTCGTGACTTGTAACTGTTT |
| C4_066 | 4 | Minus | 9,575,164 | 9,575,140 | At4g19460 | GGTACGCAAGCATAAAGAGGACTAT |
| C4_067 | 4 | Plus | 9,949,963 | 9,949,987 | At4g20340 | AAGCGGAATGCCAAGGAGCTTCGAA |
| C4_068 | 4 | Minus | 10,364,671 | 10,364,647 | At4g21400 | CATCTTCGGTAATTGTTTTCCGCCG |
| C4_069 | 4 | Plus | 10,450,933 | 10,450,957 | At4g21600 | GCATACTGGTTTTCTTGGAGATCTA |
| C4_070 | 4 | Plus | 10,538,393 | 10,538,417 | At4g21800 | GGTGGAATTCTCACTTCACTCAACT |
| C4_071 | 4 | Plus | 10,547,746 | 10,547,770 | At4g21820 | CTTGTGGCAGCTGGAGCTATAGAAA |
| C4_072 | 4 | Plus | 10,605,558 | 10,605,582 | At4g21940 | GAAGCACGTTCTAACACAACCATTG |
| C4_073 | 4 | Plus | 10,629,652 | 10,629,676 | At4g22010 | GTTCTCATCAATGGTCGCGGCTGGG |
| C4_074 | 4 | Minus | 10,749,651 | 10,749,627 | At4g22290 | GCTTGTGGGAGCTTTTCTTACTGTG |
| C4_075 | 4 | Plus | 10,902,371 | 10,902,395 | At4g22720 | GTGGTGGTAACACTCAGGTGATTGC |
| C4_076 | 4 | Minus | 11,041,950 | 11,041,926 | At4g23040 | AGAACAAATTATTCGAGCTGCTATT |
| C4_077 | 4 | Minus | 11,084,556 | 11,084,532 | At4g23130 | TACAAGACCCTACTTACGTTGGCCA |
| C4_078 | 4 | Minus | 11,124,387 | 11,124,363 | At4g23230 | TCGACCCTTGCATACGATGGAGCAT |
| C4_079 | 4 | Plus | 11,429,615 | 11,429,639 | At4g24000 | CTCCACTCAAGGATATAATGTCACG |
| C4_080 | 4 | Plus | 11,559,403 | 11,559,427 | At4g24290 | CTATTGGCTGTGGTTATGATCTAGC |
| C4_081 | 4 | Minus | 11,588,364 | 11,588,340 | At4g24410 | TGATCTATCGATGATAAACGCCATA |
| C4_082 | 4 | Minus | 11,785,134 | 11,785,110 | At4g24920 | TCTTAGAGACTTTGCCAAGGACAGC |
| C4_083 | 4 | Minus | 11,868,195 | 11,868,171 | At4g25160 | GGCAAGGAACTCATTTTCCGGCGTA |
| C4_084 | 4 | Plus | 11,946,130 | 11,946,154 | At4g25400 | CATAGACTATTTGCAAAGGAATATC |
| C4_085 | 4 | Plus | 12,313,215 | 12,313,239 | At4g26410 | CGCTTTTGAGACAGAAGAGGATTGC |
| C4_086 | 4 | Minus | 12,657,061 | 12,657,037 | At4g27350 | ATCTCTGGTGGTAACAATTCCGCCG |
| C4_087 | 4 | Plus | 12,848,846 | 12,848,870 | At4g27880 | CCCTTTTCTTGTAGCCCATCTCAGG |
| C4_088 | 4 | Plus | 13,243,125 | 13,243,149 | At4g28950 | GCCAATGTAGCTGTGGATGGACAAA |
| C4_089 | 4 | Plus | 13,475,201 | 13,475,225 | At4g29580 | AATTTGTAACCATCTAAGCTGCAGG |
| C4_090 | 4 | Minus | 13,631,330 | 13,631,306 | At4g29990 | GAGAGTCCATCACACAAATTTGACA |
| C4_091 | 4 | Plus | 14,081,620 | 14,081,644 | At4g31060 | GGAGTTTACAATTTTCCCACTGATA |
| C4_092 | 4 | Minus | 14,244,307 | 14,244,283 | At4g31520 | TACGAGAAATGTTCCTTAGGATTCC |
| C4_093 | 4 | Plus | 14,297,249 | 14,297,273 | At4g31650 | CACGTAGATCGTTTTACAGTAGGGG |
| C4_094 | 4 | Minus | 14,484,134 | 14,484,110 | At4g32120 | GGTAGGTTTTCCAAGTCTCAGAACA |
| C4_095 | 4 | Minus | 14,853,537 | 14,853,513 | At4g32920 | CCACGTGAGGCATCAAGAGTGTGGA |
| C4_096 | 4 | Minus | 15,218,880 | 15,218,856 | At4g33905 | CTACTTCCCACAATGCTTAATGGTG |
| C4_097 | 4 | Plus | 15,581,802 | 15,581,826 | At4g34880 | CCATATGGGATTAGCTTTGGAGGAC |
| C4_098 | 4 | Minus | 15,666,401 | 15,666,377 | At4g35090 | TTGCCTCTTCAACCTGTTGGACGTA |
| C4_099 | 4 | Minus | 15,871,342 | 15,871,318 | At4g35640 | ACTGCAGGCGTATAGAGTAGCACAT |
| C4_100 | 4 | Plus | 15,897,458 | 15,897,482 | At4g35730 | GACACAGCTACAACCCTCGATCAGT |
| C4_101 | 4 | Plus | 15,969,642 | 15,969,666 | At4g35900 | GGTCACTGTTCTTTACAGCTCTCCT |
| C4_102 | 4 | Minus | 16,063,531 | 16,063,507 | At4g36140 | GTCCCGAGAGTTAGAAGTGTGAAAC |
| C4_103 | 4 | Minus | 16,169,467 | 16,169,443 | At4g36430 | GACATTGGAACAATCCTTCGCTGCT |
| C4_104 | 4 | Minus | 16,175,757 | 16,175,733 | At4g36450 | CAATCAACAGCGAAACCAATGAGAG |
| C4_105 | 4 | Minus | 16,249,796 | 16,249,772 | At4g36650 | GAACCTACCGGATTCATTACCGCCT |
| C4_106 | 4 | Minus | 16,525,032 | 16,525,008 | At4g37320 | GTCCTGCAAAAACTGGTTGATGAGA |
| C4_107 | 4 | Plus | 16,682,901 | 16,682,925 | At4g37710 | CAGTAAACTTCAAAGTGTTGGTTCA |
| C4_108 | 4 | Plus | 16,870,459 | 16,870,483 | At4g38170 | GCTTCAACAGTCTACGCCAAGAAGC |
| C4_109 | 4 | Minus | 16,902,435 | 16,902,411 | At4g38260 | GCTTTCCTCACCAATTTCAAGGAAG |
| C4_110 | 4 | Minus | 17,045,685 | 17,045,661 | At4g38730 | CAACATTCTTGTGTATATCGGAATT |
| C4_111 | 4 | Plus | 17,369,987 | 17,370,011 | At4g39650 | CATCGCCGGTACAACACAAGTTTAC |
| C5_001 | 5 | Plus | 53,017 | 53,041 | At5g01150 | ACATGTGTAGAACCTTCAGGGACTT |
| C5_002 | 5 | Plus | 311,449 | 311,473 | At5g01810 | GAGGTTTACATCAAACAGAGAGGCA |
| C5_003 | 5 | Minus | 369,372 | 369,348 | At5g01950 | GGAATAGAGGCGACCCTTGCAGATC |
| C5_004 | 5 | Minus | 420,763 | 420,739 | At5g02130 | ACGCCACTGGAAATTTTGAACTGGC |
| C5_005 | 5 | Plus | 668,807 | 668,831 | At5g02880 | GCCATAACGGAAGGTCAAGGTTCCT |
| C5_006 | 5 | Plus | 727,318 | 727,342 | At5g03100 | CAATTGCCAAAATTCTCACTGGTTG |
| C5_007 | 5 | Minus | 812,351 | 812,327 | At5g03340 | CAGTTGAAGGCGTTACTGGAAACCT |
| C5_008 | 5 | Minus | 1,016,618 | 1,016,594 | At5g03820 | CAAACATTGTTGGAAGCGAAAGAGC |
| C5_009 | 5 | Minus | 1,213,089 | 1,213,065 | At5g04330 | AAGGACACTGAGATTTCCGGTTACT |
| C5_010 | 5 | Minus | 1,357,828 | 1,357,804 | At5g04710 | ACACAAGGGTCTTGTAATCAAACAT |
| C5_011 | 5 | Plus | 1,399,949 | 1,399,973 | At5g04820 | CTGAGTCCTTAGAGATGGTGGTTCG |
| C5_012 | 5 | Minus | 1,468,667 | 1,468,643 | At5g04980 | CGAGTCACGTTTCTCCGATCATCGG |
| C5_013 | 5 | Minus | 1,499,818 | 1,499,794 | At5g05080 | TTGCCAAGGAGCTGAAGAGTCTTGA |
| C5_014 | 5 | Plus | 1,629,887 | 1,629,911 | At5g05500 | GATCATGGTCATTGGCCGGAGCAGA |
| C5_015 | 5 | Plus | 1,768,084 | 1,768,108 | At5g05870 | GTCCTCTGTGCTTATAAGTTCTCTT |
| C5_016 | 5 | Plus | 2,206,679 | 2,206,703 | At5g07110 | GATCTGGACTTTTCGCATTCTTCAA |
| C5_017 | 5 | Minus | 2,516,073 | 2,516,049 | At5g07880 | TTCACCTAAGAGAAAAGTTATCGAC |
| C5_018 | 5 | Minus | 2,782,348 | 2,782,324 | At5g08580 | CCTGCTGATGGGTTTATGACGGAGA |
| C5_019 | 5 | Minus | 3,008,152 | 3,008,128 | At5g09710 | TTAGTCGTTGGAATTCTAAGCACGA |
| C5_020 | 5 | Plus | 3,200,537 | 3,200,561 | At5g10190 | TCCACGGCCTTTAGCCACGGTCTGG |
| C5_021 | 5 | Plus | 3,354,970 | 3,354,994 | At5g10610 | ATTGCCGAAGCCATGGTCTGAAATG |
| C5_022 | 5 | Plus | 3,439,814 | 3,439,838 | At5g10910 | ACACGGTCGAGCAGAGGGTATGAAC |
| C5_023 | 5 | Minus | 3,713,569 | 3,713,545 | At5g11560 | GCACCAAAGGTACATTGGGAAAGTG |
| C5_024 | 5 | Minus | 4,084,360 | 4,084,336 | At5g12930 | AATTTCGTAGATTGTGGCTGCTGGC |
| C5_025 | 5 | Minus | 4,358,667 | 4,358,643 | At5g13550 | TCTCTAATGCTTTGGGTGGAATCGC |
| C5_026 | 5 | Minus | 4,719,907 | 4,719,883 | At5g14640 | ATTCCCGCAAATAAAGGCTCATCCT |
| C5_027 | 5 | Plus | 4,795,652 | 4,795,676 | At5g14830 | CGGATTTTCATTGCTGATGGGGACT |
| C5_028 | 5 | Minus | 4,834,268 | 4,834,244 | At5g14940 | CTTCTCATAGTGATTGCTGGGATAG |
| C5_029 | 5 | Minus | 4,968,678 | 4,968,654 | At5g15300 | CGAACGCTATTAGGTGCATGTAAGA |
| C5_030 | 5 | Plus | 5,034,564 | 5,034,588 | At5g15510 | AGCTTTGGAGGAATGCTGTAATCTC |
| C5_031 | 5 | Minus | 5,189,567 | 5,189,543 | At5g15900 | AGACAAAGGATTCTTGGCGCAAGTT |
| C5_032 | 5 | Minus | 5,293,726 | 5,293,702 | At5g16210 | AGCTTGCAGAATATGTCCGCCCTGA |
| C5_033 | 5 | Minus | 5,910,781 | 5,910,757 | At5g17880 | CCAATCTTCTACAAGGTACAGCCCG |
| C5_034 | 5 | Plus | 6,372,560 | 6,372,584 | At5g19060 | ACCTCCGGCGAAAGTGCGGTGGATA |
| C5_035 | 5 | Minus | 6,543,334 | 6,543,310 | At5g19410 | CGAGATGGATCAACGTCGGGATCAT |
| C5_036 | 5 | Minus | 6,589,047 | 6,589,023 | At5g19530 | GGAGATTCACAAAGCCACATCACCC |
| C5_037 | 5 | Minus | 6,850,241 | 6,850,217 | At5g20290 | GCTCCGTTCAAGCAGTGGTACCTCT |
| C5_038 | 5 | Plus | 7,029,239 | 7,029,263 | At5g20750 | CTATGCGATGGACACATATGCCACC |
| C5_039 | 5 | Minus | 7,075,280 | 7,075,256 | At5g20860 | TAGCTCTTACTTAGGACGCCCGTGG |
| C5_040 | 5 | Plus | 7,182,012 | 7,182,036 | At5g21120 | AGCTCCACAAAGAGGCAACGATGAC |
| C5_041 | 5 | Plus | 7,194,061 | 7,194,085 | At5g21150 | CAAAGCCGCCTCTGTTCCTTCTTTG |
| C5_042 | 5 | Plus | 7,205,733 | 7,205,757 | At5g21170 | CCATCTGGCATATACCACTACAAGG |
| C5_043 | 5 | Minus | 7,376,110 | 7,376,086 | At5g22350 | GTTTTTAGCTTGTTAGGTCTCAGTC |
| C5_044 | 5 | Plus | 7,422,947 | 7,422,971 | At5g22470 | AGGGAAACTTCCAGAATCTGAACAG |
| C5_045 | 5 | Minus | 7,452,049 | 7,452,025 | At5g22510 | TTCCTACTTTGTTAGTTACTGATGG |
| C5_046 | 5 | Plus | 7,784,285 | 7,784,309 | At5g23200 | AAACCGCAGAAAACGGGTCAGACAG |
| C5_047 | 5 | Minus | 7,909,425 | 7,909,401 | At5g23530 | CGAACAGATTGGTGCTGGAAAGCGA |
| C5_048 | 5 | Plus | 7,963,792 | 7,963,816 | At5g23690 | GAAACAACTGAATTCGAAAGATCTT |
| C5_049 | 5 | Minus | 7,999,671 | 7,999,647 | At5g23800 | GCAAAACTGTGGACTTGCGTAGTCT |
| C5_050 | 5 | Plus | 8,019,833 | 8,019,857 | At5g23860 | CCACGCCACGGCAGGTACTTGACAG |
| C5_051 | 5 | Plus | 8,034,751 | 8,034,775 | At5g23890 | ATGATTCCGATAACGATGACAAGAA |
| C5_052 | 5 | Minus | 8,074,372 | 8,074,348 | At5g23980 | CCAGTGTAGTTTTCTTATGGCGCAA |
| C5_053 | 5 | Minus | 8,333,208 | 8,333,184 | At5g24470 | AACTCGAACGGAAAGAGAGACGATC |
| C5_054 | 5 | Plus | 8,592,385 | 8,592,409 | At5g25010 | AGTATCTCGGCGATTTATTCGGCAC |
| C5_055 | 5 | Minus | 8,612,970 | 8,612,946 | At5g25060 | CCTCAGACATGTCATTGATACATTG |
| C5_056 | 5 | Plus | 8,725,696 | 8,725,720 | At5g25250 | TCTTCCCGCTGTTTTTACGATTGGT |
| C5_057 | 5 | Minus | 8,782,435 | 8,782,411 | At5g25370 | GGACCTCAAGTTAGGGGAATTGCTG |
| C5_058 | 5 | Plus | 8,809,026 | 8,809,050 | At5g25410 | TGAATTCCAACTATGAACAACGTCG |
| C5_059 | 5 | Plus | 8,812,669 | 8,812,693 | At5g25415 | GTTCAGGTTCAAGTCCCCCGATGGG |
| C5_060 | 5 | Minus | 8,842,971 | 8,842,947 | At5g25470 | GCCTAGGAATGCTACTGATAAGATA |
| C5_061 | 5 | Plus | 9,013,565 | 9,013,589 | At5g25900 | TTCGCTACGCCCACGAGGATACGCA |
| C5_062 | 5 | Minus | 9,021,381 | 9,021,357 | At5g25920 | ATAAGGAACCGAAACTACTGCCAAG |
| C5_063 | 5 | Minus | 9,114,758 | 9,114,734 | At5g26150 | GATCTCTCTTCTCAAAATAGTATGG |
| C5_064 | 5 | Plus | 9,205,091 | 9,205,115 | At5g26300 | TGGTTTTCATATGTCTTGCTTTACT |
| C5_065 | 5 | Minus | 9,323,009 | 9,322,985 | At5g26670 | CCTTCAGTGGAGATAGTCAGAACAA |
| C5_066 | 5 | Plus | 9,329,143 | 9,329,167 | At5g26680 | TAATAACAGACGAAGAGCAGCTTGA |
| C5_067 | 5 | Plus | 9,380,420 | 9,380,444 | At5g26790 | TGATTCAAAAGGAGGCGCGTGATGC |
| C5_068 | 5 | Minus | 9,486,962 | 9,486,938 | At5g27080 | TCATTACTATGGAGCCAAAGGGAAA |
| C5_069 | 5 | Plus | 9,507,459 | 9,507,483 | At5g27140 | GGTTAAGACCAACAAATATGTTCAC |
| C5_070 | 5 | Plus | 9,608,214 | 9,608,238 | At5g27350 | GTGGGAATTTCATCACTTGGAGGAC |
| C5_071 | 5 | Plus | 9,731,166 | 9,731,190 | At5g27620 | CATTGTTTCTCAACCGAACTCTGAG |
| C5_072 | 5 | Plus | 9,813,043 | 9,813,067 | At5g27810 | TGCTTAAATCAGAATCACAATCAGA |
| C5_073 | 5 | Plus | 9,861,541 | 9,861,565 | At5g27890 | ACGTCGATACGTTCGGAGAAGATAG |
| C5_074 | 5 | Minus | 9,915,224 | 9,915,200 | At5g27930 | CGAGGATACTCCATGGATGATATGT |
| C5_075 | 5 | Plus | 9,967,260 | 9,967,284 | At5g27970 | CCACTATGGGACCAAGTGGTTGGCC |
| C5_076 | 5 | Minus | 9,994,850 | 9,994,826 | At5g28040 | TCGCCCATGATTAAGGAGATGATGA |
| C5_077 | 5 | Minus | 10,149,904 | 10,149,880 | At5g28220 | CCAGCTAGAGATTCAAGTCGATAAT |
| C5_078 | 5 | Plus | 10,218,141 | 10,218,165 | At5g28280 | CACAAGACGGCCAAGGGAAGGAAGA |
| C5_079 | 5 | Minus | 10,536,569 | 10,536,545 | At5g28590 | GTACAGAATCTTTGTTGGGCTACCA |
| C5_080 | 5 | Minus | 12,310,942 | 12,310,918 | At5g33260 | CAGTCAACACTCCTTCTAATGTTGG |
| C5_081 | 5 | Plus | 13,052,728 | 13,052,752 | At5g35070 | TGTGACAAGAGTTACGTCGGGAGCT |
| C5_082 | 5 | Minus | 13,204,671 | 13,204,647 | At5g35230 | TAAGAAGATAACTCCAGCCAGCTCG |
| C5_083 | 5 | Plus | 13,417,249 | 13,417,273 | At5g35520 | GCCCGTAATCCCTCCAGATCAGAAA |
| C5_084 | 5 | Minus | 13,707,681 | 13,707,657 | At5g35810 | CGGTTGCCCTCCTATCATCTGTGAC |
| C5_085 | 5 | Plus | 13,806,495 | 13,806,519 | At5g35940 | TCACATCATTGGAGGGTTGCCACGA |
| C5_086 | 5 | Minus | 13,969,806 | 13,969,782 | At5g36220 | GTTGCCAATAATAGGCCATCTCCGC |
| C5_087 | 5 | Plus | 14,235,035 | 14,235,059 | At5g36870 | GCCTAAAAACCCAACATATAGTGGT |
| C5_088 | 5 | Minus | 14,256,142 | 14,256,118 | At5g36890 | AAATCATCTGCTTACTGGTTCATGA |
| C5_089 | 5 | Minus | 14,284,418 | 14,284,394 | At5g36930 | AACTATCGAACAGAAAGATGTATTC |
| C5_090 | 5 | Plus | 14,442,246 | 14,442,270 | At5g37200 | AGTCATGAAGACATCATTCGGGTGC |
| C5_091 | 5 | Plus | 14,498,789 | 14,498,813 | At5g37330 | GAAGCAGAAAGTCTCTCGTGAGAAA |
| C5_092 | 5 | Plus | 14,618,018 | 14,618,042 | At5g37520 | AGACATTCTCGGATAACAATCATAG |
| C5_093 | 5 | Minus | 14,656,821 | 14,656,797 | At5g37620 | GCAATTTCAGATACACTTGAGTACA |
| C5_094 | 5 | Minus | 14,816,374 | 14,816,350 | At5g37930 | TATTGCACCTTTGACACCGGGAGTG |
| C5_095 | 5 | Minus | 14,998,162 | 14,998,138 | At5g38260 | GATGACACATTCTGCCCTAAAGTCT |
| C5_096 | 5 | Plus | 15,174,222 | 15,174,246 | At5g38600 | ACGATGCTGACTTCCGGGAGAGAGA |
| C5_097 | 5 | Plus | 15,420,149 | 15,420,173 | At5g39220 | GTTCAATTACACGACAAAAGTCCCG |
| C5_098 | 5 | Minus | 15,479,122 | 15,479,098 | At5g39390 | TCCGGTTCGATCTCACCTTCTATTG |
| C5_099 | 5 | Minus | 15,652,226 | 15,652,202 | At5g39810 | CTCAGGCTGTTCCAATTCCTCTTCG |
| C5_100 | 5 | Minus | 15,899,892 | 15,899,868 | At5g40450 | CAAGTTGAATGATCTCACCGATGTC |
| C5_101 | 5 | Plus | 15,941,036 | 15,941,060 | At5g40490 | TGTATGGTGGGTCATACGGTGAACC |
| C5_102 | 5 | Plus | 15,959,739 | 15,959,763 | At5g40560 | TCCAGCAGGTCAAATTGACAACAAC |
| C5_103 | 5 | Plus | 16,182,669 | 16,182,693 | At5g41140 | CAACAAAATCAAAATCGTTTTCAAG |
| C5_104 | 5 | Minus | 16,302,882 | 16,302,858 | At5g41450 | CTTAGTCAGCAACCTCCTACGGAAA |
| C5_105 | 5 | Plus | 16,315,405 | 16,315,429 | At5g41490 | GGATCTCCAAGTACCTCAAGGCCAA |
| C5_106 | 5 | Plus | 16,455,776 | 16,455,800 | At5g41820 | CGCTTTTCCTCGTGGCCTTCAGATG |
| C5_107 | 5 | Plus | 16,660,047 | 16,660,071 | At5g42390 | TGCCAAACAAAGTTCCACCGAACAG |
| C5_108 | 5 | Plus | 16,694,618 | 16,694,642 | At5g42460 | ACTTTCAGTTTCTGGCAGAGGCTGG |
| C5_109 | 5 | Plus | 16,768,641 | 16,768,665 | At5g42600 | AGATCATGACGGTCAAAAAAGTGCA |
| C5_110 | 5 | Plus | 16,891,227 | 16,891,251 | At5g42830 | GTCAACCAAATATGCGGAACAACCT |
| C5_111 | 5 | Plus | 17,011,680 | 17,011,704 | At5g43090 | ATCAACATGAAACAGATTTGCTCGT |
| C5_112 | 5 | Minus | 17,177,370 | 17,177,346 | At5g43470 | GTTCGTTTAGCACGTAAGGCATTCC |
| C5_113 | 5 | Minus | 17,400,955 | 17,400,931 | At5g43950 | ATGTATTTACAAGGATCTGCTTTGC |
| C5_114 | 5 | Plus | 17,530,122 | 17,530,146 | At5g44230 | CATCAGAGGTCACAGGGAAGGTAAT |
| C5_115 | 5 | Minus | 17,600,636 | 17,600,612 | At5g44400 | TGATGGCGAAAATCCCCGAATCCCA |
| C5_116 | 5 | Plus | 17,654,527 | 17,654,551 | At5g44530 | CTATACCGCAACACCGTCTGATATG |
| C5_117 | 5 | Minus | 17,747,897 | 17,747,873 | At5g44700 | AACGGCGAGACGATAGCTGTGAAGA |
| C5_118 | 5 | Minus | 17,905,770 | 17,905,746 | At5g45080 | CGAGGCTGTATTCGTAGTCAAATTA |
| C5_119 | 5 | Plus | 18,043,885 | 18,043,909 | At5g45260 | CCAGTAGAGGTTTCCGGTTATGAAG |
| C5_120 | 5 | Minus | 18,417,491 | 18,417,467 | At5g46130 | CTTCCTCAGTATATGTTGTATGAGT |
| C5_121 | 5 | Minus | 18,592,325 | 18,592,301 | At5g46540 | GCAACAAGGCTTCAAATTAGGACTG |
| C5_122 | 5 | Plus | 18,820,808 | 18,820,832 | At5g47050 | AAACTCCACCGCAAGTTATAGGACG |
| C5_123 | 5 | Plus | 18,908,348 | 18,908,372 | At5g47280 | GTCGCTATGGGCACTAGCTACTATG |
| C5_124 | 5 | Minus | 19,236,442 | 19,236,418 | At5g48150 | GATGCATCGAGATTTGGTTGCCTCG |
| C5_125 | 5 | Plus | 19,434,426 | 19,434,450 | At5g48620 | TTGCCCACATACATCTAGTGCATTG |
| C5_126 | 5 | Minus | 19,482,707 | 19,482,683 | At5g48740 | TGCTTTGCTTGCTTGCGGTTAAAGG |
| C5_127 | 5 | Minus | 19,551,747 | 19,551,723 | At5g48930 | CGATTGTAACGGTGCTGGTGTTCTC |
| C5_128 | 5 | Plus | 19,679,431 | 19,679,455 | At5g49250 | CTTGAATCGTTCTTATTGTTGTGGC |
| C5_129 | 5 | Plus | 19,763,187 | 19,763,211 | At5g49440 | TGCCTTAAACGGAGAGAGACACGCC |
| C5_130 | 5 | Minus | 19,965,308 | 19,965,284 | At5g49830 | GATTCGCCATATCTTTGTTGATGCG |
| C5_131 | 5 | Plus | 20,106,857 | 20,106,881 | At5g50130 | TGAGTATGTGGCTCTCAAGCTCTGC |
| C5_132 | 5 | Plus | 20,406,660 | 20,406,684 | At5g50850 | CGTACTTGGATGCACCGGTCGAGAG |
| C5_133 | 5 | Plus | 20,732,108 | 20,732,132 | At5g51740 | CGACATGTAGCTGAGGGAATAACTA |
| C5_134 | 5 | Minus | 21,224,594 | 21,224,570 | At5g53050 | AAGGCAAGAATGTCGCCTCTCCTGC |
| C5_135 | 5 | Plus | 21,424,546 | 21,424,570 | At5g53470 | TGAGAACTTGCGAAAGGTCGAAACT |
| C5_136 | 5 | Plus | 21,557,416 | 21,557,440 | At5g53790 | CTTTTCGGATTAGTTGCAAAACATT |
| C5_137 | 5 | Minus | 21,646,301 | 21,646,277 | NO_GENE | TGCAAATATTGAACGCGAGGCTGAT |
| C5_138 | 5 | Minus | 21,756,347 | 21,756,323 | At5g54280 | AAACCTATTTGACTGGCTTGTTGAG |
| C5_139 | 5 | Plus | 21,800,811 | 21,800,835 | At5g54390 | TTCTTTTGGGTGTACTAGGATGTCC |
| C5_140 | 5 | Minus | 21,901,877 | 21,901,853 | At5g54620 | GCCTCTCGTTTGTTCTTACGCACTT |
| C5_141 | 5 | Minus | 21,946,252 | 21,946,228 | At5g54720 | AGCCTTTTCAAGACATCAACCAGGT |
| C5_142 | 5 | Minus | 22,262,105 | 22,262,081 | At5g55680 | GACTTCCCTGGAATTGTAGTGGGAG |
| C5_143 | 5 | Minus | 22,538,674 | 22,538,650 | At5g56360 | AGGCTGATCGGGTACGCAAAGAATA |
| C5_144 | 5 | Minus | 22,553,924 | 22,553,900 | At5g56380 | GCTGAAACTCAAATACACTGACAGC |
| C5_145 | 5 | Minus | 22,699,565 | 22,699,541 | At5g56850 | AGCAACAACAAGTGGACGAACATTG |
| C5_146 | 5 | Minus | 22,776,070 | 22,776,046 | At5g56990 | TTATACTACCAGTACCATAAGAGCG |
| C5_147 | 5 | Plus | 23,260,982 | 23,261,006 | At5g58190 | GTTAGTATGACTTGCCCTATTTATG |
| C5_148 | 5 | Plus | 23,467,988 | 23,468,012 | At5g58820 | CATCAGGACTTTCCATCCAAAATGG |
| C5_149 | 5 | Minus | 23,568,049 | 23,568,025 | At5g59090 | CACAGCAGCTGGAAATGCAGTCAAA |
| C5_150 | 5 | Plus | 23,752,941 | 23,752,965 | At5g59660 | TTCCAAGTTAGAGGTGAGTCCTATG |
| C5_151 | 5 | Plus | 24,437,333 | 24,437,357 | At5g61470 | TGTTAAGTCTCTCACTTCAGATGTG |
| C5_152 | 5 | Minus | 24,449,190 | 24,449,166 | At5g61500 | GTCAACTTATCTTGTGGCTCATGAA |
| C5_153 | 5 | Minus | 24,793,552 | 24,793,528 | At5g62470 | TTCTTCAGCTTCAATTCAATGGACG |
| C5_154 | 5 | Plus | 25,003,286 | 25,003,310 | At5g63040 | ATCCAATACAATAGAAATGACGGGA |
| C5_155 | 5 | Minus | 25,180,961 | 25,180,937 | At5g63620 | CGGATATCATTGCTGTGGATGTTCA |
| C5_156 | 5 | Plus | 25,404,868 | 25,404,892 | At5g64220 | TGATGTACCAGAAACACTTTCGATG |
| C5_157 | 5 | Minus | 25,476,773 | 25,476,749 | At5g64430 | GGCACCTATAGGAACAAAAGCAGCA |
| C5_158 | 5 | Plus | 25,480,714 | 25,480,738 | At5g64440 | AACCGGAGCATGATGTTGTCATTGT |
| C5_159 | 5 | Plus | 25,588,861 | 25,588,885 | At5g64730 | CCACCCGAAGGAAGACTGTATGTTG |
| C5_160 | 5 | Minus | 25,790,927 | 25,790,903 | At5g65240 | AGGATAATCACTTAACTGATCGCAT |
| C5_161 | 5 | Minus | 25,881,122 | 25,881,098 | At5g65460 | CCGCATCTCAGTTCAGTTTCTCTGT |
| C5_162 | 5 | Plus | 25,969,701 | 25,969,725 | At5g65670 | GGTAAAGTCTATTCTTCTCTCTGCT |
| C5_163 | 5 | Plus | 25,982,005 | 25,982,029 | At5g65690 | TCTAGGTACCCAATATGCTGGAGAA |
| C5_164 | 5 | Plus | 26,072,258 | 26,072,282 | At5g65900 | CATTCATCCGAAAACGAAGAGATTA |
| C5_165 | 5 | Minus | 26,138,403 | 26,138,379 | At5g66080 | TTGCCTGATCGGTGTGGTCTGCGAT |
| C5_166 | 5 | Plus | 26,313,794 | 26,313,818 | At5g66640 | AGCTAGCTAAGCAGGCGGAAGAAAA |
| C5_167 | 5 | Minus | 26,593,289 | 26,593,265 | At5g67370 | GTTACTATGATTCTTCGTCGATCAT |

# Supplemental Table 5.

Informative SFP-markers used for genetic mapping in 98 lines of the Col-0-/L*er* RIL population (Lister and Dean, 1993). SFP-markers were named based on chromosome location (C1, C2, C3 and C5) and then numbered according to their position on the respective chromosome, from top to bottom. Markers with no corresponding gene in the TIGR genome annotation vers.3.0 were labelled as ‘NO_GENE’.
