# Supplementary material for: A High-Resolution Map of Arabidopsis Recombinant Inbred Lines by Whole-Genome Exon Array Hybridization
Source: PLoS Genet. 2006 Sep 15;2(9):e144. doi: 10.1371/journal.pgen.0020144 (PMC1564425; doi:10.1371/journal.pgen.0020144)
Supplement: Supplementary file 16 [file pgen.0020144.st007.doc]

| chrom. 1 |  | chrom. 2 |  | chrom. 3 |  | chrom. 4 |  | chrom. 5 |  |
| --- | --- | --- | --- | --- | --- | --- | --- | --- | --- |
| marker | cM | marker | cM | marker | cM | marker | cM | marker | cM |
| C1_001 | 1.00 | C2_001 | 1.60 | C3_001 | 0.50 | C4_001 | 2.70 | C5_001 | 1.00 |
| C1_002 | 1.50 | C2_002 | 2.60 | C3_002 | 1.00 | C4_002 | 3.20 | C5_002 | 1.50 |
| C1_003 | 2.00 | C2_003 | 3.10 | C3_003 | 1.50 | C4_003 | 3.70 | C5_003 | 2.00 |
| C1_004 | 2.50 | C2_004 | 4.10 | C3_004 | 2.00 | C4_004 | 4.20 | C5_004 | 2.50 |
| C1_005 | 3.00 | C2_005 | 4.60 | C3_005 | 3.00 | C4_005 | 5.80 | C5_005 | 3.00 |
| C1_006 | 3.50 | C2_006 | 5.10 | C3_006 | 3.50 | C4_006 | 6.30 | C5_006 | 3.50 |
| C1_007 | 4.50 | C2_007 | 6.10 | C3_007 | 4.00 | C4_007 | 7.30 | C5_007 | 4.50 |
| C1_008 | 5.00 | C2_008 | 6.60 | C3_008 | 4.50 | C4_008 | 7.80 | C5_008 | 5.00 |
| C1_009 | 5.50 | C2_009 | 7.10 | C3_009 | 5.00 | C4_009 | 8.30 | C5_009 | 5.50 |
| C1_010 | 6.00 | C2_010 | 7.60 | C3_010 | 5.50 | C4_010 | 8.80 | C5_010 | 6.00 |
| C1_011 | 6.50 | C2_011 | 8.10 | C3_011 | 6.00 | C4_011 | 11.50 | C5_011 | 6.50 |
| C1_012 | 7.00 | C2_012 | 8.60 | C3_012 | 6.50 | C4_012 | 12.00 | C5_012 | 7.00 |
| C1_013 | 7.50 | C2_013 | 9.10 | C3_013 | 7.00 | C4_013 | 12.50 | C5_013 | 7.50 |
| C1_014 | 8.00 | C2_014 | 10.10 | C3_014 | 7.50 | C4_014 | 13.00 | C5_014 | 8.00 |
| C1_015 | 8.50 | C2_015 | 10.60 | C3_015 | 8.00 | C4_015 | 13.50 | C5_015 | 8.50 |
| C1_016 | 9.00 | C2_016 | 11.60 | C3_016 | 8.50 | C4_016 | 14.00 | C5_016 | 9.00 |
| C1_017 | 9.50 | C2_017 | 12.10 | C3_017 | 9.00 | C4_017 | 14.50 | C5_017 | 9.50 |
| C1_018 | 10.00 | C2_018 | 13.10 | C3_018 | 10.00 | C4_018 | 15.00 | C5_018 | 11.60 |
| C1_019 | 10.50 | C2_019 | 13.60 | C3_019 | 10.50 | C4_019 | 15.50 | C5_019 | 12.10 |
| C1_020 | 11.00 | C2_020 | 14.10 | C3_020 | 11.00 | C4_020 | 16.50 | C5_020 | 12.60 |
| C1_021 | 13.10 | C2_021 | 14.60 | C3_021 | 11.50 | C4_021 | 17.00 | C5_021 | 13.10 |
| C1_022 | 13.60 | C2_022 | 15.10 | C3_022 | 12.00 | C4_022 | 17.50 | C5_022 | 14.10 |
| C1_023 | 14.10 | C2_023 | 15.60 | C3_023 | 12.50 | C4_023 | 18.00 | C5_023 | 15.10 |
| C1_024 | 14.60 | C2_024 | 16.10 | C3_024 | 13.00 | C4_024 | 18.50 | C5_024 | 15.60 |
| C1_025 | 15.10 | C2_025 | 16.60 | C3_025 | 13.50 | C4_025 | 19.00 | C5_025 | 16.10 |
| C1_026 | 15.60 | C2_026 | 17.10 | C3_026 | 14.00 | C4_026 | 19.50 | C5_026 | 16.60 |
| C1_027 | 16.10 | C2_027 | 17.60 | C3_027 | 14.50 | C4_027 | 20.00 | C5_027 | 17.10 |
| C1_028 | 16.60 | C2_028 | 19.20 | C3_028 | 15.00 | C4_028 | 20.50 | C5_028 | 17.60 |
| C1_029 | 17.10 | C2_029 | 19.70 | C3_029 | 15.50 | C4_029 | 21.00 | C5_029 | 18.10 |
| C1_030 | 17.60 | C2_030 | 20.20 | C3_030 | 16.00 | C4_030 | 21.50 | C5_030 | 18.60 |
| C1_031 | 18.10 | C2_031 | 20.70 | C3_031 | 16.50 | C4_031 | 22.00 | C5_031 | 19.10 |
| C1_032 | 19.10 | C2_032 | 21.20 | C3_032 | 17.00 | C4_032 | 22.50 | C5_032 | 19.60 |
| C1_033 | 19.60 | C2_033 | 21.70 | C3_033 | 17.50 | C4_033 | 23.00 | C5_033 | 20.10 |
| C1_034 | 20.10 | C2_034 | 22.20 | C3_034 | 18.00 | C4_034 | 23.50 | C5_034 | 20.60 |
| C1_035 | 20.60 | C2_035 | 22.70 | C3_035 | 19.00 | C4_035 | 25.10 | C5_035 | 21.60 |
| C1_036 | 21.10 | C2_036 | 23.20 | C3_036 | 20.00 | C4_036 | 26.10 | C5_036 | 22.60 |
| C1_037 | 21.60 | C2_037 | 23.70 | C3_037 | 20.50 | C4_037 | 26.60 | C5_037 | 23.10 |
| C1_038 | 22.10 | C2_038 | 24.20 | C3_038 | 21.00 | C4_038 | 27.60 | C5_038 | 23.60 |
| C1_039 | 22.60 | C2_039 | 24.70 | C3_039 | 21.50 | C4_039 | 28.10 | C5_039 | 24.10 |
| C1_040 | 23.10 | C2_040 | 25.20 | C3_040 | 22.00 | C4_040 | 29.10 | C5_040 | 24.60 |
| C1_041 | 23.60 | C2_041 | 25.70 | C3_041 | 23.00 | C4_041 | 29.60 | C5_041 | 25.10 |
| C1_042 | 24.60 | C2_042 | 26.20 | C3_042 | 25.10 | C4_042 | 30.10 | C5_042 | 26.10 |
| C1_043 | 25.10 | C2_043 | 26.70 | C3_043 | 25.60 | C4_043 | 30.60 | C5_043 | 26.60 |
| C1_044 | 25.60 | C2_044 | 27.20 | C3_044 | 26.10 | C4_044 | 31.60 | C5_044 | 27.10 |
| C1_045 | 26.10 | C2_045 | 27.70 | C3_045 | 26.60 | C4_045 | 32.10 | C5_045 | 27.60 |
| C1_046 | 26.60 | C2_046 | 28.70 | C3_046 | 28.20 | C4_046 | 32.60 | C5_046 | 28.10 |
| C1_047 | 27.10 | C2_047 | 29.20 | C3_047 | 28.70 | C4_047 | 33.60 | C5_047 | 28.60 |
| C1_048 | 27.60 | C2_048 | 29.70 | C3_048 | 29.20 | C4_048 | 34.10 | C5_048 | 29.10 |
| C1_049 | 28.10 | C2_049 | 30.20 | C3_049 | 29.70 | C4_049 | 35.10 | C5_049 | 29.60 |
| C1_050 | 28.60 | C2_050 | 30.70 | C3_050 | 30.20 | C4_050 | 35.60 | C5_050 | 30.10 |
| C1_051 | 29.10 | C2_051 | 31.20 | C3_051 | 31.20 | C4_051 | 36.10 | C5_051 | 30.60 |
| C1_052 | 29.60 | C2_052 | 31.70 | C3_052 | 31.70 | C4_052 | 36.60 | C5_052 | 31.10 |
| C1_053 | 30.10 | C2_053 | 32.20 | C3_053 | 32.70 | C4_053 | 37.10 | C5_053 | 31.60 |
| C1_054 | 31.70 | C2_054 | 32.70 | C3_054 | 33.70 | C4_054 | 38.10 | C5_054 | 32.10 |
| C1_055 | 32.20 | C2_055 | 34.80 | C3_055 | 34.20 | C4_055 | 38.60 | C5_055 | 32.60 |
| C1_056 | 32.70 | C2_056 | 35.30 | C3_056 | 34.70 | C4_056 | 39.10 | C5_056 | 33.10 |
| C1_057 | 33.20 | C2_057 | 35.80 | C3_057 | 35.70 | C4_057 | 39.60 | C5_057 | 33.60 |
| C1_058 | 33.70 | C2_058 | 36.30 | C3_058 | 36.20 | C4_058 | 40.10 | C5_058 | 34.10 |
| C1_059 | 34.20 | C2_059 | 36.80 | C3_059 | 36.70 | C4_059 | 40.60 | C5_059 | 35.10 |
| C1_060 | 34.70 | C2_060 | 37.30 | C3_060 | 37.20 | C4_060 | 41.10 | C5_060 | 36.10 |
| C1_061 | 35.20 | C2_061 | 37.80 | C3_061 | 38.20 | C4_061 | 41.60 | C5_061 | 37.10 |
| C1_062 | 35.70 | C2_062 | 38.30 | C3_062 | 38.70 | C4_062 | 42.10 | C5_062 | 37.60 |
| C1_063 | 36.70 | C2_063 | 38.80 | C3_063 | 39.70 | C4_063 | 42.60 | C5_063 | 38.10 |
| C1_064 | 37.20 | C2_064 | 39.30 | C3_064 | 40.20 | C4_064 | 43.10 | C5_064 | 38.60 |
| C1_065 | 37.70 | C2_065 | 39.80 | C3_065 | 40.70 | C4_065 | 43.60 | C5_065 | 39.10 |
| C1_066 | 38.20 | C2_066 | 40.30 | C3_066 | 41.20 | C4_066 | 44.10 | C5_066 | 39.60 |
| C1_067 | 38.70 | C2_067 | 40.80 | C3_067 | 41.70 | C4_067 | 44.60 | C5_067 | 40.10 |
| C1_068 | 39.20 | C2_068 | 41.80 | C3_068 | 42.20 | C4_068 | 45.10 | C5_068 | 40.60 |
| C1_069 | 39.70 | C2_069 | 42.30 | C3_069 | 42.70 | C4_069 | 45.60 | C5_069 | 41.10 |
| C1_070 | 40.20 | C2_070 | 42.80 | C3_070 | 43.20 | C4_070 | 46.10 | C5_070 | 41.60 |
| C1_071 | 40.70 | C2_071 | 43.30 | C3_071 | 43.70 | C4_071 | 46.60 | C5_071 | 42.10 |
| C1_072 | 41.20 | C2_072 | 43.80 | C3_072 | 44.20 | C4_072 | 47.10 | C5_072 | 42.60 |
| C1_073 | 41.70 | C2_073 | 44.30 | C3_073 | 44.70 | C4_073 | 47.60 | C5_073 | 43.10 |
| C1_074 | 42.70 | C2_074 | 45.90 | C3_074 | 45.20 | C4_074 | 48.10 | C5_074 | 43.60 |
| C1_075 | 43.20 | C2_075 | 46.90 | C3_075 | 45.70 | C4_075 | 48.60 | C5_075 | 44.10 |
| C1_076 | 44.20 | C2_076 | 47.40 | C3_076 | 46.20 | C4_076 | 49.10 | C5_076 | 44.60 |
| C1_077 | 45.20 | C2_077 | 47.90 | C3_077 | 47.80 | C4_077 | 49.60 | C5_077 | 45.10 |
| C1_078 | 45.70 | C2_078 | 48.40 | C3_078 | 48.80 | C4_078 | 50.10 | C5_078 | 46.70 |
| C1_079 | 46.20 | C2_079 | 49.40 | C3_079 | 49.80 | C4_079 | 51.10 | C5_079 | 47.20 |
| C1_080 | 46.70 | C2_080 | 49.90 | C3_080 | 50.30 | C4_080 | 51.60 | C5_080 | 47.70 |
| C1_081 | 47.20 | C2_081 | 50.40 | C3_081 | 51.30 | C4_081 | 52.10 | C5_081 | 48.20 |
| C1_082 | 47.70 | C2_082 | 50.90 | C3_082 | 51.80 | C4_082 | 52.60 | C5_082 | 48.70 |
| C1_083 | 48.20 | C2_083 | 51.40 | C3_083 | 52.30 | C4_083 | 53.10 | C5_083 | 49.20 |
| C1_084 | 48.70 | C2_084 | 51.90 | C3_084 | 53.30 | C4_084 | 53.60 | C5_084 | 49.70 |
| C1_085 | 49.20 | C2_085 | 52.40 | C3_085 | 53.80 | C4_085 | 54.10 | C5_085 | 50.70 |
| C1_086 | 49.70 | C2_086 | 52.90 | C3_086 | 54.80 | C4_086 | 55.10 | C5_086 | 51.20 |
| C1_087 | 50.20 | C2_087 | 53.40 | C3_087 | 55.30 | C4_087 | 55.60 | C5_087 | 51.70 |
| C1_088 | 50.70 | C2_088 | 55.00 | C3_088 | 55.80 | C4_088 | 56.60 | C5_088 | 52.20 |
| C1_089 | 51.20 | C2_089 | 55.50 | C3_089 | 56.30 | C4_089 | 58.20 | C5_089 | 52.70 |
| C1_090 | 51.70 | C2_090 | 56.50 | C3_090 | 56.80 | C4_090 | 58.70 | C5_090 | 53.20 |
| C1_091 | 53.30 | C2_091 | 57.00 | C3_091 | 57.30 | C4_091 | 59.20 | C5_091 | 53.70 |
| C1_092 | 55.40 | C2_092 | 57.50 | C3_092 | 57.80 | C4_092 | 59.70 | C5_092 | 54.20 |
| C1_093 | 55.90 | C2_093 | 58.00 | C3_093 | 58.30 | C4_093 | 60.20 | C5_093 | 54.70 |
| C1_094 | 56.40 | C2_094 | 58.50 | C3_094 | 58.80 | C4_094 | 60.70 | C5_094 | 55.20 |
| C1_095 | 56.90 | C2_095 | 59.00 | C3_095 | 59.30 | C4_095 | 61.20 | C5_095 | 55.70 |
| C1_096 | 57.40 | C2_096 | 59.50 | C3_096 | 59.80 | C4_096 | 61.70 | C5_096 | 56.20 |
| C1_097 | 57.90 | C2_097 | 60.00 | C3_097 | 60.30 | C4_097 | 63.30 | C5_097 | 56.70 |
| C1_098 | 58.40 | C2_098 | 60.50 | C3_098 | 60.80 | C4_098 | 64.90 | C5_098 | 57.20 |
| C1_099 | 58.90 | C2_099 | 61.00 | C3_099 | 61.30 | C4_099 | 65.40 | C5_099 | 57.70 |
| C1_100 | 59.40 | C2_100 | 61.50 | C3_100 | 61.80 | C4_100 | 65.90 | C5_100 | 58.20 |
| C1_101 | 59.90 | C2_101 | 62.00 | C3_101 | 62.30 | C4_101 | 66.40 | C5_101 | 58.70 |
| C1_102 | 60.90 | C2_102 | 62.50 | C3_102 | 62.80 | C4_102 | 68.00 | C5_102 | 59.20 |
| C1_103 | 61.40 | C2_103 | 63.00 | C3_103 | 63.30 | C4_103 | 68.50 | C5_103 | 59.70 |
| C1_104 | 61.90 | C2_104 | 64.00 | C3_104 | 66.00 | C4_104 | 69.00 | C5_104 | 60.20 |
| C1_105 | 62.40 | C2_105 | 64.50 | C3_105 | 66.50 | C4_105 | 69.50 | C5_105 | 60.70 |
| C1_106 | 62.90 | C2_106 | 66.40 | C3_106 | 67.00 | C4_106 | 70.00 | C5_106 | 61.70 |
| C1_107 | 63.40 |  |  | C3_107 | 67.50 | C4_107 | 72.10 | C5_107 | 62.20 |
| C1_108 | 64.40 |  |  | C3_108 | 68.00 | C4_108 | 72.60 | C5_108 | 62.70 |
| C1_109 | 64.90 |  |  | C3_109 | 68.50 | C4_109 | 73.10 | C5_109 | 63.20 |
| C1_110 | 65.40 |  |  | C3_110 | 69.00 | C4_110 | 74.10 | C5_110 | 63.70 |
| C1_111 | 65.90 |  |  | C3_111 | 69.50 | C4_111 | 75.90 | C5_111 | 64.20 |
| C1_112 | 66.40 |  |  | C3_112 | 70.00 |  |  | C5_112 | 64.70 |
| C1_113 | 66.90 |  |  | C3_113 | 70.50 |  |  | C5_113 | 65.20 |
| C1_114 | 67.40 |  |  | C3_114 | 71.00 |  |  | C5_114 | 65.70 |
| C1_115 | 67.90 |  |  | C3_115 | 71.50 |  |  | C5_115 | 66.20 |
| C1_116 | 68.40 |  |  | C3_116 | 72.00 |  |  | C5_116 | 66.70 |
| C1_117 | 68.90 |  |  | C3_117 | 73.00 |  |  | C5_117 | 67.20 |
| C1_118 | 69.40 |  |  | C3_118 | 73.50 |  |  | C5_118 | 67.70 |
| C1_119 | 69.90 |  |  | C3_119 | 74.50 |  |  | C5_119 | 68.20 |
| C1_120 | 70.40 |  |  | C3_120 | 76.70 |  |  | C5_120 | 69.20 |
| C1_121 | 70.90 |  |  |  |  |  |  | C5_121 | 69.70 |
| C1_122 | 71.40 |  |  |  |  |  |  | C5_122 | 70.20 |
| C1_123 | 71.90 |  |  |  |  |  |  | C5_123 | 70.70 |
| C1_124 | 72.40 |  |  |  |  |  |  | C5_124 | 71.70 |
| C1_125 | 72.90 |  |  |  |  |  |  | C5_125 | 72.20 |
| C1_126 | 73.40 |  |  |  |  |  |  | C5_126 | 72.70 |
| C1_127 | 73.90 |  |  |  |  |  |  | C5_127 | 73.20 |
| C1_128 | 74.40 |  |  |  |  |  |  | C5_128 | 73.70 |
| C1_129 | 74.90 |  |  |  |  |  |  | C5_129 | 74.20 |
| C1_130 | 75.40 |  |  |  |  |  |  | C5_130 | 74.70 |
| C1_131 | 75.90 |  |  |  |  |  |  | C5_131 | 75.20 |
| C1_132 | 76.40 |  |  |  |  |  |  | C5_132 | 75.70 |
| C1_133 | 77.40 |  |  |  |  |  |  | C5_133 | 76.20 |
| C1_134 | 77.90 |  |  |  |  |  |  | C5_134 | 76.70 |
| C1_135 | 78.40 |  |  |  |  |  |  | C5_135 | 77.20 |
| C1_136 | 78.90 |  |  |  |  |  |  | C5_136 | 78.20 |
| C1_137 | 79.40 |  |  |  |  |  |  | C5_137 | 78.70 |
| C1_138 | 80.40 |  |  |  |  |  |  | C5_138 | 79.20 |
| C1_139 | 80.90 |  |  |  |  |  |  | C5_139 | 80.20 |
| C1_140 | 81.40 |  |  |  |  |  |  | C5_140 | 80.70 |
| C1_141 | 81.90 |  |  |  |  |  |  | C5_141 | 81.20 |
| C1_142 | 82.90 |  |  |  |  |  |  | C5_142 | 82.20 |
| C1_143 | 83.40 |  |  |  |  |  |  | C5_143 | 82.70 |
| C1_144 | 84.40 |  |  |  |  |  |  | C5_144 | 83.20 |
| C1_145 | 85.40 |  |  |  |  |  |  | C5_145 | 83.70 |
| C1_146 | 85.90 |  |  |  |  |  |  | C5_146 | 84.20 |
| C1_147 | 86.40 |  |  |  |  |  |  | C5_147 | 84.70 |
| C1_148 | 86.90 |  |  |  |  |  |  | C5_148 | 85.20 |
| C1_149 | 87.40 |  |  |  |  |  |  | C5_149 | 85.70 |
| C1_150 | 87.90 |  |  |  |  |  |  | C5_150 | 86.20 |
| C1_151 | 91.20 |  |  |  |  |  |  | C5_151 | 86.70 |
| C1_152 | 91.70 |  |  |  |  |  |  | C5_152 | 87.20 |
| C1_153 | 92.20 |  |  |  |  |  |  | C5_153 | 87.70 |
| C1_154 | 92.70 |  |  |  |  |  |  | C5_154 | 88.70 |
| C1_155 | 93.20 |  |  |  |  |  |  | C5_155 | 89.20 |
| C1_156 | 93.70 |  |  |  |  |  |  | C5_156 | 89.70 |
| C1_157 | 94.20 |  |  |  |  |  |  | C5_157 | 90.20 |
| C1_158 | 94.70 |  |  |  |  |  |  | C5_158 | 90.70 |
| C1_159 | 95.20 |  |  |  |  |  |  | C5_159 | 91.20 |
| C1_160 | 95.70 |  |  |  |  |  |  | C5_160 | 91.70 |
| C1_161 | 96.20 |  |  |  |  |  |  | C5_161 | 92.70 |
| C1_162 | 96.70 |  |  |  |  |  |  | C5_162 | 93.20 |
| C1_163 | 97.70 |  |  |  |  |  |  | C5_163 | 94.20 |
| C1_164 | 98.20 |  |  |  |  |  |  | C5_164 | 94.70 |
| C1_165 | 98.70 |  |  |  |  |  |  | C5_165 | 95.20 |
| C1_166 | 99.20 |  |  |  |  |  |  | C5_166 | 95.70 |
| C1_167 | 99.70 |  |  |  |  |  |  | C5_167 | 98.80 |
| C1_168 | 100.20 |  |  |  |  |  |  |  |  |
| C1_169 | 100.70 |  |  |  |  |  |  |  |  |
| C1_170 | 101.20 |  |  |  |  |  |  |  |  |
| C1_171 | 101.70 |  |  |  |  |  |  |  |  |
| C1_172 | 104.70 |  |  |  |  |  |  |  |  |

**Supplemental Table 7.**

Genetic linkage map with centimorgan distances for the *Arabidopsis* genome based on 676 informative SFP-markers for the Col-0/L*er* RIL-population (Lister and Dean, 1993). The Kosambi-mapping function was used to generate the linkage map with a modified MAPMAKER/EXP vers.3.0 (Lander et al., 1987) software.
