# Supplementary material for: A High-Resolution Map of Arabidopsis Recombinant Inbred Lines by Whole-Genome Exon Array Hybridization
Source: PLoS Genet. 2006 Sep 15;2(9):e144. doi: 10.1371/journal.pgen.0020144 (PMC1564425; doi:10.1371/journal.pgen.0020144)
Supplement: Supplementary file 17 [file pgen.0020144.st008.doc]

|  | chrom. | Ave. interval dist. (kb) | Min. interval dist. (kb) | Max. interval dist (kb) | Ave. gene no./ interval | Min. gene no./ interval | Max. gene no. /interval |
| --- | --- | --- | --- | --- | --- | --- | --- |
| informative SFP-markers |  |  |  |  |  |  |  |
|  | 1 | 174 | 7.60 | 1307 | 42 | 3 | 234 |
|  | 2 | 184 | 4.96 | 1,253 | 46 | 2 | 256 |
|  | 3 | 195 | 0.003 | 1,902 | 46 | 1 | 269 |
|  | 4 | 166 | 2.30 | 2,450 | 41 | 1 | 492 |
|  | 5 | 162 | 3.67 | 1,972 | 41 | 2 | 343 |
| Ave |  | 176 | 3.71 | 1,777 | 43 | 1.8 | 319 |
| interval SFP-markers |  |  |  |  |  |  |  |
|  | 1 | 143 | 0.007 | 1,391 | 36 | 1 | 325 |
|  | 2 | 159 | 0.022 | 1,361 | 40 | 1 | 277 |
|  | 3 | 158 | 0.021 | 2,299 | 41 | 1 | 461 |
|  | 4 | 137 | 0.025 | 1,978 | 35 | 1 | 391 |
|  | 5 | 128 | 0.010 | 2,542 | 33 | 1 | 466 |
| Ave |  | 145 | 0.017 | 1,914 | 37 | 1 | 384 |

# Supplemental Table 8.

Genome-wide statistics on interval sizes and number of genes per genetic interval based on informative SFP-markers and on interval SFP-markers.
