# Supplementary material for: A High-Resolution Map of Arabidopsis Recombinant Inbred Lines by Whole-Genome Exon Array Hybridization
Source: PLoS Genet. 2006 Sep 15;2(9):e144. doi: 10.1371/journal.pgen.0020144 (PMC1564425; doi:10.1371/journal.pgen.0020144)
Supplement: Supplementary file 18 [file pgen.0020144.st009.doc]

| Chrom. | Marker | strand | Beg.start | End.start | Gene_Beg_Interval | Chrom. | Marker | strand | Beg.last | End.last | Gene_end_interval |
| --- | --- | --- | --- | --- | --- | --- | --- | --- | --- | --- | --- |
| C1 | 1 | Plus | 114,902 | 114,926 | At1g01010 | C1 | 1 | Minus | 1,234,757 | 1,234,733 | At1g04530 |
| C1 | 2 | Plus | 1,238,972 | 1,238,996 | At1g04540 | C1 | 2 | Plus | 1,241,202 | 1,241,226 | At1g04550 |
| C1 | 3 | Minus | 1,435,850 | 1,435,826 | At1g05020 | C1 | 3 | Plus | 1,506,919 | 1,506,943 | At1g05200 |
| C1 | 4 | Plus | 1,506,972 | 1,506,996 | At1g05200 | C1 | 4 | Plus | 1,685,400 | 1,685,424 | At1g05630 |
| C1 | 5 | Plus | 1,686,847 | 1,686,871 | At1g05630 | C1 | 5 | Minus | 2,338,408 | 2,338,384 | At1g07590 |
| C1 | 6 | Minus | 2,355,231 | 2,355,207 | At1g07640 | C1 | 6 | Minus | 2,355,231 | 2,355,207 | At1g07640 |
| C1 | 7 | Plus | 2,378,310 | 2,378,334 | At1g07690 | C1 | 7 | Minus | 2,576,372 | 2,576,348 | At1g08200 |
| C1 | 8 | Plus | 2,646,337 | 2,646,361 | At1g08410 | C1 | 8 | Plus | 2,842,359 | 2,842,383 | At1g08860 |
| C1 | 9 | Plus | 2,889,330 | 2,889,354 | At1g08990 | C1 | 9 | Minus | 2,973,383 | 2,973,359 | At1g09210 |
| C1 | 10 | Plus | 2,993,482 | 2,993,506 | At1g09260 | C1 | 10 | Plus | 3,769,003 | 3,769,027 | At1g11250 |
| C1 | 11 | Plus | 3,779,315 | 3,779,339 | At1g11260 | C1 | 11 | Plus | 4,156,857 | 4,156,881 | At1g12240 |
| C1 | 12 | Plus | 4,224,469 | 4,224,493 | At1g12410 | C1 | 12 | Plus | 4,224,469 | 4,224,493 | At1g12410 |
| C1 | 13 | Plus | 4,261,272 | 4,261,296 | At1g12490 | C1 | 13 | Plus | 4,275,698 | 4,275,722 | At1g12550 |
| C1 | 14 | Minus | 4,290,755 | 4,290,731 | At1g12610 | C1 | 14 | Minus | 4,323,107 | 4,323,083 | At1g12700 |
| C1 | 15 | Plus | 4,331,319 | 4,331,343 | At1g12720 | C1 | 15 | Plus | 4,460,075 | 4,460,099 | At1g13080 |
| C1 | 16 | Minus | 4,507,470 | 4,507,446 | At1g13200 | C1 | 16 | Plus | 4,605,488 | 4,605,512 | At1g13420 |
| C1 | 17 | Plus | 4,606,713 | 4,606,737 | At1g13430 | C1 | 17 | Plus | 4,618,103 | 4,618,127 | At1g13460 |
| C1 | 18 | Minus | 4,628,059 | 4,628,035 | At1g13500 | C1 | 18 | Minus | 4,628,059 | 4,628,035 | At1g13500 |
| C1 | 19 | Plus | 4,795,942 | 4,795,966 | At1g13990 | C1 | 19 | Minus | 4,932,558 | 4,932,534 | At1g14420 |
| C1 | 20 | Minus | 5,119,147 | 5,119,123 | At1g14850 | C1 | 20 | Minus | 5,266,095 | 5,266,071 | At1g15300 |
| C1 | 21 | Minus | 5,308,743 | 5,308,719 | At1g15440 | C1 | 21 | Minus | 5,427,594 | 5,427,570 | At1g15770 |
| C1 | 22 | Plus | 5,456,669 | 5,456,693 | At1g15860 | C1 | 22 | Minus | 5,773,119 | 5,773,095 | At1g16870 |
| C1 | 23 | Plus | 5,854,427 | 5,854,451 | At1g17130 | C1 | 23 | Plus | 5,854,427 | 5,854,451 | At1g17130 |
| C1 | 24 | Plus | 5,866,270 | 5,866,294 | At1g17150 | C1 | 24 | Minus | 5,897,092 | 5,897,068 | At1g17240 |
| C1 | 25 | Plus | 6,002,072 | 6,002,096 | At1g17460 | C1 | 25 | Plus | 6,066,188 | 6,066,212 | At1g17630 |
| C1 | 26 | Plus | 6,109,919 | 6,109,943 | At1g17750 | C1 | 26 | Minus | 6,163,985 | 6,163,961 | At1g17920 |
| C1 | 27 | Minus | 6,229,150 | 6,229,126 | At1g18100 | C1 | 27 | Plus | 6,238,771 | 6,238,795 | At1g18130 |
| C1 | 28 | Minus | 6,279,999 | 6,279,975 | At1g18260 | C1 | 28 | Plus | 6,365,689 | 6,365,713 | At1g18485 |
| C1 | 29 | Minus | 6,429,539 | 6,429,515 | At1g18670 | C1 | 29 | Plus | 6,553,436 | 6,553,460 | At1g18960 |
| C1 | 30 | Plus | 6,590,777 | 6,590,801 | At1g19090 | C1 | 30 | Minus | 7,028,540 | 7,028,516 | At1g20290 |
| C1 | 31 | Plus | 7,071,016 | 7,071,040 | At1g20390 | C1 | 31 | Minus | 7,074,185 | 7,074,161 | At1g20400 |
| C1 | 32 | Minus | 7,098,631 | 7,098,607 | At1g20490 | C1 | 32 | Plus | 7,511,354 | 7,511,378 | At1g21450 |
| C1 | 33 | Minus | 7,544,481 | 7,544,457 | At1g21530 | C1 | 33 | Plus | 7,635,917 | 7,635,941 | At1g21730 |
| C1 | 34 | Minus | 7,705,410 | 7,705,386 | At1g21920 | C1 | 34 | Minus | 7,723,715 | 7,723,691 | At1g21950 |
| C1 | 35 | Plus | 7,747,096 | 7,747,120 | At1g22000 | C1 | 35 | Minus | 7,812,480 | 7,812,456 | At1g22130 |
| C1 | 36 | Plus | 7,847,461 | 7,847,485 | At1g22220 | C1 | 36 | Minus | 7,953,839 | 7,953,815 | At1g22520 |
| C1 | 37 | Minus | 7,953,845 | 7,953,821 | At1g22520 | C1 | 37 | Minus | 7,953,845 | 7,953,821 | At1g22520 |
| C1 | 38 | Minus | 8,044,429 | 8,044,405 | At1g22720 | C1 | 38 | Minus | 8,082,241 | 8,082,217 | At1g22850 |
| C1 | 39 | Plus | 8,096,153 | 8,096,177 | At1g22880 | C1 | 39 | Plus | 8,097,218 | 8,097,242 | At1g22880 |
| C1 | 40 | Minus | 8,111,181 | 8,111,157 | At1g22920 | C1 | 40 | Plus | 8,148,822 | 8,148,846 | At1g23010 |
| C1 | 41 | Plus | 8,168,942 | 8,168,966 | At1g23050 | C1 | 41 | Minus | 8,273,528 | 8,273,504 | At1g23320 |
| C1 | 42 | Plus | 8,323,077 | 8,323,101 | At1g23440 | C1 | 42 | Plus | 8,339,788 | 8,339,812 | At1g23500 |
| C1 | 43 | Minus | 8,352,829 | 8,352,805 | At1g23560 | C1 | 43 | Plus | 8,503,997 | 8,504,021 | At1g24030 |
| C1 | 44 | Plus | 8,504,240 | 8,504,264 | At1g24030 | C1 | 44 | Plus | 8,694,216 | 8,694,240 | At1g24530 |
| C1 | 45 | Plus | 8,694,377 | 8,694,401 | At1g24530 | C1 | 45 | Plus | 8,694,377 | 8,694,401 | At1g24530 |
| C1 | 46 | Minus | 8,714,515 | 8,714,491 | At1g24590 | C1 | 46 | Minus | 8,714,515 | 8,714,491 | At1g24590 |
| C1 | 47 | Minus | 8,911,865 | 8,911,841 | At1g25400 | C1 | 47 | Minus | 9,162,493 | 9,162,469 | At1g26510 |
| C1 | 48 | Plus | 9,190,322 | 9,190,346 | At1g26590 | C1 | 48 | Plus | 9,190,322 | 9,190,346 | At1g26590 |
| C1 | 49 | Minus | 9,319,841 | 9,319,817 | At1g26900 | C1 | 49 | Minus | 9,399,181 | 9,399,157 | At1g27070 |
| C1 | 50 | Minus | 9,413,698 | 9,413,674 | At1g27110 | C1 | 50 | Plus | 9,727,239 | 9,727,263 | At1g27920 |
| C1 | 51 | Plus | 9,750,503 | 9,750,527 | At1g27980 | C1 | 51 | Plus | 9,949,499 | 9,949,523 | At1g28350 |
| C1 | 52 | Plus | 9,964,594 | 9,964,618 | At1g28380 | C1 | 52 | Plus | 10,078,819 | 10,078,843 | At1g28680 |
| C1 | 53 | Minus | 10,084,809 | 10,084,785 | At1g28700 | C1 | 53 | Minus | 10,158,617 | 10,158,593 | At1g29080 |
| C1 | 54 | Minus | 10,201,683 | 10,201,659 | At1g29190 | C1 | 54 | Plus | 10,208,272 | 10,208,296 | At1g29200 |
| C1 | 55 | Plus | 10,320,049 | 10,320,073 | At1g29490 | C1 | 55 | Plus | 10,359,551 | 10,359,575 | At1g29650 |
| C1 | 56 | Minus | 10,396,593 | 10,396,569 | At1g29720 | C1 | 56 | Plus | 10,445,056 | 10,445,080 | At1g29830 |
| C1 | 57 | Minus | 10,458,218 | 10,458,194 | At1g29870 | C1 | 57 | Minus | 10,499,363 | 10,499,339 | At1g29965 |
| C1 | 58 | Minus | 10,584,729 | 10,584,705 | At1g30120 | C1 | 58 | Plus | 10,613,649 | 10,613,673 | At1g30180 |
| C1 | 59 | Minus | 10,641,882 | 10,641,858 | At1g30240 | C1 | 59 | Minus | 10,641,882 | 10,641,858 | At1g30240 |
| C1 | 60 | Plus | 10,701,242 | 10,701,266 | At1g30340 | C1 | 60 | Plus | 10,701,350 | 10,701,374 | At1g30340 |
| C1 | 61 | Minus | 10,773,577 | 10,773,553 | At1g30460 | C1 | 61 | Minus | 10,908,300 | 10,908,276 | At1g30755 |
| C1 | 62 | Plus | 10,985,840 | 10,985,864 | At1g30850 | C1 | 62 | Minus | 11,010,333 | 11,010,309 | At1g30922 |
| C1 | 63 | Minus | 11,010,486 | 11,010,462 | At1g30920 | C1 | 63 | Minus | 11,011,845 | 11,011,821 | At1g30920 |
| C1 | 64 | Minus | 11,023,206 | 11,023,182 | At1g30940 | C1 | 64 | Minus | 11,023,224 | 11,023,200 | At1g30940 |
| C1 | 65 | Plus | 11,037,425 | 11,037,449 | At1g30950 | C1 | 65 | Minus | 11,064,799 | 11,064,775 | At1g31030 |
| C1 | 66 | Minus | 11,064,849 | 11,064,825 | At1g31030 | C1 | 66 | Minus | 11,238,519 | 11,238,495 | At1g31370 |
| C1 | 67 | Minus | 11,238,527 | 11,238,503 | At1g31370 | C1 | 67 | Minus | 11,289,912 | 11,289,888 | At1g31540 |
| C1 | 68 | Minus | 11,291,468 | 11,291,444 | At1g31540 | C1 | 68 | Plus | 11,433,356 | 11,433,380 | At1g31850 |
| C1 | 69 | Plus | 11,460,895 | 11,460,919 | At1g31910 | C1 | 69 | Plus | 11,468,312 | 11,468,336 | At1g31930 |
| C1 | 70 | Plus | 11,477,516 | 11,477,540 | At1g31950 | C1 | 70 | Plus | 11,534,045 | 11,534,069 | At1g32060 |
| C1 | 71 | Plus | 11,554,525 | 11,554,549 | At1g32120 | C1 | 71 | Plus | 11,570,057 | 11,570,081 | At1g32160 |
| C1 | 72 | Plus | 11,570,253 | 11,570,277 | At1g32160 | C1 | 72 | Plus | 11,570,253 | 11,570,277 | At1g32160 |
| C1 | 73 | Plus | 11,643,074 | 11,643,098 | At1g32270 | C1 | 73 | Minus | 11,690,894 | 11,690,870 | At1g32400 |
| C1 | 74 | Plus | 11,698,485 | 11,698,509 | At1g32410 | C1 | 74 | Plus | 12,065,008 | 12,065,032 | At1g33260 |
| C1 | 75 | Plus | 12,092,650 | 12,092,674 | At1g33360 | C1 | 75 | Minus | 12,245,005 | 12,244,981 | At1g33780 |
| C1 | 76 | Plus | 12,269,014 | 12,269,038 | At1g33811 | C1 | 76 | Plus | 12,470,824 | 12,470,848 | At1g34240 |
| C1 | 77 | Plus | 12,486,742 | 12,486,766 | At1g34260 | C1 | 77 | Minus | 12,616,999 | 12,616,975 | At1g34510 |
| C1 | 78 | Plus | 12,628,003 | 12,628,027 | At1g34530 | C1 | 78 | Plus | 12,965,690 | 12,965,714 | At1g35330 |
| C1 | 79 | Minus | 13,037,584 | 13,037,560 | At1g35450 | C1 | 79 | Minus | 13,163,633 | 13,163,609 | At1g35630 |
| C1 | 80 | Minus | 13,189,167 | 13,189,143 | At1g35650 | C1 | 80 | Minus | 13,279,725 | 13,279,701 | At1g35780 |
| C1 | 81 | Minus | 13,280,893 | 13,280,869 | At1g35780 | C1 | 81 | Minus | 13,280,893 | 13,280,869 | At1g35780 |
| C1 | 82 | Plus | 13,330,299 | 13,330,323 | At1g35840 | C1 | 82 | Minus | 14,133,392 | 14,133,368 | At1g37110 |
| C1 | 83 | Minus | 14,195,909 | 14,195,885 | At1g37180 | C1 | 83 | Minus | 15,113,633 | 15,113,609 | At1g41820 |
| C1 | 84 | Plus | 15,159,650 | 15,159,674 | At1g41850 | C1 | 84 | Plus | 15,658,208 | 15,658,232 | At1g42970 |
| C1 | 85 | Minus | 15,665,899 | 15,665,875 | At1g42990 | C1 | 85 | Plus | 15,793,718 | 15,793,742 | At1g43160 |
| C1 | 86 | Plus | 15,845,921 | 15,845,945 | At1g43250 | C1 | 86 | Minus | 15,975,246 | 15,975,222 | At1g43650 |
| C1 | 87 | Plus | 16,008,415 | 16,008,439 | At1g43690 | C1 | 87 | Plus | 16,008,415 | 16,008,439 | At1g43690 |
| C1 | 88 | Minus | 16,058,898 | 16,058,874 | At1g43760 | C1 | 88 | Plus | 16,158,163 | 16,158,187 | At1g43880 |
| C1 | 89 | Plus | 16,177,580 | 16,177,604 | At1g43890 | C1 | 89 | Minus | 16,224,952 | 16,224,928 | At1g43990 |
| C1 | 90 | Plus | 16,323,712 | 16,323,736 | At1g44150 | C1 | 90 | Plus | 16,323,712 | 16,323,736 | At1g44150 |
| C1 | 91 | Plus | 16,343,605 | 16,343,629 | At1g44190 | C1 | 91 | Plus | 16,343,605 | 16,343,629 | At1g44190 |
| C1 | 92 | Plus | 16,417,414 | 16,417,438 | At1g44740 | C1 | 92 | Minus | 16,626,787 | 16,626,763 | At1g45170 |
| C1 | 93 | Plus | 16,890,812 | 16,890,836 | At1g47220 | C1 | 93 | Plus | 16,890,812 | 16,890,836 | At1g47220 |
| C1 | 94 | Minus | 16,908,725 | 16,908,701 | At1g47260 | C1 | 94 | Plus | 16,914,821 | 16,914,845 | At1g47270 |
| C1 | 95 | Plus | 16,922,580 | 16,922,604 | At1g47290 | C1 | 95 | Minus | 16,954,475 | 16,954,451 | At1g47370 |
| C1 | 96 | Minus | 16,982,523 | 16,982,499 | At1g47420 | C1 | 96 | Plus | 16,983,986 | 16,984,010 | At1g47430 |
| C1 | 97 | Plus | 16,984,015 | 16,984,039 | At1g47430 | C1 | 97 | Plus | 17,234,516 | 17,234,540 | At1g47890 |
| C1 | 98 | Minus | 17,235,632 | 17,235,608 | At1g47900 | C1 | 98 | Minus | 17,583,932 | 17,583,908 | At1g48660 |
| C1 | 99 | Minus | 17,585,503 | 17,585,479 | At1g48660 | C1 | 99 | Plus | 17,605,978 | 17,606,002 | At1g48720 |
| C1 | 100 | Minus | 17,624,449 | 17,624,425 | At1g48760 | C1 | 100 | Plus | 17,637,907 | 17,637,931 | At1g48800 |
| C1 | 101 | Plus | 17,642,939 | 17,642,963 | At1g48820 | C1 | 101 | Minus | 17,816,334 | 17,816,310 | At1g49270 |
| C1 | 102 | Minus | 17,816,755 | 17,816,731 | At1g49270 | C1 | 102 | Plus | 17,935,907 | 17,935,931 | At1g49570 |
| C1 | 103 | Minus | 17,956,592 | 17,956,568 | At1g49630 | C1 | 103 | Minus | 18,218,973 | 18,218,949 | At1g50300 |
| C1 | 104 | Plus | 18,307,267 | 18,307,291 | At1g50520 | C1 | 104 | Minus | 18,664,893 | 18,664,869 | At1g51460 |
| C1 | 105 | Plus | 18,682,837 | 18,682,861 | At1g51490 | C1 | 105 | Minus | 18,935,690 | 18,935,666 | At1g52030 |
| C1 | 106 | Minus | 18,953,470 | 18,953,446 | At1g52070 | C1 | 106 | Minus | 18,970,946 | 18,970,922 | At1g52100 |
| C1 | 107 | Minus | 18,971,263 | 18,971,239 | At1g52100 | C1 | 107 | Plus | 19,066,398 | 19,066,422 | At1g52310 |
| C1 | 108 | Plus | 19,072,718 | 19,072,742 | At1g52320 | C1 | 108 | Plus | 19,072,866 | 19,072,890 | At1g52320 |
| C1 | 109 | Plus | 19,087,935 | 19,087,959 | At1g52360 | C1 | 109 | Plus | 19,098,753 | 19,098,777 | At1g52380 |
| C1 | 110 | Plus | 19,104,001 | 19,104,025 | At1g52400 | C1 | 110 | Plus | 19,118,472 | 19,118,496 | At1g52420 |
| C1 | 111 | Minus | 19,146,094 | 19,146,070 | At1g52490 | C1 | 111 | Plus | 19,469,044 | 19,469,068 | At1g53300 |
| C1 | 112 | Plus | 19,484,558 | 19,484,582 | At1g53330 | C1 | 112 | Minus | 19,486,646 | 19,486,622 | At1g53340 |
| C1 | 113 | Minus | 19,517,154 | 19,517,130 | At1g53420 | C1 | 113 | Plus | 19,686,283 | 19,686,307 | At1g53830 |
| C1 | 114 | Minus | 19,728,311 | 19,728,287 | At1g53930 | C1 | 114 | Plus | 20,012,071 | 20,012,095 | At1g54730 |
| C1 | 115 | Plus | 20,125,992 | 20,126,016 | At1g55040 | C1 | 115 | Minus | 20,314,314 | 20,314,290 | At1g55520 |
| C1 | 116 | Minus | 20,323,100 | 20,323,076 | At1g55540 | C1 | 116 | Plus | 20,605,036 | 20,605,060 | At1g56150 |
| C1 | 117 | Minus | 20,649,747 | 20,649,723 | At1g56250 | C1 | 117 | Plus | 20,933,665 | 20,933,689 | At1g57630 |
| C1 | 118 | Minus | 20,955,389 | 20,955,365 | At1g57690 | C1 | 118 | Minus | 21,252,007 | 21,251,983 | At1g58350 |
| C1 | 119 | Plus | 21,266,037 | 21,266,061 | At1g58360 | C1 | 119 | Minus | 21,504,436 | 21,504,412 | At1g59453 |
| C1 | 120 | Plus | 21,545,973 | 21,545,997 | At1g59610 | C1 | 120 | Minus | 21,645,672 | 21,645,648 | At1g59780 |
| C1 | 121 | Minus | 21,645,679 | 21,645,655 | At1g59780 | C1 | 121 | Minus | 21,869,122 | 21,869,098 | At1g60250 |
| C1 | 122 | Minus | 21,871,176 | 21,871,152 | At1g60260 | C1 | 122 | Plus | 22,646,951 | 22,646,975 | At1g62240 |
| C1 | 123 | Plus | 22,699,557 | 22,699,581 | At1g62330 | C1 | 123 | Plus | 22,724,820 | 22,724,844 | At1g62370 |
| C1 | 124 | Plus | 22,742,220 | 22,742,244 | At1g62400 | C1 | 124 | Plus | 22,804,233 | 22,804,257 | At1g62540 |
| C1 | 125 | Plus | 22,808,906 | 22,808,930 | At1g62550 | C1 | 125 | Plus | 22,854,637 | 22,854,661 | At1g62660 |
| C1 | 126 | Plus | 22,904,345 | 22,904,369 | At1g62790 | C1 | 126 | Minus | 23,001,549 | 23,001,525 | At1g63010 |
| C1 | 127 | Plus | 23,023,548 | 23,023,572 | At1g63040 | C1 | 127 | Minus | 23,197,833 | 23,197,809 | At1g63490 |
| C1 | 128 | Minus | 23,203,941 | 23,203,917 | At1g63490 | C1 | 128 | Minus | 23,464,461 | 23,464,437 | At1g64150 |
| C1 | 129 | Plus | 23,473,910 | 23,473,934 | At1g64180 | C1 | 129 | Plus | 23,475,891 | 23,475,915 | At1g64180 |
| C1 | 130 | Plus | 23,475,925 | 23,475,949 | At1g64180 | C1 | 130 | Plus | 23,538,333 | 23,538,357 | At1g64355 |
| C1 | 131 | Plus | 23,565,684 | 23,565,708 | At1g64390 | C1 | 131 | Plus | 23,669,956 | 23,669,980 | At1g64625 |
| C1 | 132 | Plus | 23,670,252 | 23,670,276 | At1g64625 | C1 | 132 | Minus | 24,031,722 | 24,031,698 | At1g65580 |
| C1 | 133 | Plus | 24,041,081 | 24,041,105 | At1g65590 | C1 | 133 | Minus | 24,044,042 | 24,044,018 | At1g65610 |
| C1 | 134 | Minus | 24,058,670 | 24,058,646 | At1g65630 | C1 | 134 | Minus | 24,058,670 | 24,058,646 | At1g65630 |
| C1 | 135 | Plus | 24,079,196 | 24,079,220 | At1g65680 | C1 | 135 | Plus | 24,267,317 | 24,267,341 | At1g66130 |
| C1 | 136 | Minus | 24,309,029 | 24,309,005 | At1g66200 | C1 | 136 | Minus | 24,309,029 | 24,309,005 | At1g66200 |
| C1 | 137 | Minus | 24,318,950 | 24,318,926 | At1g66210 | C1 | 137 | Plus | 24,429,430 | 24,429,454 | At1g66420 |
| C1 | 138 | Plus | 24,430,342 | 24,430,366 | At1g66430 | C1 | 138 | Minus | 24,507,990 | 24,507,966 | At1g66630 |
| C1 | 139 | Plus | 24,554,169 | 24,554,193 | At1g66760 | C1 | 139 | Plus | 24,554,169 | 24,554,193 | At1g66760 |
| C1 | 140 | Plus | 24,577,152 | 24,577,176 | At1g66800 | C1 | 140 | Minus | 24,639,793 | 24,639,769 | At1g66960 |
| C1 | 141 | Minus | 24,651,425 | 24,651,401 | At1g66980 | C1 | 141 | Plus | 24,668,754 | 24,668,778 | At1g67030 |
| C1 | 142 | Plus | 24,668,962 | 24,668,986 | At1g67030 | C1 | 142 | Minus | 24,671,590 | 24,671,566 | At1g67040 |
| C1 | 143 | Minus | 24,715,862 | 24,715,838 | At1g67110 | C1 | 143 | Minus | 24,746,528 | 24,746,504 | At1g67120 |
| C1 | 144 | Plus | 24,775,745 | 24,775,769 | At1g67150 | C1 | 144 | Minus | 24,858,213 | 24,858,189 | At1g67320 |
| C1 | 145 | Plus | 24,883,289 | 24,883,313 | At1g67340 | C1 | 145 | Minus | 25,287,936 | 25,287,912 | At1g68380 |
| C1 | 146 | Plus | 25,412,008 | 25,412,032 | At1g68600 | C1 | 146 | Plus | 25,418,594 | 25,418,618 | At1g68620 |
| C1 | 147 | Minus | 25,422,458 | 25,422,434 | At1g68640 | C1 | 147 | Plus | 25,541,068 | 25,541,092 | At1g68870 |
| C1 | 148 | Plus | 25,586,469 | 25,586,493 | At1g68980 | C1 | 148 | Minus | 25,643,776 | 25,643,752 | At1g69140 |
| C1 | 149 | Plus | 25,691,447 | 25,691,471 | At1g69260 | C1 | 149 | Plus | 25,809,831 | 25,809,855 | At1g69560 |
| C1 | 150 | Plus | 25,810,343 | 25,810,367 | At1g69560 | C1 | 150 | Plus | 25,840,440 | 25,840,464 | At1g69610 |
| C1 | 151 | Plus | 25,842,895 | 25,842,919 | At1g69620 | C1 | 151 | Plus | 25,877,580 | 25,877,604 | At1g69710 |
| C1 | 152 | Plus | 25,879,939 | 25,879,963 | At1g69720 | C1 | 152 | Minus | 25,882,366 | 25,882,342 | At1g69730 |
| C1 | 153 | Minus | 25,927,579 | 25,927,555 | At1g69800 | C1 | 153 | Minus | 25,978,698 | 25,978,674 | At1g69900 |
| C1 | 154 | Plus | 25,982,681 | 25,982,705 | At1g69910 | C1 | 154 | Minus | 26,130,441 | 26,130,417 | At1g70300 |
| C1 | 155 | Minus | 26,145,284 | 26,145,260 | At1g70320 | C1 | 155 | Plus | 26,359,502 | 26,359,526 | At1g70820 |
| C1 | 156 | Plus | 26,359,728 | 26,359,752 | At1g70820 | C1 | 156 | Plus | 26,521,654 | 26,521,678 | At1g71270 |
| C1 | 157 | Plus | 26,525,077 | 26,525,101 | At1g71290 | C1 | 157 | Minus | 26,782,599 | 26,782,575 | At1g72110 |
| C1 | 158 | Plus | 26,787,238 | 26,787,262 | At1g72120 | C1 | 158 | Plus | 26,795,783 | 26,795,807 | At1g72140 |
| C1 | 159 | Plus | 26,801,925 | 26,801,949 | At1g72150 | C1 | 159 | Minus | 26,939,259 | 26,939,235 | At1g72480 |
| C1 | 160 | Plus | 26,960,589 | 26,960,613 | At1g72520 | C1 | 160 | Minus | 27,202,590 | 27,202,566 | At1g73270 |
| C1 | 161 | Plus | 27,241,628 | 27,241,652 | At1g73380 | C1 | 161 | Plus | 27,903,986 | 27,904,010 | At1g75280 |
| C1 | 162 | Plus | 27,957,468 | 27,957,492 | At1g75420 | C1 | 162 | Plus | 27,957,468 | 27,957,492 | At1g75420 |
| C1 | 163 | Minus | 28,069,326 | 28,069,302 | At1g75680 | C1 | 163 | Plus | 28,192,925 | 28,192,949 | At1g76040 |
| C1 | 164 | Plus | 28,226,370 | 28,226,394 | At1g76140 | C1 | 164 | Minus | 28,574,005 | 28,573,981 | At1g76960 |
| C1 | 165 | Plus | 28,587,854 | 28,587,878 | At1g76990 | C1 | 165 | Plus | 29,010,453 | 29,010,477 | At1g78070 |
| C1 | 166 | Plus | 29,027,338 | 29,027,362 | At1g78090 | C1 | 166 | Plus | 29,059,280 | 29,059,304 | At1g78150 |
| C1 | 167 | Minus | 29,087,260 | 29,087,236 | At1g78240 | C1 | 167 | Minus | 29,430,285 | 29,430,261 | At1g79150 |
| C1 | 168 | Minus | 29,433,734 | 29,433,710 | At1g79160 | C1 | 168 | Plus | 29,488,237 | 29,488,261 | At1g79310 |
| C1 | 169 | Plus | 29,514,852 | 29,514,876 | At1g79380 | C1 | 169 | Plus | 29,514,852 | 29,514,876 | At1g79380 |
| C1 | 170 | Minus | 29,565,719 | 29,565,695 | At1g79520 | C1 | 170 | Minus | 29,565,719 | 29,565,695 | At1g79520 |
| C1 | 171 | Minus | 29,566,082 | 29,566,058 | At1g79520 | C1 | 171 | Plus | 29,946,981 | 29,947,005 | At1g80580 |
| C1 | 172 | Plus | 29,947,314 | 29,947,338 | At1g80580 | C1 | 172 | Plus | 30,079,980 | 30,080,004 | At1g81020 |
| C2 | 1 | Plus | 81,560 | 81,584 | At2g01010 | C2 | 1 | Plus | 321,175 | 321,199 | At2g01730 |
| C2 | 2 | Plus | 322,605 | 322,629 | At2g01730 | C2 | 2 | Minus | 343,707 | 343,683 | At2g01800 |
| C2 | 3 | Minus | 356,899 | 356,875 | At2g01820 | C2 | 3 | Minus | 356,944 | 356,920 | At2g01820 |
| C2 | 4 | Plus | 384,666 | 384,690 | At2g01850 | C2 | 4 | Plus | 417,373 | 417,397 | At2g01910 |
| C2 | 5 | Minus | 452,335 | 452,311 | At2g01970 | C2 | 5 | Minus | 452,335 | 452,311 | At2g01970 |
| C2 | 6 | Minus | 475,213 | 475,189 | At2g02010 | C2 | 6 | Minus | 475,478 | 475,454 | At2g02010 |
| C2 | 7 | Plus | 495,314 | 495,338 | At2g02060 | C2 | 7 | Plus | 495,314 | 495,338 | At2g02060 |
| C2 | 8 | Plus | 497,637 | 497,661 | At2g02060 | C2 | 8 | Minus | 598,358 | 598,334 | At2g02270 |
| C2 | 9 | Minus | 598,505 | 598,481 | At2g02270 | C2 | 9 | Minus | 831,110 | 831,086 | At2g02860 |
| C2 | 10 | Minus | 861,373 | 861,349 | At2g02960 | C2 | 10 | Plus | 1,033,902 | 1,033,926 | At2g03410 |
| C2 | 11 | Plus | 1,079,047 | 1,079,071 | At2g03560 | C2 | 11 | Plus | 1,176,468 | 1,176,492 | At2g03860 |
| C2 | 12 | Plus | 1,180,217 | 1,180,241 | At2g03870 | C2 | 12 | Plus | 1,470,208 | 1,470,232 | At2g04260 |
| C2 | 13 | Plus | 1,492,978 | 1,493,002 | At2g04300 | C2 | 13 | Plus | 1,641,084 | 1,641,108 | At2g04680 |
| C2 | 14 | Minus | 1,715,219 | 1,715,195 | At2g04870 | C2 | 14 | Plus | 1,869,692 | 1,869,716 | At2g05170 |
| C2 | 15 | Plus | 1,884,863 | 1,884,887 | At2g05200 | C2 | 15 | Minus | 2,013,447 | 2,013,423 | At2g05500 |
| C2 | 16 | Minus | 2,039,068 | 2,039,044 | At2g05550 | C2 | 16 | Minus | 2,250,709 | 2,250,685 | At2g05880 |
| C2 | 17 | Plus | 2,296,819 | 2,296,843 | At2g05950 | C2 | 17 | Minus | 2,313,228 | 2,313,204 | At2g05980 |
| C2 | 18 | Minus | 2,313,359 | 2,313,335 | At2g05980 | C2 | 18 | Minus | 2,315,601 | 2,315,577 | At2g05980 |
| C2 | 19 | Plus | 2,323,420 | 2,323,444 | At2g05990 | C2 | 19 | Plus | 2,916,774 | 2,916,798 | At2g07040 |
| C2 | 20 | Plus | 2,940,492 | 2,940,516 | At2g07090 | C2 | 20 | Plus | 4,244,000 | 4,244,024 | At2g10900 |
| C2 | 21 | Minus | 4,259,799 | 4,259,775 | At2g10940 | C2 | 21 | Plus | 4,903,820 | 4,903,844 | At2g12330 |
| C2 | 22 | Plus | 4,904,054 | 4,904,078 | At2g12330 | C2 | 22 | Plus | 4,993,067 | 4,993,091 | At2g12450 |
| C2 | 23 | Minus | 5,011,599 | 5,011,575 | At2g12470 | C2 | 23 | Minus | 5,011,599 | 5,011,575 | At2g12470 |
| C2 | 24 | Minus | 5,018,420 | 5,018,396 | At2g12480 | C2 | 24 | Plus | 5,373,193 | 5,373,217 | At2g13140 |
| C2 | 25 | Plus | 5,373,288 | 5,373,312 | At2g13140 | C2 | 25 | Plus | 5,373,288 | 5,373,312 | At2g13140 |
| C2 | 26 | Plus | 5,413,142 | 5,413,166 | At2g13180 | C2 | 26 | Minus | 5,616,003 | 5,615,979 | At2g13590 |
| C2 | 27 | Plus | 5,626,745 | 5,626,769 | At2g13620 | C2 | 27 | Minus | 5,818,913 | 5,818,889 | At2g13980 |
| C2 | 28 | Minus | 5,825,474 | 5,825,450 | At2g13990 | C2 | 28 | Minus | 5,958,901 | 5,958,877 | At2g14200 |
| C2 | 29 | Minus | 5,990,059 | 5,990,035 | At2g14260 | C2 | 29 | Minus | 6,122,914 | 6,122,890 | At2g14510 |
| C2 | 30 | Plus | 6,163,101 | 6,163,125 | At2g14560 | C2 | 30 | Plus | 6,209,005 | 6,209,029 | At2g14640 |
| C2 | 31 | Plus | 6,227,807 | 6,227,831 | At2g14680 | C2 | 31 | Minus | 6,233,926 | 6,233,902 | At2g14690 |
| C2 | 32 | Plus | 6,243,097 | 6,243,121 | At2g14700 | C2 | 32 | Plus | 6,309,005 | 6,309,029 | At2g14820 |
| C2 | 33 | Plus | 6,309,505 | 6,309,529 | At2g14820 | C2 | 33 | Plus | 6,339,729 | 6,339,753 | At2g14870 |
| C2 | 34 | Minus | 6,357,395 | 6,357,371 | At2g14910 | C2 | 34 | Minus | 6,440,570 | 6,440,546 | At2g15020 |
| C2 | 35 | Plus | 6,456,844 | 6,456,868 | At2g15040 | C2 | 35 | Minus | 6,592,440 | 6,592,416 | At2g15290 |
| C2 | 36 | Plus | 6,603,634 | 6,603,658 | At2g15310 | C2 | 36 | Minus | 6,637,102 | 6,637,078 | At2g15350 |
| C2 | 37 | Minus | 6,648,315 | 6,648,291 | At2g15370 | C2 | 37 | Plus | 6,668,410 | 6,668,434 | At2g15410 |
| C2 | 38 | Plus | 6,669,664 | 6,669,688 | At2g15410 | C2 | 38 | Minus | 6,676,733 | 6,676,709 | At2g15420 |
| C2 | 39 | Minus | 6,715,246 | 6,715,222 | At2g15510 | C2 | 39 | Minus | 6,858,938 | 6,858,914 | At2g15870 |
| C2 | 40 | Minus | 6,861,832 | 6,861,808 | At2g15870 | C2 | 40 | Minus | 7,010,665 | 7,010,641 | At2g16300 |
| C2 | 41 | Minus | 7,014,630 | 7,014,606 | At2g16310 | C2 | 41 | Plus | 7,036,616 | 7,036,640 | At2g16380 |
| C2 | 42 | Minus | 7,094,884 | 7,094,860 | At2g16490 | C2 | 42 | Minus | 7,166,187 | 7,166,163 | At2g16650 |
| C2 | 43 | Minus | 7,166,262 | 7,166,238 | At2g16650 | C2 | 43 | Minus | 7,181,027 | 7,181,003 | At2g16680 |
| C2 | 44 | Plus | 7,196,853 | 7,196,877 | At2g16710 | C2 | 44 | Plus | 7,335,321 | 7,335,345 | At2g16990 |
| C2 | 45 | Minus | 7,338,241 | 7,338,217 | At2g17000 | C2 | 45 | Minus | 7,384,468 | 7,384,444 | At2g17090 |
| C2 | 46 | Plus | 7,458,027 | 7,458,051 | At2g17265 | C2 | 46 | Minus | 7,955,393 | 7,955,369 | At2g18470 |
| C2 | 47 | Minus | 7,958,655 | 7,958,631 | At2g18480 | C2 | 47 | Plus | 8,046,937 | 8,046,961 | At2g18690 |
| C2 | 48 | Minus | 8,152,788 | 8,152,764 | At2g18940 | C2 | 48 | Minus | 8,252,658 | 8,252,634 | At2g19140 |
| C2 | 49 | Minus | 8,274,931 | 8,274,907 | At2g19190 | C2 | 49 | Minus | 8,423,149 | 8,423,125 | At2g19580 |
| C2 | 50 | Plus | 8,428,305 | 8,428,329 | At2g19600 | C2 | 50 | Plus | 8,884,458 | 8,884,482 | At2g20740 |
| C2 | 51 | Plus | 8,890,859 | 8,890,883 | At2g20750 | C2 | 51 | Plus | 9,325,926 | 9,325,950 | At2g22050 |
| C2 | 52 | Plus | 9,330,394 | 9,330,418 | At2g22060 | C2 | 52 | Plus | 9,330,394 | 9,330,418 | At2g22060 |
| C2 | 53 | Minus | 9,341,138 | 9,341,114 | At2g22100 | C2 | 53 | Minus | 9,401,797 | 9,401,773 | At2g22240 |
| C2 | 54 | Minus | 9,441,930 | 9,441,906 | At2g22340 | C2 | 54 | Plus | 9,607,936 | 9,607,960 | At2g22720 |
| C2 | 55 | Minus | 9,612,856 | 9,612,832 | At2g22740 | C2 | 55 | Minus | 10,068,843 | 10,068,819 | At2g23770 |
| C2 | 56 | Plus | 10,123,367 | 10,123,391 | At2g23900 | C2 | 56 | Plus | 10,123,367 | 10,123,391 | At2g23900 |
| C2 | 57 | Plus | 10,128,301 | 10,128,325 | At2g23910 | C2 | 57 | Plus | 10,128,301 | 10,128,325 | At2g23910 |
| C2 | 58 | Minus | 10,150,026 | 10,150,002 | At2g23980 | C2 | 58 | Minus | 10,169,366 | 10,169,342 | At2g24030 |
| C2 | 59 | Plus | 10,179,543 | 10,179,567 | At2g24060 | C2 | 59 | Plus | 10,244,810 | 10,244,834 | At2g24210 |
| C2 | 60 | Minus | 10,295,990 | 10,295,966 | At2g24330 | C2 | 60 | Minus | 10,320,735 | 10,320,711 | At2g24370 |
| C2 | 61 | Plus | 10,339,745 | 10,339,769 | At2g24440 | C2 | 61 | Minus | 10,473,189 | 10,473,165 | At2g24720 |
| C2 | 62 | Minus | 10,533,549 | 10,533,525 | At2g24850 | C2 | 62 | Minus | 10,652,600 | 10,652,576 | At2g25150 |
| C2 | 63 | Minus | 10,673,541 | 10,673,517 | At2g25180 | C2 | 63 | Plus | 10,991,208 | 10,991,232 | At2g25900 |
| C2 | 64 | Minus | 10,998,442 | 10,998,418 | At2g25910 | C2 | 64 | Minus | 10,998,442 | 10,998,418 | At2g25910 |
| C2 | 65 | Plus | 11,025,576 | 11,025,600 | At2g25970 | C2 | 65 | Plus | 11,110,477 | 11,110,501 | At2g26220 |
| C2 | 66 | Plus | 11,110,484 | 11,110,508 | At2g26220 | C2 | 66 | Minus | 11,142,694 | 11,142,670 | At2g26290 |
| C2 | 67 | Minus | 11,175,505 | 11,175,481 | At2g26380 | C2 | 67 | Plus | 11,390,670 | 11,390,694 | At2g26820 |
| C2 | 68 | Plus | 11,390,704 | 11,390,728 | At2g26820 | C2 | 68 | Plus | 11,539,500 | 11,539,524 | At2g27120 |
| C2 | 69 | Minus | 11,623,398 | 11,623,374 | At2g27280 | C2 | 69 | Plus | 11,685,155 | 11,685,179 | At2g27440 |
| C2 | 70 | Plus | 11,685,772 | 11,685,796 | At2g27440 | C2 | 70 | Plus | 11,830,059 | 11,830,083 | At2g27900 |
| C2 | 71 | Minus | 11,866,649 | 11,866,625 | At2g27980 | C2 | 71 | Minus | 11,927,748 | 11,927,724 | At2g28110 |
| C2 | 72 | Minus | 11,960,969 | 11,960,945 | At2g28180 | C2 | 72 | Minus | 12,111,291 | 12,111,267 | At2g28450 |
| C2 | 73 | Minus | 12,111,428 | 12,111,404 | At2g28450 | C2 | 73 | Minus | 12,113,927 | 12,113,903 | At2g28450 |
| C2 | 74 | Minus | 12,120,611 | 12,120,587 | At2g28470 | C2 | 74 | Minus | 12,126,355 | 12,126,331 | At2g28480 |
| C2 | 75 | Minus | 12,317,393 | 12,317,369 | At2g28830 | C2 | 75 | Plus | 12,543,489 | 12,543,513 | At2g29330 |
| C2 | 76 | Plus | 12,566,023 | 12,566,047 | At2g29410 | C2 | 76 | Minus | 12,840,710 | 12,840,686 | At2g30220 |
| C2 | 77 | Minus | 12,848,513 | 12,848,489 | At2g30240 | C2 | 77 | Minus | 12,853,226 | 12,853,202 | At2g30250 |
| C2 | 78 | Minus | 12,886,219 | 12,886,195 | At2g30360 | C2 | 78 | Minus | 12,996,751 | 12,996,727 | At2g30630 |
| C2 | 79 | Plus | 13,066,216 | 13,066,240 | At2g30780 | C2 | 79 | Plus | 13,066,216 | 13,066,240 | At2g30780 |
| C2 | 80 | Minus | 13,171,794 | 13,171,770 | At2g31080 | C2 | 80 | Plus | 13,680,911 | 13,680,935 | At2g32340 |
| C2 | 81 | Plus | 13,694,092 | 13,694,116 | At2g32370 | C2 | 81 | Minus | 13,696,052 | 13,696,028 | At2g32380 |
| C2 | 82 | Plus | 13,707,609 | 13,707,633 | At2g32410 | C2 | 82 | Plus | 14,027,692 | 14,027,716 | At2g33220 |
| C2 | 83 | Plus | 14,028,425 | 14,028,449 | At2g33220 | C2 | 83 | Minus | 14,240,198 | 14,240,174 | At2g33780 |
| C2 | 84 | Minus | 14,331,611 | 14,331,587 | At2g34050 | C2 | 84 | Minus | 14,569,544 | 14,569,520 | At2g34680 |
| C2 | 85 | Minus | 14,613,247 | 14,613,223 | At2g34760 | C2 | 85 | Plus | 15,059,822 | 15,059,846 | At2g35980 |
| C2 | 86 | Minus | 15,079,239 | 15,079,215 | At2g36040 | C2 | 86 | Minus | 15,162,137 | 15,162,113 | At2g36300 |
| C2 | 87 | Minus | 15,174,581 | 15,174,557 | At2g36310 | C2 | 87 | Plus | 15,268,280 | 15,268,304 | At2g36500 |
| C2 | 88 | Plus | 15,277,312 | 15,277,336 | At2g36550 | C2 | 88 | Minus | 15,303,105 | 15,303,081 | At2g36630 |
| C2 | 89 | Minus | 15,306,772 | 15,306,748 | At2g36640 | C2 | 89 | Minus | 15,401,826 | 15,401,802 | At2g36840 |
| C2 | 90 | Plus | 15,451,765 | 15,451,789 | At2g36910 | C2 | 90 | Plus | 15,452,605 | 15,452,629 | At2g36910 |
| C2 | 91 | Plus | 15,464,000 | 15,464,024 | At2g36950 | C2 | 91 | Plus | 15,630,788 | 15,630,812 | At2g37370 |
| C2 | 92 | Minus | 15,748,003 | 15,747,979 | At2g37670 | C2 | 92 | Plus | 15,960,810 | 15,960,834 | At2g38230 |
| C2 | 93 | Plus | 16,005,514 | 16,005,538 | At2g38320 | C2 | 93 | Plus | 16,094,726 | 16,094,750 | At2g38600 |
| C2 | 94 | Minus | 16,103,646 | 16,103,622 | At2g38630 | C2 | 94 | Plus | 16,115,973 | 16,115,997 | At2g38660 |
| C2 | 95 | Minus | 16,125,747 | 16,125,723 | At2g38690 | C2 | 95 | Plus | 16,461,820 | 16,461,844 | At2g39580 |
| C2 | 96 | Minus | 16,473,967 | 16,473,943 | At2g39640 | C2 | 96 | Plus | 17,066,819 | 17,066,843 | At2g41020 |
| C2 | 97 | Minus | 17,150,992 | 17,150,968 | At2g41250 | C2 | 97 | Minus | 17,159,674 | 17,159,650 | At2g41290 |
| C2 | 98 | Minus | 17,188,710 | 17,188,686 | At2g41370 | C2 | 98 | Minus | 17,632,615 | 17,632,591 | At2g42470 |
| C2 | 99 | Minus | 17,637,052 | 17,637,028 | At2g42480 | C2 | 99 | Plus | 18,291,239 | 18,291,263 | At2g44450 |
| C2 | 100 | Plus | 18,309,806 | 18,309,830 | At2g44480 | C2 | 100 | Plus | 18,452,090 | 18,452,114 | At2g44870 |
| C2 | 101 | Minus | 18,497,964 | 18,497,940 | At2g44970 | C2 | 101 | Plus | 18,548,195 | 18,548,219 | At2g45110 |
| C2 | 102 | Plus | 18,552,967 | 18,552,991 | At2g45120 | C2 | 102 | Minus | 18,649,168 | 18,649,144 | At2g45380 |
| C2 | 103 | Minus | 18,722,113 | 18,722,089 | At2g45550 | C2 | 103 | Minus | 18,728,939 | 18,728,915 | At2g45570 |
| C2 | 104 | Minus | 18,728,955 | 18,728,931 | At2g45570 | C2 | 104 | Plus | 19,098,781 | 19,098,805 | At2g46640 |
| C2 | 105 | Plus | 19,099,116 | 19,099,140 | At2g46640 | C2 | 105 | Plus | 19,099,116 | 19,099,140 | At2g46640 |
| C2 | 106 | Minus | 19,114,459 | 19,114,435 | At2g46680 | C2 | 106 | Minus | 19,638,347 | 19,638,323 | At2g48160 |
| C3 | 1 | Minus | 37,093 | 37,069 | At3g01015 | C3 | 1 | Minus | 276,224 | 276,200 | At3g01770 |
| C3 | 2 | Minus | 284,836 | 284,812 | At3g01790 | C3 | 2 | Minus | 434,970 | 434,946 | At3g02260 |
| C3 | 3 | Minus | 527,317 | 527,293 | At3g02520 | C3 | 3 | Minus | 702,084 | 702,060 | At3g03090 |
| C3 | 4 | Minus | 742,878 | 742,854 | At3g03220 | C3 | 4 | Minus | 825,351 | 825,327 | At3g03470 |
| C3 | 5 | Minus | 861,105 | 861,081 | At3g03580 | C3 | 5 | Plus | 967,804 | 967,828 | At3g03790 |
| C3 | 6 | Plus | 990,153 | 990,177 | At3g03855 | C3 | 6 | Minus | 1,228,511 | 1,228,487 | At3g04560 |
| C3 | 7 | Minus | 1,251,213 | 1,251,189 | At3g04610 | C3 | 7 | Plus | 1,297,067 | 1,297,091 | At3g04740 |
| C3 | 8 | Plus | 1,357,789 | 1,357,813 | At3g04910 | C3 | 8 | Plus | 1,357,789 | 1,357,813 | At3g04910 |
| C3 | 9 | Plus | 1,366,418 | 1,366,442 | At3g04940 | C3 | 9 | Plus | 1,440,303 | 1,440,327 | At3g05150 |
| C3 | 10 | Plus | 1,468,062 | 1,468,086 | At3g05170 | C3 | 10 | Plus | 1,512,447 | 1,512,471 | At3g05310 |
| C3 | 11 | Plus | 1,512,584 | 1,512,608 | At3g05310 | C3 | 11 | Plus | 1,512,584 | 1,512,608 | At3g05310 |
| C3 | 12 | Minus | 1,531,082 | 1,531,058 | At3g05360 | C3 | 12 | Minus | 2,097,992 | 2,097,968 | At3g66658 |
| C3 | 13 | Minus | 2,152,183 | 2,152,159 | At3g06820 | C3 | 13 | Minus | 3,042,966 | 3,042,942 | At3g09920 |
| C3 | 14 | Minus | 3,082,960 | 3,082,936 | At3g10010 | C3 | 14 | Plus | 3,241,171 | 3,241,195 | At3g10420 |
| C3 | 15 | Plus | 3,388,942 | 3,388,966 | At3g10820 | C3 | 15 | Plus | 3,424,269 | 3,424,293 | At3g10950 |
| C3 | 16 | Minus | 3,446,847 | 3,446,823 | At3g10990 | C3 | 16 | Minus | 3,466,903 | 3,466,879 | At3g11060 |
| C3 | 17 | Plus | 3,506,567 | 3,506,591 | At3g11180 | C3 | 17 | Plus | 3,559,116 | 3,559,140 | At3g11350 |
| C3 | 18 | Plus | 3,559,222 | 3,559,246 | At3g11340 | C3 | 18 | Plus | 3,565,280 | 3,565,304 | At3g11380 |
| C3 | 19 | Plus | 3,663,424 | 3,663,448 | At3g11590 | C3 | 19 | Minus | 3,682,870 | 3,682,846 | At3g11670 |
| C3 | 20 | Minus | 3,687,452 | 3,687,428 | At3g11680 | C3 | 20 | Minus | 3,898,663 | 3,898,639 | At3g12220 |
| C3 | 21 | Minus | 3,956,532 | 3,956,508 | At3g12470 | C3 | 21 | Minus | 4,530,590 | 4,530,566 | At3g13784 |
| C3 | 22 | Plus | 4,539,620 | 4,539,644 | At3g13800 | C3 | 22 | Plus | 4,822,488 | 4,822,512 | At3g14420 |
| C3 | 23 | Plus | 4,849,847 | 4,849,871 | At3g14450 | C3 | 23 | Minus | 4,958,278 | 4,958,254 | At3g14770 |
| C3 | 24 | Plus | 5,017,181 | 5,017,205 | At3g14910 | C3 | 24 | Plus | 5,244,568 | 5,244,592 | At3g15510 |
| C3 | 25 | Plus | 5,288,304 | 5,288,328 | At3g15605 | C3 | 25 | Plus | 5,587,511 | 5,587,535 | At3g16440 |
| C3 | 26 | Minus | 5,619,463 | 5,619,439 | At3g16520 | C3 | 26 | Minus | 5,624,772 | 5,624,748 | At3g16530 |
| C3 | 27 | Minus | 5,650,285 | 5,650,261 | At3g16590 | C3 | 27 | Plus | 5,825,386 | 5,825,410 | At3g17080 |
| C3 | 28 | Plus | 5,852,728 | 5,852,752 | At3g17160 | C3 | 28 | Plus | 5,958,825 | 5,958,849 | At3g17410 |
| C3 | 29 | Plus | 5,981,215 | 5,981,239 | At3g17470 | C3 | 29 | Minus | 6,349,176 | 6,349,152 | At3g18490 |
| C3 | 30 | Plus | 6,352,662 | 6,352,686 | At3g18500 | C3 | 30 | Plus | 6,504,405 | 6,504,429 | At3g18860 |
| C3 | 31 | Plus | 6,507,038 | 6,507,062 | At3g18860 | C3 | 31 | Plus | 6,507,038 | 6,507,062 | At3g18860 |
| C3 | 32 | Minus | 6,704,062 | 6,704,038 | At3g19340 | C3 | 32 | Plus | 6,773,570 | 6,773,594 | At3g19520 |
| C3 | 33 | Plus | 6,823,560 | 6,823,584 | At3g19650 | C3 | 33 | Plus | 6,823,560 | 6,823,584 | At3g19650 |
| C3 | 34 | Plus | 6,905,750 | 6,905,774 | At3g19860 | C3 | 34 | Plus | 6,905,750 | 6,905,774 | At3g19860 |
| C3 | 35 | Minus | 7,090,621 | 7,090,597 | At3g20330 | C3 | 35 | Minus | 7,120,539 | 7,120,515 | At3g20420 |
| C3 | 36 | Plus | 7,121,656 | 7,121,680 | At3g20430 | C3 | 36 | Plus | 7,378,541 | 7,378,565 | At3g21060 |
| C3 | 37 | Plus | 7,378,660 | 7,378,684 | At3g21060 | C3 | 37 | Plus | 7,889,644 | 7,889,668 | At3g22310 |
| C3 | 38 | Plus | 7,930,774 | 7,930,798 | At3g22400 | C3 | 38 | Plus | 7,960,213 | 7,960,237 | At3g22440 |
| C3 | 39 | Minus | 7,998,969 | 7,998,945 | At3g22560 | C3 | 39 | Minus | 8,022,047 | 8,022,023 | At3g22690 |
| C3 | 40 | Minus | 8,033,512 | 8,033,488 | At3g22740 | C3 | 40 | Minus | 8,034,528 | 8,034,504 | At3g22740 |
| C3 | 41 | Plus | 8,045,268 | 8,045,292 | At3g22760 | C3 | 41 | Plus | 8,070,279 | 8,070,303 | At3g22810 |
| C3 | 42 | Plus | 8,080,589 | 8,080,613 | At3g22830 | C3 | 42 | Minus | 8,208,648 | 8,208,624 | At3g23080 |
| C3 | 43 | Minus | 8,222,572 | 8,222,548 | At3g23110 | C3 | 43 | Minus | 8,222,572 | 8,222,548 | At3g23110 |
| C3 | 44 | Minus | 8,222,598 | 8,222,574 | At3g23110 | C3 | 44 | Minus | 8,227,425 | 8,227,401 | At3g23120 |
| C3 | 45 | Plus | 8,288,335 | 8,288,359 | At3g23220 | C3 | 45 | Minus | 8,448,934 | 8,448,910 | At3g23550 |
| C3 | 46 | Minus | 8,448,955 | 8,448,931 | At3g23550 | C3 | 46 | Minus | 8,717,322 | 8,717,298 | At3g24140 |
| C3 | 47 | Minus | 8,794,215 | 8,794,191 | At3g24260 | C3 | 47 | Minus | 8,901,190 | 8,901,166 | At3g24480 |
| C3 | 48 | Plus | 8,932,540 | 8,932,564 | At3g24510 | C3 | 48 | Plus | 9,137,582 | 9,137,606 | At3g25080 |
| C3 | 49 | Plus | 9,152,249 | 9,152,273 | At3g25130 | C3 | 49 | Plus | 9,209,170 | 9,209,194 | At3g25280 |
| C3 | 50 | Plus | 9,220,022 | 9,220,046 | At3g25420 | C3 | 50 | Plus | 9,244,281 | 9,244,305 | At3g25490 |
| C3 | 51 | Plus | 9,279,466 | 9,279,490 | At3g25545 | C3 | 51 | Minus | 9,289,223 | 9,289,199 | At3g25570 |
| C3 | 52 | Plus | 9,309,023 | 9,309,047 | At3g25600 | C3 | 52 | Plus | 9,309,023 | 9,309,047 | At3g25600 |
| C3 | 53 | Minus | 9,318,799 | 9,318,775 | At3g25620 | C3 | 53 | Minus | 9,318,799 | 9,318,775 | At3g25620 |
| C3 | 54 | Plus | 9,382,267 | 9,382,291 | At3g25720 | C3 | 54 | Minus | 9,459,575 | 9,459,551 | At3g25850 |
| C3 | 55 | Minus | 9,459,661 | 9,459,637 | At3g25850 | C3 | 55 | Minus | 9,459,661 | 9,459,637 | At3g25850 |
| C3 | 56 | Plus | 9,501,013 | 9,501,037 | At3g25960 | C3 | 56 | Plus | 9,673,833 | 9,673,857 | At3g26420 |
| C3 | 57 | Plus | 9,679,032 | 9,679,056 | At3g26440 | C3 | 57 | Minus | 9,691,309 | 9,691,285 | At3g26480 |
| C3 | 58 | Minus | 9,713,327 | 9,713,303 | At3g26510 | C3 | 58 | Minus | 9,713,327 | 9,713,303 | At3g26510 |
| C3 | 59 | Minus | 9,746,313 | 9,746,289 | At3g26540 | C3 | 59 | Minus | 9,748,461 | 9,748,437 | At3g26550 |
| C3 | 60 | Plus | 9,778,913 | 9,778,937 | At3g26610 | C3 | 60 | Plus | 9,847,508 | 9,847,532 | At3g26770 |
| C3 | 61 | Plus | 9,895,102 | 9,895,126 | At3g26840 | C3 | 61 | Plus | 10,143,107 | 10,143,131 | At3g27400 |
| C3 | 62 | Plus | 10,148,997 | 10,149,021 | At3g27420 | C3 | 62 | Minus | 10,159,596 | 10,159,572 | At3g27450 |
| C3 | 63 | Minus | 10,159,855 | 10,159,831 | At3g27450 | C3 | 63 | Minus | 10,228,459 | 10,228,435 | At3g27610 |
| C3 | 64 | Plus | 10,308,857 | 10,308,881 | At3g27810 | C3 | 64 | Plus | 10,308,857 | 10,308,881 | At3g27810 |
| C3 | 65 | Plus | 10,333,450 | 10,333,474 | At3g27870 | C3 | 65 | Plus | 10,501,316 | 10,501,340 | At3g28170 |
| C3 | 66 | Plus | 10,507,476 | 10,507,500 | At3g28180 | C3 | 66 | Minus | 10,612,600 | 10,612,576 | At3g28360 |
| C3 | 67 | Minus | 10,626,834 | 10,626,810 | At3g28380 | C3 | 67 | Minus | 10,631,000 | 10,630,976 | At3g28390 |
| C3 | 68 | Minus | 10,632,369 | 10,632,345 | At3g28390 | C3 | 68 | Plus | 10,730,546 | 10,730,570 | At3g28630 |
| C3 | 69 | Minus | 10,769,143 | 10,769,119 | At3g28700 | C3 | 69 | Minus | 10,923,196 | 10,923,172 | At3g28915 |
| C3 | 70 | Minus | 10,969,823 | 10,969,799 | At3g28940 | C3 | 70 | Plus | 11,082,353 | 11,082,377 | At3g29105 |
| C3 | 71 | Minus | 11,109,530 | 11,109,506 | At3g29150 | C3 | 71 | Plus | 11,720,949 | 11,720,973 | At3g29800 |
| C3 | 72 | Plus | 11,721,232 | 11,721,256 | At3g29800 | C3 | 72 | Plus | 11,721,807 | 11,721,831 | At3g29800 |
| C3 | 73 | Plus | 11,733,913 | 11,733,937 | At3g29820 | C3 | 73 | Plus | 11,735,100 | 11,735,124 | At3g29820 |
| C3 | 74 | Plus | 11,795,581 | 11,795,605 | At3g30170 | C3 | 74 | Plus | 12,448,568 | 12,448,592 | At3g30770 |
| C3 | 75 | Minus | 12,449,687 | 12,449,663 | At3g30775 | C3 | 75 | Minus | 12,541,004 | 12,540,980 | At3g30830 |
| C3 | 76 | Plus | 12,679,247 | 12,679,271 | At3g31310 | C3 | 76 | Plus | 14,977,636 | 14,977,660 | At3g42890 |
| C3 | 77 | Plus | 14,977,644 | 14,977,668 | At3g42890 | C3 | 77 | Minus | 15,659,933 | 15,659,909 | At3g43770 |
| C3 | 78 | Minus | 15,712,612 | 15,712,588 | At3g43850 | C3 | 78 | Plus | 15,903,837 | 15,903,861 | At3g44170 |
| C3 | 79 | Minus | 15,961,631 | 15,961,607 | At3g44260 | C3 | 79 | Minus | 15,961,635 | 15,961,611 | At3g44260 |
| C3 | 80 | Minus | 15,982,189 | 15,982,165 | At3g44290 | C3 | 80 | Plus | 16,391,818 | 16,391,842 | At3g44870 |
| C3 | 81 | Plus | 16,438,015 | 16,438,039 | At3g44960 | C3 | 81 | Plus | 16,477,512 | 16,477,536 | At3g45010 |
| C3 | 82 | Minus | 16,520,938 | 16,520,914 | At3g45120 | C3 | 82 | Minus | 16,633,330 | 16,633,306 | At3g45300 |
| C3 | 83 | Plus | 16,641,076 | 16,641,100 | At3g45320 | C3 | 83 | Minus | 16,688,580 | 16,688,556 | At3g45460 |
| C3 | 84 | Minus | 16,689,895 | 16,689,871 | At3g45470 | C3 | 84 | Plus | 17,084,422 | 17,084,446 | At3g46400 |
| C3 | 85 | Plus | 17,084,446 | 17,084,470 | At3g46400 | C3 | 85 | Minus | 17,243,912 | 17,243,888 | At3g46800 |
| C3 | 86 | Plus | 17,261,257 | 17,261,281 | At3g46840 | C3 | 86 | Plus | 17,530,145 | 17,530,169 | At3g47540 |
| C3 | 87 | Plus | 17,577,430 | 17,577,454 | At3g47640 | C3 | 87 | Minus | 17,595,404 | 17,595,380 | At3g47700 |
| C3 | 88 | Plus | 17,613,503 | 17,613,527 | At3g47740 | C3 | 88 | Plus | 17,719,709 | 17,719,733 | At3g47970 |
| C3 | 89 | Minus | 17,730,415 | 17,730,391 | At3g48010 | C3 | 89 | Minus | 17,845,975 | 17,845,951 | At3g48200 |
| C3 | 90 | Plus | 17,864,968 | 17,864,992 | At3g48230 | C3 | 90 | Plus | 18,025,237 | 18,025,261 | At3g48620 |
| C3 | 91 | Plus | 18,034,414 | 18,034,438 | At3g48650 | C3 | 91 | Plus | 18,034,414 | 18,034,438 | At3g48650 |
| C3 | 92 | Minus | 18,046,578 | 18,046,554 | At3g48690 | C3 | 92 | Minus | 18,046,578 | 18,046,554 | At3g48690 |
| C3 | 93 | Minus | 18,046,593 | 18,046,569 | At3g48690 | C3 | 93 | Plus | 18,228,288 | 18,228,312 | At3g49150 |
| C3 | 94 | Plus | 18,228,910 | 18,228,934 | At3g49150 | C3 | 94 | Plus | 18,228,910 | 18,228,934 | At3g49150 |
| C3 | 95 | Plus | 18,300,768 | 18,300,792 | At3g49330 | C3 | 95 | Plus | 18,300,768 | 18,300,792 | At3g49330 |
| C3 | 96 | Minus | 18,312,296 | 18,312,272 | At3g49360 | C3 | 96 | Minus | 18,411,010 | 18,410,986 | At3g49640 |
| C3 | 97 | Plus | 18,493,176 | 18,493,200 | At3g49840 | C3 | 97 | Plus | 18,513,678 | 18,513,702 | At3g49910 |
| C3 | 98 | Minus | 18,539,845 | 18,539,821 | At3g49980 | C3 | 98 | Minus | 18,539,845 | 18,539,821 | At3g49980 |
| C3 | 99 | Plus | 18,585,165 | 18,585,189 | At3g50090 | C3 | 99 | Minus | 18,885,763 | 18,885,739 | At3g50780 |
| C3 | 100 | Minus | 18,890,751 | 18,890,727 | At3g50790 | C3 | 100 | Plus | 18,920,998 | 18,921,022 | At3g50870 |
| C3 | 101 | Plus | 18,940,349 | 18,940,373 | At3g50930 | C3 | 101 | Minus | 19,396,840 | 19,396,816 | At3g52270 |
| C3 | 102 | Minus | 19,478,645 | 19,478,621 | At3g52510 | C3 | 102 | Plus | 19,520,211 | 19,520,235 | At3g52610 |
| C3 | 103 | Plus | 19,537,286 | 19,537,310 | At3g52680 | C3 | 103 | Plus | 19,537,286 | 19,537,310 | At3g52680 |
| C3 | 104 | Minus | 19,570,992 | 19,570,968 | At3g52780 | C3 | 104 | Minus | 19,753,572 | 19,753,548 | At3g53260 |
| C3 | 105 | Minus | 19,798,703 | 19,798,679 | At3g53380 | C3 | 105 | Minus | 20,114,604 | 20,114,580 | At3g54290 |
| C3 | 106 | Minus | 20,172,835 | 20,172,811 | At3g54460 | C3 | 106 | Plus | 20,756,822 | 20,756,846 | At3g55940 |
| C3 | 107 | Minus | 20,794,514 | 20,794,490 | At3g56000 | C3 | 107 | Plus | 21,328,265 | 21,328,289 | At3g57570 |
| C3 | 108 | Minus | 21,713,875 | 21,713,851 | At3g58670 | C3 | 108 | Minus | 21,796,400 | 21,796,376 | At3g58960 |
| C3 | 109 | Minus | 21,834,034 | 21,834,010 | At3g59050 | C3 | 109 | Minus | 21,834,034 | 21,834,010 | At3g59050 |
| C3 | 110 | Minus | 21,870,403 | 21,870,379 | At3g59130 | C3 | 110 | Plus | 22,191,298 | 22,191,322 | At3g60060 |
| C3 | 111 | Minus | 22,289,329 | 22,289,305 | At3g60280 | C3 | 111 | Minus | 22,331,277 | 22,331,253 | At3g60390 |
| C3 | 112 | Plus | 22,358,860 | 22,358,884 | At3g60490 | C3 | 112 | Plus | 22,609,682 | 22,609,706 | At3g61050 |
| C3 | 113 | Minus | 22,618,560 | 22,618,536 | At3g61090 | C3 | 113 | Plus | 22,634,341 | 22,634,365 | At3g61130 |
| C3 | 114 | Plus | 22,675,809 | 22,675,833 | At3g61240 | C3 | 114 | Minus | 22,811,292 | 22,811,268 | At3g61620 |
| C3 | 115 | Plus | 22,947,197 | 22,947,221 | At3g61940 | C3 | 115 | Plus | 22,949,177 | 22,949,201 | At3g61950 |
| C3 | 116 | Minus | 22,965,532 | 22,965,508 | At3g61980 | C3 | 116 | Minus | 22,965,532 | 22,965,508 | At3g61980 |
| C3 | 117 | Plus | 22,989,194 | 22,989,218 | At3g62060 | C3 | 117 | Minus | 22,993,046 | 22,993,022 | At3g62070 |
| C3 | 118 | Minus | 23,023,725 | 23,023,701 | At3g62160 | C3 | 118 | Plus | 23,041,814 | 23,041,838 | At3g62230 |
| C3 | 119 | Minus | 23,082,057 | 23,082,033 | At3g62360 | C3 | 119 | Minus | 23,101,050 | 23,101,026 | At3g62420 |
| C3 | 120 | Plus | 23,141,426 | 23,141,450 | At3g62530 | C3 | 120 | Plus | 23,464,829 | 23,464,853 | At3g63520 |
| C4 | 1 | Plus | 17,329 | 17,353 | At4g00010 | C4 | 1 | Plus | 209,288 | 209,312 | At4g00450 |
| C4 | 2 | Minus | 216,954 | 216,930 | At4g00480 | C4 | 2 | Minus | 217,068 | 217,044 | At4g00480 |
| C4 | 3 | Minus | 217,395 | 217,371 | At4g00480 | C4 | 3 | Minus | 405,627 | 405,603 | At4g00950 |
| C4 | 4 | Plus | 418,033 | 418,057 | At4g00970 | C4 | 4 | Plus | 440,277 | 440,301 | At4g01020 |
| C4 | 5 | Plus | 459,547 | 459,571 | At4g01060 | C4 | 5 | Plus | 642,406 | 642,430 | At4g01510 |
| C4 | 6 | Minus | 669,551 | 669,527 | At4g01540 | C4 | 6 | Plus | 701,566 | 701,590 | At4g01640 |
| C4 | 7 | Minus | 703,957 | 703,933 | At4g01650 | C4 | 7 | Minus | 703,957 | 703,933 | At4g01650 |
| C4 | 8 | Plus | 755,799 | 755,823 | At4g01750 | C4 | 8 | Plus | 929,606 | 929,630 | At4g02100 |
| C4 | 9 | Plus | 1,037,827 | 1,037,851 | At4g02350 | C4 | 9 | Plus | 1,040,291 | 1,040,315 | At4g02360 |
| C4 | 10 | Plus | 1,051,065 | 1,051,089 | At4g02390 | C4 | 10 | Plus | 1,305,743 | 1,305,767 | At4g02940 |
| C4 | 11 | Plus | 1,309,534 | 1,309,558 | At4g02940 | C4 | 11 | Plus | 1,512,425 | 1,512,449 | At4g03420 |
| C4 | 12 | Minus | 1,541,178 | 1,541,154 | At4g03470 | C4 | 12 | Minus | 1,557,337 | 1,557,313 | At4g03510 |
| C4 | 13 | Minus | 1,592,638 | 1,592,614 | At4g03570 | C4 | 13 | Plus | 2,806,067 | 2,806,091 | At4g05520 |
| C4 | 14 | Plus | 2,823,322 | 2,823,346 | At4g05550 | C4 | 14 | Minus | 3,767,792 | 3,767,768 | At4g07960 |
| C4 | 15 | Plus | 3,853,041 | 3,853,065 | At4g08040 | C4 | 15 | Minus | 4,312,287 | 4,312,263 | At4g08430 |
| C4 | 16 | Plus | 4,335,175 | 4,335,199 | At4g08450 | C4 | 16 | Plus | 4,335,175 | 4,335,199 | At4g08450 |
| C4 | 17 | Minus | 4,350,158 | 4,350,134 | At4g08470 | C4 | 17 | Plus | 4,399,133 | 4,399,157 | At4g08540 |
| C4 | 18 | Plus | 4,409,794 | 4,409,818 | At4g08550 | C4 | 18 | Minus | 4,429,915 | 4,429,891 | At4g08590 |
| C4 | 19 | Minus | 4,474,495 | 4,474,471 | At4g08630 | C4 | 19 | Plus | 4,591,392 | 4,591,416 | At4g08830 |
| C4 | 20 | Plus | 4,694,249 | 4,694,273 | At4g08930 | C4 | 20 | Minus | 4,695,530 | 4,695,506 | At4g08940 |
| C4 | 21 | Minus | 4,719,439 | 4,719,415 | At4g08970 | C4 | 21 | Plus | 4,767,088 | 4,767,112 | At4g09070 |
| C4 | 22 | Minus | 4,859,039 | 4,859,015 | At4g09290 | C4 | 22 | Minus | 4,927,005 | 4,926,981 | At4g09420 |
| C4 | 23 | Minus | 4,927,098 | 4,927,074 | At4g09420 | C4 | 23 | Plus | 5,004,243 | 5,004,267 | At4g09550 |
| C4 | 24 | Minus | 5,037,642 | 5,037,618 | At4g09600 | C4 | 24 | Minus | 5,037,642 | 5,037,618 | At4g09600 |
| C4 | 25 | Minus | 5,072,422 | 5,072,398 | At4g09670 | C4 | 25 | Plus | 5,102,240 | 5,102,264 | At4g09730 |
| C4 | 26 | Minus | 5,107,227 | 5,107,203 | At4g09740 | C4 | 26 | Plus | 5,165,146 | 5,165,170 | At4g09870 |
| C4 | 27 | Minus | 5,186,423 | 5,186,399 | At4g09900 | C4 | 27 | Plus | 5,234,323 | 5,234,347 | At4g10020 |
| C4 | 28 | Minus | 5,237,149 | 5,237,125 | At4g10030 | C4 | 28 | Minus | 5,237,149 | 5,237,125 | At4g10030 |
| C4 | 29 | Plus | 5,280,397 | 5,280,421 | At4g10120 | C4 | 29 | Plus | 5,280,816 | 5,280,840 | At4g10120 |
| C4 | 30 | Plus | 5,281,301 | 5,281,325 | At4g10120 | C4 | 30 | Minus | 5,540,364 | 5,540,340 | At4g10650 |
| C4 | 31 | Plus | 5,547,476 | 5,547,500 | At4g10660 | C4 | 31 | Plus | 5,702,642 | 5,702,666 | At4g11030 |
| C4 | 32 | Minus | 5,712,658 | 5,712,634 | At4g11050 | C4 | 32 | Plus | 5,733,939 | 5,733,963 | At4g11100 |
| C4 | 33 | Plus | 5,743,494 | 5,743,518 | At4g11120 | C4 | 33 | Plus | 5,784,602 | 5,784,626 | At4g11180 |
| C4 | 34 | Minus | 5,792,300 | 5,792,276 | At4g11200 | C4 | 34 | Minus | 5,793,840 | 5,793,816 | At4g11200 |
| C4 | 35 | Minus | 5,809,578 | 5,809,554 | At4g11230 | C4 | 35 | Plus | 5,887,641 | 5,887,665 | At4g11380 |
| C4 | 36 | Plus | 5,929,212 | 5,929,236 | At4g11460 | C4 | 36 | Plus | 5,929,338 | 5,929,362 | At4g11460 |
| C4 | 37 | Plus | 5,929,378 | 5,929,402 | At4g11460 | C4 | 37 | Plus | 5,945,159 | 5,945,183 | At4g11490 |
| C4 | 38 | Plus | 5,945,335 | 5,945,359 | At4g11490 | C4 | 38 | Minus | 6,207,157 | 6,207,133 | At4g12090 |
| C4 | 39 | Minus | 6,221,364 | 6,221,340 | At4g12120 | C4 | 39 | Plus | 6,269,712 | 6,269,736 | At4g12290 |
| C4 | 40 | Plus | 6,301,134 | 6,301,158 | At4g12390 | C4 | 40 | Plus | 6,301,134 | 6,301,158 | At4g12390 |
| C4 | 41 | Minus | 6,413,918 | 6,413,894 | At4g12580 | C4 | 41 | Minus | 6,450,923 | 6,450,899 | At4g12710 |
| C4 | 42 | Minus | 6,467,515 | 6,467,491 | At4g12750 | C4 | 42 | Minus | 6,487,677 | 6,487,653 | At4g12810 |
| C4 | 43 | Minus | 6,487,866 | 6,487,842 | At4g12810 | C4 | 43 | Plus | 6,510,923 | 6,510,947 | At4g12890 |
| C4 | 44 | Plus | 6,548,803 | 6,548,827 | At4g12960 | C4 | 44 | Minus | 6,619,936 | 6,619,912 | At4g13170 |
| C4 | 45 | Minus | 6,621,999 | 6,621,975 | At4g13180 | C4 | 45 | Minus | 6,625,568 | 6,625,544 | At4g13190 |
| C4 | 46 | Plus | 6,718,300 | 6,718,324 | At4g13320 | C4 | 46 | Minus | 6,792,541 | 6,792,517 | At4g13470 |
| C4 | 47 | Minus | 6,803,761 | 6,803,737 | At4g13490 | C4 | 47 | Plus | 6,834,902 | 6,834,926 | At4g13540 |
| C4 | 48 | Plus | 6,885,271 | 6,885,295 | At4g13610 | C4 | 48 | Plus | 6,885,318 | 6,885,342 | At4g13610 |
| C4 | 49 | Plus | 6,897,237 | 6,897,261 | At4g13620 | C4 | 49 | Minus | 6,901,751 | 6,901,727 | At4g13640 |
| C4 | 50 | Plus | 6,910,900 | 6,910,924 | At4g13660 | C4 | 50 | Minus | 7,013,159 | 7,013,135 | At4g13930 |
| C4 | 51 | Plus | 7,049,612 | 7,049,636 | At4g13990 | C4 | 51 | Minus | 7,086,969 | 7,086,945 | At4g14090 |
| C4 | 52 | Plus | 7,111,782 | 7,111,806 | At4g14140 | C4 | 52 | Plus | 7,263,071 | 7,263,095 | At4g14400 |
| C4 | 53 | Plus | 7,279,624 | 7,279,648 | At4g14460 | C4 | 53 | Minus | 7,347,336 | 7,347,312 | At4g14610 |
| C4 | 54 | Minus | 7,353,778 | 7,353,754 | At4g14620 | C4 | 54 | Minus | 7,569,340 | 7,569,316 | At4g15070 |
| C4 | 55 | Minus | 7,574,043 | 7,574,019 | At4g15080 | C4 | 55 | Minus | 7,757,747 | 7,757,723 | At4g15390 |
| C4 | 56 | Plus | 7,786,603 | 7,786,627 | At4g15417 | C4 | 56 | Plus | 7,790,288 | 7,790,312 | At4g15420 |
| C4 | 57 | Minus | 7,821,812 | 7,821,788 | At4g15500 | C4 | 57 | Minus | 7,822,048 | 7,822,024 | At4g15500 |
| C4 | 58 | Plus | 7,863,303 | 7,863,327 | At4g15570 | C4 | 58 | Minus | 7,920,048 | 7,920,024 | At4g15730 |
| C4 | 59 | Minus | 7,946,070 | 7,946,046 | At4g15780 | C4 | 59 | Plus | 8,042,782 | 8,042,806 | At4g16020 |
| C4 | 60 | Plus | 8,055,753 | 8,055,777 | At4g16045 | C4 | 60 | Plus | 8,055,753 | 8,055,777 | At4g16045 |
| C4 | 61 | Plus | 8,055,906 | 8,055,930 | At4g16045 | C4 | 61 | Minus | 8,191,144 | 8,191,120 | At4g16330 |
| C4 | 62 | Minus | 8,237,456 | 8,237,432 | At4g16442 | C4 | 62 | Minus | 8,558,751 | 8,558,727 | At4g17060 |
| C4 | 63 | Minus | 8,570,271 | 8,570,247 | At4g17090 | C4 | 63 | Minus | 8,890,457 | 8,890,433 | At4g17860 |
| C4 | 64 | Plus | 8,893,737 | 8,893,761 | At4g17870 | C4 | 64 | Plus | 8,893,737 | 8,893,761 | At4g17870 |
| C4 | 65 | Minus | 8,917,814 | 8,917,790 | At4g17910 | C4 | 65 | Minus | 8,954,275 | 8,954,251 | At4g18000 |
| C4 | 66 | Plus | 8,990,604 | 8,990,628 | At4g18050 | C4 | 66 | Plus | 9,799,150 | 9,799,174 | At4g19990 |
| C4 | 67 | Plus | 9,799,365 | 9,799,389 | At4g19990 | C4 | 67 | Plus | 10,309,722 | 10,309,746 | At4g21323 |
| C4 | 68 | Plus | 10,313,469 | 10,313,493 | At4g21326 | C4 | 68 | Minus | 10,402,983 | 10,402,959 | At4g21490 |
| C4 | 69 | Minus | 10,405,905 | 10,405,881 | At4g21500 | C4 | 69 | Minus | 10,462,747 | 10,462,723 | At4g21640 |
| C4 | 70 | Plus | 10,527,078 | 10,527,102 | At4g21760 | C4 | 70 | Plus | 10,547,587 | 10,547,611 | At4g21820 |
| C4 | 71 | Plus | 10,547,746 | 10,547,770 | At4g21820 | C4 | 71 | Plus | 10,547,746 | 10,547,770 | At4g21820 |
| C4 | 72 | Plus | 10,566,611 | 10,566,635 | At4g21860 | C4 | 72 | Minus | 10,622,192 | 10,622,168 | At4g21990 |
| C4 | 73 | Plus | 10,629,652 | 10,629,676 | At4g22010 | C4 | 73 | Plus | 10,639,797 | 10,639,821 | At4g22030 |
| C4 | 74 | Minus | 10,680,576 | 10,680,552 | At4g22120 | C4 | 74 | Plus | 10,872,566 | 10,872,590 | At4g22620 |
| C4 | 75 | Plus | 10,874,007 | 10,874,031 | At4g22630 | C4 | 75 | Minus | 10,991,793 | 10,991,769 | At4g22950 |
| C4 | 76 | Minus | 11,007,641 | 11,007,617 | At4g22970 | C4 | 76 | Minus | 11,065,736 | 11,065,712 | At4g23090 |
| C4 | 77 | Minus | 11,071,193 | 11,071,169 | At4g23100 | C4 | 77 | Plus | 11,088,074 | 11,088,098 | At4g23140 |
| C4 | 78 | Plus | 11,095,352 | 11,095,376 | At4g23160 | C4 | 78 | Minus | 11,380,642 | 11,380,618 | At4g23880 |
| C4 | 79 | Plus | 11,429,615 | 11,429,639 | At4g24000 | C4 | 79 | Plus | 11,429,615 | 11,429,639 | At4g24000 |
| C4 | 80 | Minus | 11,484,567 | 11,484,543 | At4g24110 | C4 | 80 | Minus | 11,578,510 | 11,578,486 | At4g24390 |
| C4 | 81 | Minus | 11,588,280 | 11,588,256 | At4g24410 | C4 | 81 | Plus | 11,600,390 | 11,600,414 | At4g24450 |
| C4 | 82 | Minus | 11,677,054 | 11,677,030 | At4g24620 | C4 | 82 | Plus | 11,842,300 | 11,842,324 | At4g25080 |
| C4 | 83 | Plus | 11,860,725 | 11,860,749 | At4g25120 | C4 | 83 | Minus | 11,925,236 | 11,925,212 | At4g25340 |
| C4 | 84 | Minus | 11,929,350 | 11,929,326 | At4g25350 | C4 | 84 | Plus | 11,950,849 | 11,950,873 | At4g25410 |
| C4 | 85 | Plus | 11,963,934 | 11,963,958 | At4g25430 | C4 | 85 | Minus | 12,599,022 | 12,598,998 | At4g27220 |
| C4 | 86 | Minus | 12,657,061 | 12,657,037 | At4g27350 | C4 | 86 | Minus | 12,673,480 | 12,673,456 | At4g27410 |
| C4 | 87 | Plus | 12,796,814 | 12,796,838 | At4g27720 | C4 | 87 | Plus | 13,116,187 | 13,116,211 | At4g28670 |
| C4 | 88 | Plus | 13,137,706 | 13,137,730 | At4g28710 | C4 | 88 | Minus | 13,392,737 | 13,392,713 | At4g29280 |
| C4 | 89 | Plus | 13,453,352 | 13,453,376 | At4g29490 | C4 | 89 | Plus | 13,493,525 | 13,493,549 | At4g29650 |
| C4 | 90 | Minus | 13,511,822 | 13,511,798 | At4g29710 | C4 | 90 | Plus | 13,990,194 | 13,990,218 | At4g30860 |
| C4 | 91 | Plus | 14,062,741 | 14,062,765 | At4g30993 | C4 | 91 | Plus | 14,174,976 | 14,175,000 | At4g31350 |
| C4 | 92 | Minus | 14,244,307 | 14,244,283 | At4g31520 | C4 | 92 | Minus | 14,245,441 | 14,245,417 | At4g31520 |
| C4 | 93 | Plus | 14,272,124 | 14,272,148 | At4g31580 | C4 | 93 | Plus | 14,299,064 | 14,299,088 | At4g31660 |
| C4 | 94 | Plus | 14,328,696 | 14,328,720 | At4g31740 | C4 | 94 | Plus | 14,641,431 | 14,641,455 | At4g32480 |
| C4 | 95 | Plus | 14,641,481 | 14,641,505 | At4g32480 | C4 | 95 | Plus | 14,886,890 | 14,886,914 | At4g32990 |
| C4 | 96 | Minus | 14,977,710 | 14,977,686 | At4g33200 | C4 | 96 | Plus | 15,508,988 | 15,509,012 | At4g34650 |
| C4 | 97 | Plus | 15,509,379 | 15,509,403 | At4g34650 | C4 | 97 | Minus | 15,647,328 | 15,647,304 | At4g35050 |
| C4 | 98 | Plus | 15,664,263 | 15,664,287 | At4g35080 | C4 | 98 | Minus | 15,714,131 | 15,714,107 | At4g35200 |
| C4 | 99 | Plus | 15,779,995 | 15,780,019 | At4g35370 | C4 | 99 | Plus | 15,890,441 | 15,890,465 | At4g35710 |
| C4 | 100 | Plus | 15,897,454 | 15,897,478 | At4g35730 | C4 | 100 | Plus | 15,903,248 | 15,903,272 | At4g35740 |
| C4 | 101 | Plus | 15,969,642 | 15,969,666 | At4g35900 | C4 | 101 | Plus | 15,969,642 | 15,969,666 | At4g35900 |
| C4 | 102 | Minus | 16,008,136 | 16,008,112 | At4g36020 | C4 | 102 | Plus | 16,073,059 | 16,073,083 | At4g36150 |
| C4 | 103 | Minus | 16,169,467 | 16,169,443 | At4g36430 | C4 | 103 | Minus | 16,175,753 | 16,175,729 | At4g36450 |
| C4 | 104 | Minus | 16,175,757 | 16,175,733 | At4g36450 | C4 | 104 | Minus | 16,180,175 | 16,180,151 | At4g36470 |
| C4 | 105 | Minus | 16,197,855 | 16,197,831 | At4g36520 | C4 | 105 | Plus | 16,290,523 | 16,290,547 | At4g36750 |
| C4 | 106 | Plus | 16,407,258 | 16,407,282 | At4g37000 | C4 | 106 | Minus | 16,650,980 | 16,650,956 | At4g37640 |
| C4 | 107 | Plus | 16,682,901 | 16,682,925 | At4g37710 | C4 | 107 | Plus | 16,682,901 | 16,682,925 | At4g37710 |
| C4 | 108 | Minus | 16,766,299 | 16,766,275 | At4g37860 | C4 | 108 | Minus | 16,897,089 | 16,897,065 | At4g38240 |
| C4 | 109 | Minus | 16,902,435 | 16,902,411 | At4g38260 | C4 | 109 | Minus | 16,902,435 | 16,902,411 | At4g38260 |
| C4 | 110 | Minus | 17,025,884 | 17,025,860 | At4g38640 | C4 | 110 | Minus | 17,133,354 | 17,133,330 | At4g38990 |
| C4 | 111 | Minus | 17,133,364 | 17,133,340 | At4g38990 | C4 | 111 | Minus | 17,513,480 | 17,513,456 | At4g40100 |
| C5 | 1 | Plus | 46,705 | 46,729 | At5g01010 | C5 | 1 | Minus | 168,581 | 168,557 | At5g01400 |
| C5 | 2 | Minus | 255,469 | 255,445 | At5g01680 | C5 | 2 | Minus | 343,416 | 343,392 | At5g01890 |
| C5 | 3 | Minus | 361,232 | 361,208 | At5g01930 | C5 | 3 | Minus | 374,025 | 374,001 | At5g01970 |
| C5 | 4 | Minus | 404,094 | 404,070 | At5g02050 | C5 | 4 | Minus | 509,558 | 509,534 | At5g02390 |
| C5 | 5 | Plus | 560,606 | 560,630 | At5g02510 | C5 | 5 | Plus | 703,550 | 703,574 | At5g03000 |
| C5 | 6 | Plus | 705,227 | 705,251 | At5g03010 | C5 | 6 | Plus | 727,597 | 727,621 | At5g03100 |
| C5 | 7 | Plus | 728,038 | 728,062 | At5g03100 | C5 | 7 | Plus | 919,760 | 919,784 | At5g03620 |
| C5 | 8 | Plus | 960,354 | 960,378 | At5g03680 | C5 | 8 | Minus | 1,036,239 | 1,036,215 | At5g03870 |
| C5 | 9 | Plus | 1,091,416 | 1,091,440 | At5g04040 | C5 | 9 | Plus | 1,281,514 | 1,281,538 | At5g04490 |
| C5 | 10 | Plus | 1,284,226 | 1,284,250 | At5g04500 | C5 | 10 | Plus | 1,373,236 | 1,373,260 | At5g04750 |
| C5 | 11 | Plus | 1,399,949 | 1,399,973 | At5g04820 | C5 | 11 | Plus | 1,424,436 | 1,424,460 | At5g04885 |
| C5 | 12 | Minus | 1,464,376 | 1,464,352 | At5g04970 | C5 | 12 | Minus | 1,471,804 | 1,471,780 | At5g04990 |
| C5 | 13 | Minus | 1,499,818 | 1,499,794 | At5g05080 | C5 | 13 | Minus | 1,507,709 | 1,507,685 | At5g05110 |
| C5 | 14 | Plus | 1,569,151 | 1,569,175 | At5g05290 | C5 | 14 | Plus | 1,689,352 | 1,689,376 | At5g05657 |
| C5 | 15 | Minus | 1,722,351 | 1,722,327 | At5g05730 | C5 | 15 | Minus | 1,978,400 | 1,978,376 | At5g06490 |
| C5 | 16 | Minus | 1,987,620 | 1,987,596 | At5g06520 | C5 | 16 | Plus | 2,422,860 | 2,422,884 | At5g07660 |
| C5 | 17 | Plus | 2,436,518 | 2,436,542 | At5g07680 | C5 | 17 | Minus | 2,654,639 | 2,654,615 | At5g08250 |
| C5 | 18 | Plus | 2,734,800 | 2,734,824 | At5g08460 | C5 | 18 | Minus | 3,004,020 | 3,003,996 | At5g09700 |
| C5 | 19 | Minus | 3,004,578 | 3,004,554 | At5g09700 | C5 | 19 | Minus | 3,083,255 | 3,083,231 | At5g09880 |
| C5 | 20 | Plus | 3,170,844 | 3,170,868 | At5g10120 | C5 | 20 | Minus | 3,219,320 | 3,219,296 | At5g10250 |
| C5 | 21 | Plus | 3,240,443 | 3,240,467 | At5g10300 | C5 | 21 | Plus | 3,380,547 | 3,380,571 | At5g10710 |
| C5 | 22 | Plus | 3,387,867 | 3,387,891 | At5g10720 | C5 | 22 | Plus | 3,534,164 | 3,534,188 | At5g11100 |
| C5 | 23 | Minus | 3,558,209 | 3,558,185 | At5g11180 | C5 | 23 | Plus | 3,826,201 | 3,826,225 | At5g11870 |
| C5 | 24 | Minus | 4,084,360 | 4,084,336 | At5g12930 | C5 | 24 | Minus | 4,085,181 | 4,085,157 | At5g12930 |
| C5 | 25 | Minus | 4,278,085 | 4,278,061 | At5g13340 | C5 | 25 | Minus | 4,681,146 | 4,681,122 | At5g14520 |
| C5 | 26 | Minus | 4,719,907 | 4,719,883 | At5g14640 | C5 | 26 | Minus | 4,727,815 | 4,727,791 | At5g14660 |
| C5 | 27 | Plus | 4,795,652 | 4,795,676 | At5g14830 | C5 | 27 | Plus | 4,795,652 | 4,795,676 | At5g14830 |
| C5 | 28 | Plus | 4,798,051 | 4,798,075 | At5g14830 | C5 | 28 | Plus | 4,848,521 | 4,848,545 | At5g14970 |
| C5 | 29 | Minus | 4,951,812 | 4,951,788 | At5g15250 | C5 | 29 | Minus | 5,026,495 | 5,026,471 | At5g15480 |
| C5 | 30 | Plus | 5,034,424 | 5,034,448 | At5g15510 | C5 | 30 | Plus | 5,035,654 | 5,035,678 | At5g15510 |
| C5 | 31 | Plus | 5,132,212 | 5,132,236 | At5g15730 | C5 | 31 | Minus | 5,271,433 | 5,271,409 | At5g16140 |
| C5 | 32 | Plus | 5,273,030 | 5,273,054 | At5g16150 | C5 | 32 | Minus | 5,300,111 | 5,300,087 | At5g16220 |
| C5 | 33 | Plus | 5,304,956 | 5,304,980 | At5g16230 | C5 | 33 | Minus | 6,298,655 | 6,298,631 | At5g18880 |
| C5 | 34 | Minus | 6,298,727 | 6,298,703 | At5g18880 | C5 | 34 | Plus | 6,459,572 | 6,459,596 | At5g19210 |
| C5 | 35 | Minus | 6,521,271 | 6,521,247 | At5g19360 | C5 | 35 | Plus | 6,561,124 | 6,561,148 | At5g19460 |
| C5 | 36 | Minus | 6,575,180 | 6,575,156 | At5g19490 | C5 | 36 | Minus | 6,643,429 | 6,643,405 | At5g19660 |
| C5 | 37 | Plus | 6,674,362 | 6,674,386 | At5g19740 | C5 | 37 | Minus | 6,957,565 | 6,957,541 | At5g20580 |
| C5 | 38 | Minus | 6,958,007 | 6,957,983 | At5g20580 | C5 | 38 | Minus | 7,051,563 | 7,051,539 | At5g20830 |
| C5 | 39 | Minus | 7,059,731 | 7,059,707 | At5g20840 | C5 | 39 | Minus | 7,139,834 | 7,139,810 | At5g21030 |
| C5 | 40 | Plus | 7,162,762 | 7,162,786 | At5g21090 | C5 | 40 | Plus | 7,184,647 | 7,184,671 | At5g21130 |
| C5 | 41 | Plus | 7,194,061 | 7,194,085 | At5g21150 | C5 | 41 | Plus | 7,194,061 | 7,194,085 | At5g21150 |
| C5 | 42 | Plus | 7,205,733 | 7,205,757 | At5g21170 | C5 | 42 | Plus | 7,205,733 | 7,205,757 | At5g21170 |
| C5 | 43 | Minus | 7,350,002 | 7,349,978 | At5g22280 | C5 | 43 | Plus | 7,410,178 | 7,410,202 | At5g22430 |
| C5 | 44 | Plus | 7,422,947 | 7,422,971 | At5g22470 | C5 | 44 | Plus | 7,424,509 | 7,424,533 | At5g22470 |
| C5 | 45 | Minus | 7,436,409 | 7,436,385 | At5g22490 | C5 | 45 | Plus | 7,501,293 | 7,501,317 | At5g22630 |
| C5 | 46 | Plus | 7,501,298 | 7,501,322 | At5g22630 | C5 | 46 | Minus | 7,846,615 | 7,846,591 | At5g23390 |
| C5 | 47 | Plus | 7,888,951 | 7,888,975 | At5g23460 | C5 | 47 | Minus | 7,929,717 | 7,929,693 | At5g23590 |
| C5 | 48 | Minus | 7,947,703 | 7,947,679 | At5g23660 | C5 | 48 | Minus | 7,994,628 | 7,994,604 | At5g23780 |
| C5 | 49 | Minus | 7,998,446 | 7,998,422 | At5g23800 | C5 | 49 | Plus | 8,010,359 | 8,010,383 | At5g23830 |
| C5 | 50 | Plus | 8,019,833 | 8,019,857 | At5g23860 | C5 | 50 | Plus | 8,019,833 | 8,019,857 | At5g23860 |
| C5 | 51 | Plus | 8,034,751 | 8,034,775 | At5g23890 | C5 | 51 | Plus | 8,034,751 | 8,034,775 | At5g23890 |
| C5 | 52 | Minus | 8,074,372 | 8,074,348 | At5g23980 | C5 | 52 | Minus | 8,074,387 | 8,074,363 | At5g23980 |
| C5 | 53 | Minus | 8,141,585 | 8,141,561 | At5g24140 | C5 | 53 | Minus | 8,568,389 | 8,568,365 | At5g24940 |
| C5 | 54 | Minus | 8,589,758 | 8,589,734 | At5g25000 | C5 | 54 | Plus | 8,598,194 | 8,598,218 | At5g25040 |
| C5 | 55 | Plus | 8,598,208 | 8,598,232 | At5g25040 | C5 | 55 | Minus | 8,626,054 | 8,626,030 | At5g25100 |
| C5 | 56 | Plus | 8,725,696 | 8,725,720 | At5g25250 | C5 | 56 | Plus | 8,727,141 | 8,727,165 | At5g25250 |
| C5 | 57 | Plus | 8,755,305 | 8,755,329 | At5g25290 | C5 | 57 | Plus | 8,793,087 | 8,793,111 | At5g25380 |
| C5 | 58 | Plus | 8,808,970 | 8,808,994 | At5g25410 | C5 | 58 | Plus | 8,812,543 | 8,812,567 | At5g25415 |
| C5 | 59 | Plus | 8,812,650 | 8,812,674 | At5g25415 | C5 | 59 | Plus | 8,812,847 | 8,812,871 | At5g25415 |
| C5 | 60 | Minus | 8,842,971 | 8,842,947 | At5g25470 | C5 | 60 | Minus | 8,842,971 | 8,842,947 | At5g25470 |
| C5 | 61 | Plus | 9,013,565 | 9,013,589 | At5g25900 | C5 | 61 | Plus | 9,013,876 | 9,013,900 | At5g25900 |
| C5 | 62 | Minus | 9,021,381 | 9,021,357 | At5g25920 | C5 | 62 | Minus | 9,021,381 | 9,021,357 | At5g25920 |
| C5 | 63 | Minus | 9,033,799 | 9,033,775 | At5g25950 | C5 | 63 | Plus | 9,172,894 | 9,172,918 | At5g26250 |
| C5 | 64 | Plus | 9,205,091 | 9,205,115 | At5g26300 | C5 | 64 | Plus | 9,206,749 | 9,206,773 | At5g26300 |
| C5 | 65 | Minus | 9,323,009 | 9,322,985 | At5g26670 | C5 | 65 | Minus | 9,323,009 | 9,322,985 | At5g26670 |
| C5 | 66 | Plus | 9,329,143 | 9,329,167 | At5g26680 | C5 | 66 | Minus | 9,334,519 | 9,334,495 | At5g26710 |
| C5 | 67 | Minus | 9,334,567 | 9,334,543 | At5g26710 | C5 | 67 | Plus | 9,395,850 | 9,395,874 | At5g26830 |
| C5 | 68 | Minus | 9,399,250 | 9,399,226 | At5g26840 | C5 | 68 | Minus | 9,495,173 | 9,495,149 | At5g27100 |
| C5 | 69 | Minus | 9,495,705 | 9,495,681 | At5g27110 | C5 | 69 | Minus | 9,590,324 | 9,590,300 | At5g27330 |
| C5 | 70 | Plus | 9,608,214 | 9,608,238 | At5g27350 | C5 | 70 | Plus | 9,608,214 | 9,608,238 | At5g27350 |
| C5 | 71 | Plus | 9,624,535 | 9,624,559 | At5g27370 | C5 | 71 | Plus | 9,779,465 | 9,779,489 | At5g27730 |
| C5 | 72 | Plus | 9,813,043 | 9,813,067 | At5g27810 | C5 | 72 | Plus | 9,813,043 | 9,813,067 | At5g27810 |
| C5 | 73 | Minus | 9,836,341 | 9,836,317 | At5g27870 | C5 | 73 | Minus | 9,901,021 | 9,900,997 | At5g27920 |
| C5 | 74 | Minus | 9,915,224 | 9,915,200 | At5g27930 | C5 | 74 | Minus | 9,915,224 | 9,915,200 | At5g27930 |
| C5 | 75 | Plus | 9,967,260 | 9,967,284 | At5g27970 | C5 | 75 | Plus | 9,967,260 | 9,967,284 | At5g27970 |
| C5 | 76 | Plus | 9,974,284 | 9,974,308 | At5g27990 | C5 | 76 | Minus | 10,071,484 | 10,071,460 | At5g28130 |
| C5 | 77 | Minus | 10,071,489 | 10,071,465 | At5g28130 | C5 | 77 | Plus | 10,181,571 | 10,181,595 | At5g28250 |
| C5 | 78 | Minus | 10,192,368 | 10,192,344 | At5g28260 | C5 | 78 | Plus | 10,218,913 | 10,218,937 | At5g28280 |
| C5 | 79 | Plus | 10,315,317 | 10,315,341 | At5g28410 | C5 | 79 | Minus | 10,754,976 | 10,754,952 | At5g28770 |
| C5 | 80 | Minus | 10,770,432 | 10,770,408 | At5g28780 | C5 | 80 | Plus | 13,052,553 | 13,052,577 | At5g35070 |
| C5 | 81 | Plus | 13,052,728 | 13,052,752 | At5g35070 | C5 | 81 | Plus | 13,052,728 | 13,052,752 | At5g35070 |
| C5 | 82 | Minus | 13,192,990 | 13,192,966 | At5g35210 | C5 | 82 | Plus | 13,236,083 | 13,236,107 | At5g35320 |
| C5 | 83 | Plus | 13,279,361 | 13,279,385 | At5g35340 | C5 | 83 | Plus | 13,548,594 | 13,548,618 | At5g35640 |
| C5 | 84 | Minus | 13,561,138 | 13,561,114 | At5g35655 | C5 | 84 | Minus | 13,802,937 | 13,802,913 | At5g35935 |
| C5 | 85 | Minus | 13,802,972 | 13,802,948 | At5g35935 | C5 | 85 | Minus | 13,835,814 | 13,835,790 | At5g35970 |
| C5 | 86 | Minus | 13,969,806 | 13,969,782 | At5g36220 | C5 | 86 | Plus | 14,006,924 | 14,006,948 | At5g36270 |
| C5 | 87 | Plus | 14,235,035 | 14,235,059 | At5g36870 | C5 | 87 | Plus | 14,235,035 | 14,235,059 | At5g36870 |
| C5 | 88 | Plus | 14,235,244 | 14,235,268 | At5g36870 | C5 | 88 | Minus | 14,282,109 | 14,282,085 | At5g36930 |
| C5 | 89 | Minus | 14,282,154 | 14,282,130 | At5g36930 | C5 | 89 | Plus | 14,405,485 | 14,405,509 | At5g37140 |
| C5 | 90 | Plus | 14,442,246 | 14,442,270 | At5g37200 | C5 | 90 | Plus | 14,448,078 | 14,448,102 | At5g37220 |
| C5 | 91 | Minus | 14,475,228 | 14,475,204 | At5g37280 | C5 | 91 | Minus | 14,572,614 | 14,572,590 | At5g37460 |
| C5 | 92 | Minus | 14,605,907 | 14,605,883 | At5g37500 | C5 | 92 | Plus | 14,621,859 | 14,621,883 | At5g37530 |
| C5 | 93 | Plus | 14,636,034 | 14,636,058 | At5g37560 | C5 | 93 | Minus | 14,668,425 | 14,668,401 | At5g37650 |
| C5 | 94 | Plus | 14,706,103 | 14,706,127 | At5g37740 | C5 | 94 | Minus | 14,914,256 | 14,914,232 | At5g38100 |
| C5 | 95 | Plus | 14,949,846 | 14,949,870 | At5g38190 | C5 | 95 | Plus | 15,020,332 | 15,020,356 | At5g38300 |
| C5 | 96 | Plus | 15,043,538 | 15,043,562 | At5g38350 | C5 | 96 | Plus | 15,320,943 | 15,320,967 | At5g38980 |
| C5 | 97 | Plus | 15,356,607 | 15,356,631 | At5g39080 | C5 | 97 | Minus | 15,440,794 | 15,440,770 | At5g39260 |
| C5 | 98 | Minus | 15,445,542 | 15,445,518 | At5g39280 | C5 | 98 | Minus | 15,562,719 | 15,562,695 | At5g39580 |
| C5 | 99 | Minus | 15,568,512 | 15,568,488 | At5g39600 | C5 | 99 | Minus | 15,887,725 | 15,887,701 | At5g40420 |
| C5 | 100 | Minus | 15,899,892 | 15,899,868 | At5g40450 | C5 | 100 | Minus | 15,899,962 | 15,899,938 | At5g40450 |
| C5 | 101 | Plus | 15,928,818 | 15,928,842 | At5g40480 | C5 | 101 | Plus | 15,944,452 | 15,944,476 | At5g40510 |
| C5 | 102 | Plus | 15,959,269 | 15,959,293 | At5g40560 | C5 | 102 | Minus | 15,979,120 | 15,979,096 | At5g40610 |
| C5 | 103 | Plus | 15,995,457 | 15,995,481 | At5g40650 | C5 | 103 | Plus | 16,269,531 | 16,269,555 | At5g41370 |
| C5 | 104 | Minus | 16,302,882 | 16,302,858 | At5g41450 | C5 | 104 | Minus | 16,302,882 | 16,302,858 | At5g41450 |
| C5 | 105 | Plus | 16,307,866 | 16,307,890 | At5g41470 | C5 | 105 | Minus | 16,352,782 | 16,352,758 | At5g41610 |
| C5 | 106 | Plus | 16,360,413 | 16,360,437 | At5g41620 | C5 | 106 | Minus | 16,644,196 | 16,644,172 | At5g42340 |
| C5 | 107 | Plus | 16,659,545 | 16,659,569 | At5g42390 | C5 | 107 | Plus | 16,683,025 | 16,683,049 | At5g42420 |
| C5 | 108 | Minus | 16,687,768 | 16,687,744 | At5g42440 | C5 | 108 | Minus | 16,703,142 | 16,703,118 | At5g42490 |
| C5 | 109 | Plus | 16,715,884 | 16,715,908 | At5g42520 | C5 | 109 | Minus | 16,812,248 | 16,812,224 | At5g42650 |
| C5 | 110 | Minus | 16,832,512 | 16,832,488 | At5g42690 | C5 | 110 | Minus | 16,958,339 | 16,958,315 | At5g42990 |
| C5 | 111 | Minus | 16,969,667 | 16,969,643 | At5g43020 | C5 | 111 | Minus | 17,075,641 | 17,075,617 | At5g43270 |
| C5 | 112 | Minus | 17,075,805 | 17,075,781 | At5g43270 | C5 | 112 | Minus | 17,287,955 | 17,287,931 | At5g43745 |
| C5 | 113 | Plus | 17,301,833 | 17,301,857 | At5g43760 | C5 | 113 | Plus | 17,500,913 | 17,500,937 | At5g44180 |
| C5 | 114 | Plus | 17,525,043 | 17,525,067 | At5g44220 | C5 | 114 | Minus | 17,586,564 | 17,586,540 | At5g44360 |
| C5 | 115 | Minus | 17,600,636 | 17,600,612 | At5g44400 | C5 | 115 | Plus | 17,635,557 | 17,635,581 | At5g44480 |
| C5 | 116 | Plus | 17,636,824 | 17,636,848 | At5g44480 | C5 | 116 | Plus | 17,704,743 | 17,704,767 | At5g44600 |
| C5 | 117 | Minus | 17,747,149 | 17,747,125 | At5g44700 | C5 | 117 | Plus | 17,773,680 | 17,773,704 | At5g44750 |
| C5 | 118 | Plus | 17,864,106 | 17,864,130 | At5g44940 | C5 | 118 | Plus | 17,977,548 | 17,977,572 | At5g45150 |
| C5 | 119 | Plus | 17,983,883 | 17,983,907 | At5g45160 | C5 | 119 | Minus | 18,196,479 | 18,196,455 | At5g45580 |
| C5 | 120 | Minus | 18,258,133 | 18,258,109 | At5g45720 | C5 | 120 | Minus | 18,515,537 | 18,515,513 | At5g46350 |
| C5 | 121 | Minus | 18,521,592 | 18,521,568 | At5g46360 | C5 | 121 | Plus | 18,725,467 | 18,725,491 | At5g46850 |
| C5 | 122 | Minus | 18,726,896 | 18,726,872 | At5g46860 | C5 | 122 | Minus | 18,833,706 | 18,833,682 | At5g47070 |
| C5 | 123 | Minus | 18,848,950 | 18,848,926 | At5g47110 | C5 | 123 | Minus | 19,012,989 | 19,012,965 | At5g47590 |
| C5 | 124 | Minus | 19,053,928 | 19,053,904 | At5g47750 | C5 | 124 | Plus | 19,357,507 | 19,357,531 | At5g48470 |
| C5 | 125 | Plus | 19,375,839 | 19,375,863 | At5g48520 | C5 | 125 | Minus | 19,463,665 | 19,463,641 | At5g48700 |
| C5 | 126 | Minus | 19,482,707 | 19,482,683 | At5g48740 | C5 | 126 | Minus | 19,482,707 | 19,482,683 | At5g48740 |
| C5 | 127 | Minus | 19,547,287 | 19,547,263 | At5g48910 | C5 | 127 | Plus | 19,587,583 | 19,587,607 | At5g49020 |
| C5 | 128 | Minus | 19,633,061 | 19,633,037 | At5g49140 | C5 | 128 | Minus | 19,708,319 | 19,708,295 | At5g49310 |
| C5 | 129 | Plus | 19,743,885 | 19,743,909 | At5g49390 | C5 | 129 | Plus | 19,943,244 | 19,943,268 | At5g49780 |
| C5 | 130 | Plus | 19,945,397 | 19,945,421 | At5g49780 | C5 | 130 | Minus | 19,974,673 | 19,974,649 | At5g49850 |
| C5 | 131 | Minus | 19,975,644 | 19,975,620 | At5g49850 | C5 | 131 | Plus | 20,405,231 | 20,405,255 | At5g50850 |
| C5 | 132 | Plus | 20,406,660 | 20,406,684 | At5g50850 | C5 | 132 | Minus | 20,423,064 | 20,423,040 | At5g50910 |
| C5 | 133 | Plus | 20,468,866 | 20,468,890 | At5g51050 | C5 | 133 | Plus | 20,955,349 | 20,955,373 | At5g52310 |
| C5 | 134 | Plus | 20,990,247 | 20,990,271 | At5g52410 | C5 | 134 | Plus | 21,375,802 | 21,375,826 | At5g53400 |
| C5 | 135 | Plus | 21,395,980 | 21,396,004 | At5g53430 | C5 | 135 | Plus | 21,527,923 | 21,527,947 | At5g53740 |
| C5 | 136 | Minus | 21,553,810 | 21,553,786 | At5g53780 | C5 | 136 | Plus | 21,558,005 | 21,558,029 | At5g53790 |
| C5 | 137 | Plus | 21,604,654 | 21,604,678 | At5g53910 | C5 | 137 | Plus | 21,681,561 | 21,681,585 | At5g54140 |
| C5 | 138 | Minus | 21,730,604 | 21,730,580 | At5g54230 | C5 | 138 | Plus | 21,786,187 | 21,786,211 | At5g54360 |
| C5 | 139 | Plus | 21,800,697 | 21,800,721 | At5g54390 | C5 | 139 | Plus | 21,800,895 | 21,800,919 | At5g54390 |
| C5 | 140 | Minus | 21,899,807 | 21,899,783 | At5g54610 | C5 | 140 | Minus | 21,906,543 | 21,906,519 | At5g54630 |
| C5 | 141 | Minus | 21,912,080 | 21,912,056 | At5g54650 | C5 | 141 | Plus | 22,103,870 | 22,103,894 | At5g55180 |
| C5 | 142 | Plus | 22,119,913 | 22,119,937 | At5g55240 | C5 | 142 | Minus | 22,370,453 | 22,370,429 | At5g55940 |
| C5 | 143 | Minus | 22,538,674 | 22,538,650 | At5g56360 | C5 | 143 | Minus | 22,538,674 | 22,538,650 | At5g56360 |
| C5 | 144 | Minus | 22,553,497 | 22,553,473 | At5g56380 | C5 | 144 | Plus | 22,617,662 | 22,617,686 | At5g56570 |
| C5 | 145 | Plus | 22,656,858 | 22,656,882 | At5g56700 | C5 | 145 | Minus | 22,753,698 | 22,753,674 | At5g56960 |
| C5 | 146 | Minus | 22,776,070 | 22,776,046 | At5g56990 | C5 | 146 | Plus | 22,792,807 | 22,792,831 | At5g57030 |
| C5 | 147 | Minus | 22,977,046 | 22,977,022 | At5g57400 | C5 | 147 | Plus | 23,370,405 | 23,370,429 | At5g58520 |
| C5 | 148 | Plus | 23,385,763 | 23,385,787 | At5g58560 | C5 | 148 | Minus | 23,534,622 | 23,534,598 | At5g59010 |
| C5 | 149 | Minus | 23,558,476 | 23,558,452 | At5g59060 | C5 | 149 | Minus | 23,626,503 | 23,626,479 | At5g59270 |
| C5 | 150 | Minus | 23,633,991 | 23,633,967 | At5g59300 | C5 | 150 | Plus | 24,384,232 | 24,384,256 | At5g61350 |
| C5 | 151 | Plus | 24,421,903 | 24,421,927 | At5g61440 | C5 | 151 | Plus | 24,437,543 | 24,437,567 | At5g61470 |
| C5 | 152 | Minus | 24,448,184 | 24,448,160 | At5g61500 | C5 | 152 | Plus | 24,460,912 | 24,460,936 | At5g61540 |
| C5 | 153 | Plus | 24,484,059 | 24,484,083 | At5g61610 | C5 | 153 | Minus | 24,879,483 | 24,879,459 | At5g62680 |
| C5 | 154 | Plus | 24,927,312 | 24,927,336 | At5g62780 | C5 | 154 | Minus | 25,106,094 | 25,106,070 | At5g63390 |
| C5 | 155 | Plus | 25,167,332 | 25,167,356 | At5g63570 | C5 | 155 | Plus | 25,206,130 | 25,206,154 | At5g63680 |
| C5 | 156 | Minus | 25,230,472 | 25,230,448 | At5g63760 | C5 | 156 | Minus | 25,466,367 | 25,466,343 | At5g64410 |
| C5 | 157 | Minus | 25,476,773 | 25,476,749 | At5g64430 | C5 | 157 | Minus | 25,476,773 | 25,476,749 | At5g64430 |
| C5 | 158 | Plus | 25,480,714 | 25,480,738 | At5g64440 | C5 | 158 | Plus | 25,480,714 | 25,480,738 | At5g64440 |
| C5 | 159 | Plus | 25,490,238 | 25,490,262 | At5g64470 | C5 | 159 | Plus | 25,782,500 | 25,782,524 | At5g65230 |
| C5 | 160 | Plus | 25,782,798 | 25,782,822 | At5g65230 | C5 | 160 | Minus | 25,800,137 | 25,800,113 | At5g65280 |
| C5 | 161 | Minus | 25,816,742 | 25,816,718 | At5g65310 | C5 | 161 | Minus | 25,912,871 | 25,912,847 | At5g65550 |
| C5 | 162 | Plus | 25,969,701 | 25,969,725 | At5g65670 | C5 | 162 | Plus | 25,969,701 | 25,969,725 | At5g65670 |
| C5 | 163 | Plus | 25,981,997 | 25,982,021 | At5g65690 | C5 | 163 | Plus | 25,982,235 | 25,982,259 | At5g65690 |
| C5 | 164 | Plus | 26,019,115 | 26,019,139 | At5g65750 | C5 | 164 | Minus | 26,132,720 | 26,132,696 | At5g66055 |
| C5 | 165 | Plus | 26,133,530 | 26,133,554 | At5g66060 | C5 | 165 | Minus | 26,188,008 | 26,187,984 | At5g66270 |
| C5 | 166 | Minus | 26,228,028 | 26,228,004 | At5g66380 | C5 | 166 | Plus | 26,425,292 | 26,425,316 | At5g66880 |
| C5 | 167 | Minus | 26,434,548 | 26,434,524 | At5g66910 | C5 | 167 | Minus | 26,681,836 | 26,681,812 | At5g67640 |

**Table 9:** This table lists the genes marking the beginning and end of each recombination interval for the 98 lines of the Col-0/L*er* RIL population. The SFP-probe position for each gene is indicated.
