# Supplementary material for: A High-Resolution Map of Arabidopsis Recombinant Inbred Lines by Whole-Genome Exon Array Hybridization
Source: PLoS Genet. 2006 Sep 15;2(9):e144. doi: 10.1371/journal.pgen.0020144 (PMC1564425; doi:10.1371/journal.pgen.0020144)
Supplement: Supplementary file 19 [file pgen.0020144.st010.doc]

|  | Max. recombination rate | |  | Min. recombination rate | |  | Ave. recombination rate (slope) | | |
| --- | --- | --- | --- | --- | --- | --- | --- | --- | --- |
| Chrom. | (cM/50kb) | (kb/cM) | (cM/Mb) | (cM/50kb) | (kb/cM) | (cM/Mb) | (cM/50kb) | (kb/cM) | (cM/Mb) |
| 1 | 7.90 | 6.33 | 158.0 | 0.022 | 2,319 | 0.431 | 0.176 | 285 | 3.5 |
| 2 | 5.07 | 9.87 | 101.3 | 0.025 | 2,037 | 0.491 | 0.168 | 298 | 3.4 |
| 3 | 10.25 | 4.88 | 205.0 | 0.017 | 2,981 | 0.335 | 0.162 | 308 | 3.2 |
| 4 | 6.26 | 7.99 | 125.1 | 0.015 | 3,226 | 0.310 | 0.198 | 252 | 4.0 |
| 5 | 12.55 | 3.98 | 251.0 | 0.017 | 3,013 | 0.332 | 0.176 | 285 | 3.5 |
| Ave |  |  |  |  |  |  | 0.176 | 285.5 | 3.5 |

**Supplemental Table 10.**

Recombination rates for 5 *Arabidopsis* chromosomes.

For estimating recombination rates we compared the genetic distance (cM) with the physical distance between map units, normalized to a 50kb window size. For each genetic interval an average physical position was calculated (SFP-marker position at the beginning of interval + SFP-marker position at the end of the interval/(2*50kb).

The average recombination rate was calculated as the slope of a regression-line fitted through the cumulative cM distances between informative SFP-markers plotted on the y-axis and and physical marker position plotted on the x-axis (SFP-marker position at the beginning of interval + SFP-marker position at the end of the interval/(2*50kb) (Fig. S9-S11, panel B).
